# Predicting DNA Reactions with a Quantum Chemistry‐Based Deep Learning Model

**DOI:** 10.1002/advs.202409880

**Published:** 2024-09-19

**Authors:** Likun Wang, Na Li, Mengyao Cao, Yun Zhu, Xiewei Xiong, Li Li, Tong Zhu, Hao Pei

**Affiliations:** ^1^ Shanghai Key Laboratory of Green Chemistry and Chemical Processes Shanghai Engineering Research Center of Molecular Therapeutics and New Drug Development School of Chemistry and Molecular Engineering East China Normal University Shanghai 200241 China; ^2^ Shanghai Innovation Institute Shanghai 200003 China; ^3^ Institute for Advanced Algorithms Research Shanghai 200062 China

**Keywords:** deep learning, DNA reactions, quantum chemistry

## Abstract

In this study, a deep learning model based on quantum chemistry is introduced to enhance the accuracy and efficiency of predicting DNA reaction parameters. By integrating quantum chemical calculations with self‐designed descriptor matrices, the model offers a comprehensive description of energy variations and considers a broad range of relevant factors. To overcome the challenge of limited labeled data, an active learning method is employed. The results demonstrate that this model outperforms existing methods in predicting DNA hybridization free energies and strand displacement rate constants, thus advancing the understanding of DNA molecular interactions, and aiding in the precise design and optimization of DNA‐based systems.

## Graphical abstract

In this work, a quantum chemistry‐based deep learning model is proposed to enhance the accuracy and efficiency of predicting DNA reaction parameters. By integrating quantum chemistry into self‐designed descriptor matrices, it offers a comprehensive description of energy variations. Employing an active learning method addresses limited labeled dataset, yielding superior predictions over existing methods.

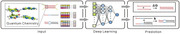

## 1 Introduction

DNA reactions play a pivotal role in numerous biological processes, synthetic biology applications, and emerging fields like DNA computing.^[^ ^1^ ^]^ Accurate prediction of the thermodynamic and kinetic parameters of these reactions is crucial for understanding molecular interactions, designing functional DNA‐based systems, and advancing various technological applications.^[^ ^2^ ^]^ Existing software for predicting thermodynamics of DNA reactions such as Mfold and Nupack rely on the nearest neighbor model.^[^ ^3^ ^]^ The model calculates the free energy of DNA molecule formation as the sum of non‐overlapping independent motifs, including stacks, dangles and loops.^[^ ^4^ ^]^ The predictions exhibit deviations from experimental measurements, with errors in the predicted melting temperature reaching up to 1.6 °C.^[^ ^5^ ^]^ This discrepancy stems from several factors: 1) inaccuracies in experimental thermodynamic measurements; 2) the limited accuracy of deriving free energies of stacks due to mathematical approximations; 3) limitations in the linear summation approach of all motifs within the nearest neighbor model. Recent advancements by Wang and Bae et al. have improved the accuracy of experimental thermodynamic measurements for dangles and loops using noncovalent catalysis and toehold exchange energy measurement, addressing the first problem.^[^ ^6^ ^]^ However, the limitations inherent in the nearest neighbor model remain, along with the challenges in measuring and estimating thermodynamic parameters of stacks. The next‐nearest neighbor model proposed by Owczarzy et al. seeks to solve the third problem, but it has not significantly improved prediction accuracy.^[^ ^7^ ^]^

Machine learning has demonstrated great potential in discovering patterns and rules within large data, making it a valuable tool for enhancing scientific models to address intricate problems involving nonlinear processes or broad chemical spaces.^[^ ^8^ ^]^ For instance, Zhang et al. proposed two machine learning models: a weighted neighbor voting model and a deep learning model.^[^ ^9^ ^]^ These models were specifically designed to predict DNA hybridization and strand displacement rate constants based on sequences and experimental conditions. Impressively, they achieved a significant 50% reduction in root mean square error compared to traditional approaches such as the three‐step model and worm‐like chain model.^[^ ^10^ ^]^ Despite these advancements, machine learning models predicting DNA reaction thermodynamics demand specific physical information about DNA, leading to the requirement of extensive training data. Notably, quantum chemical calculations can provide precise descriptions of electronic structure and atomic behavior. For example, Hopfinger et al. demonstrated significant agreement between energy calculations obtained from quantum chemical calculations and parameters derived from a series of optical melting experiments. Their approach successfully predicted the thermodynamic parameters of modified nucleotides.^[^ ^11^ ^]^ However, relying solely on quantum chemical calculations for predicting DNA reaction thermodynamics demands huge computational resources and will crash when applied for such large systems. The integration of quantum chemistry with machine learning offers a possible solution. Incorporating these two approaches, quantum chemical calculations supply the model with detailed physical information about DNA, thereby reducing the dependency on extensive data. Additionally, this integration can enhance the model's generalizability and predictive ability outside the training data. This integrated approach has shown notable success in predicting chemical properties and reaction pathways.^[^ ^12^ ^]^

**Figure 1:**
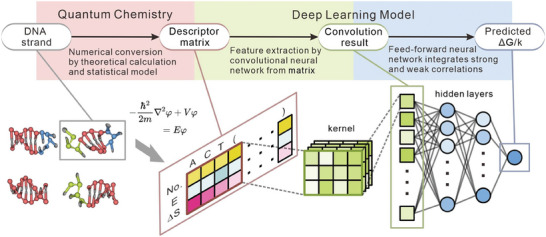
The scheme used in this study. The given DNA strands undergo a preprocessing stage where they are transformed into descriptor matrices. Then these descriptor matrices serve as inputs for a convolutional neural network. Subsequently, the convolution results are fed into a feed‐forward neural network, ultimately producing the desired output.

In this study, we proposed a quantum chemistry‐based deep learning model to predict the thermodynamic and kinetic parameters of DNA reactions with enhanced accuracy and efficiency (**Figure** **1**). This model integrates quantum chemical calculations, self‐designed descriptor matrices, and deep learning algorithms. It comprehensively describes the energy variations associated with DNA reactions by expanding stacking terms and incorporating other relevant factors. Quantum chemical calculations are used to obtain energy values, while a physical motivated statistical model estimates entropy values. Furthermore, to overcome the challenge of limited labelled datasets, we employed an active learning method,^[^ ^13^ ^]^ which iteratively selects informative samples of DNA reactions. This approach not only enhances the model's performance but also optimizes the utilization of available data on DNA reactions. Through extensive evaluations and comparisons with existing models, we demonstrated the superior predictive capability of our model in accurately predicting DNA hybridization free energies and strand displacement rate constants. These results highlight the potential of integrating quantum chemistry with deep learning for advancing our understanding of DNA reactions and enabling more precise design and optimization of DNA‐based systems.

## 2 Experimental Section

### 2.1 Design of Descriptor Matrices

**Figure 2:**
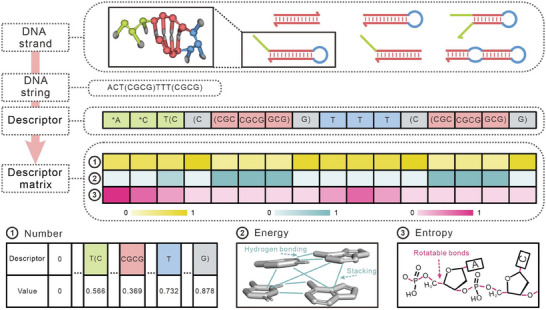
Design of descriptor matrices. The descriptors encompass stacking terms (roman), dangling terms (conifer), looping terms (danube), and initiating terms (iron). The first row of the descriptor matrices denotes “Number,” and the values presented in the table are normalized. The second row signifies “Energy,” which is the sum of the stacking interaction energy and the hydrogen bond interaction energy of bases, prominently displayed as neptune lines in the panel. The third row highlights “Entropy”, where rotatable bonds are depicted as cerise lines.

Due to the lack of critical structural and interaction information, using DNA sequences directly as inputs for deep learning models makes it difficult to achieve ideal prediction accuracy. In this study, a novel method is proposed for preprocessing DNA strands into descriptor matrices, which involves three main steps: converting DNA strands into DNA strings, transforming DNA strings into descriptors and constructing descriptors matrices (**Figure** **2**).

First, inspired by the simplified molecular input line entry system (SMILES) and dot‐parens‐plus notation for nucleic acid molecules,^[^ ^3^, ^14^ ^]^ DNA strands were converted into custom DNA strings. The conversion follows four rules: 1) Nucleotides are represented by their respective abbreviation “A”, “T”, “C”, “G”; 2) Sequences begin at the 5′ and end at the 3′; 3) Parentheses “()” indicate that the nucleotides within are paired, while those outside are unpaired; 4) Dot “.” represents the end of the first strand in a double‐stranded DNA.

Secondly, DNA strings were transformed into descriptors. The descriptors encompass four types: 1) Stacking term: the parentheses are only considered and the characters they enclose. Starting with the left parenthesis, every 4 characters constitute a string. For example, the string “(CGCG)” would be transformed into three quartets: “(CGC”, “CGCG”, and “GCG)”. There are a total of 400 possible quartets. This is where this approach improves on the nearest neighbor model, because combinations of 4 nucleotides are considered instead of 2, providing a more accurate description of their physical interactions. 2) Dangling term: In a contiguous sequence, all nucleotides between the start and the left parenthesis or between the right parenthesis and the end, are considered to be dangles. Starting from either the beginning or the end, for each nucleotide in the dangle, a “*” is added to its left if it is not immediately adjacent to a parenthesis. However, if the nucleotide is next to a parenthesis, instead of adding a “*”, the parenthesis and the first nucleotide within it are included. For example, the string “CT(C” can be transformed into two sub‐strings: “*C” and “T(C.” There are 36 dangling terms in total. 3) Looping term: Nucleotides that lie between two sets of parentheses should be considered part of a loop region and are directly represented by their corresponding single characters. 4) Initiating term: The first and last nucleotides in the stack domain are defined as the initiating term, represented as “(X”, “X)”. The “.” is also considered as an initiating term.

Third, the descriptors were transformed into descriptor matrices, which have three features: “Number,” “Energy,” and “Entropy.” “Number” is the identifier that is assigned to different terms in the descriptors, with detailed correspondences provided in Table S1 (Supporting Information). The“Energy” values were calculated as the sum of all pairwise interactions between nucleotides within each term. These values were obtained through a combination of molecular dynamics simulations and quantum chemical calculations. Details are described in the next section. The “Entropy” values were derived based on the physically motivated statistical model, calculated as the product of the entropy coefficient and the number of rotatable bonds in the nucleotide.^[^ ^15^ ^]^ Unpaired nucleotides have multiple rotatable bonds, including carbon–nitrogen bonds between pentoses and bases, carbon–oxygen bonds, carbon–carbon bonds, and phosphorus‐oxygen bonds on the phosphoric acid backbone. For dangling terms and looping terms, the entropy coefficient decreases as it approaches to a stack domain. Thus, the entropy coefficient of the first nucleotide near the stack domain was set to 0.33, the second nucleotide to 0.67, and subsequent nucleotides to 1. For stacking and initiating terms, the entropy coefficient was set to 0 due to structural constraints. Due to the different lengths of descriptors for DNA duplexes, zero‐padding was used for the shorter descriptors to match the length of the longest descriptor.

### 2.2 Quantum Chemical Calculations

A total of stacking terms are 400, including 256 stacking terms in the middle of the strand, 128 stacking terms at the end of the strand, and 16 stacking terms with left and right parentheses. Among them, there are 136 unique stacking terms in the middle of the strand and 64 unique stacking terms at the end of the strand that need to be calculated. For example, the duplexes 5′‐TGCT‐3′/3′‐ACGA‐5′ and 5′‐AGCA‐3′/3′‐TCGT‐5′ are equivalent. Additionally, a total of dangling terms are 36, only the dangling terms with parentheses need to be considered. That is, there are 32 dangling terms need to calculate.

**Figure 3:**
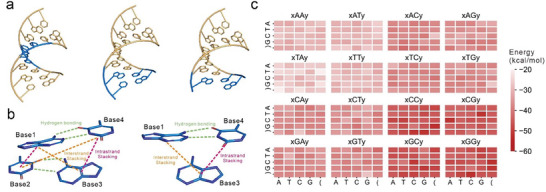
Structures and results of quantum chemical calculations. a) Structures of stacking terms in the middle of the strand, stacking terms at the end of the strand and dangling terms. b) Schematic of bases involved for calculated hydrogen bonding energies and stacking energies in stacking terms and dangling terms. c) An overview of energies for 400 stacking terms. The stacking terms’ *x* and *y* correspond to the characters delineated along the x and y axes, respectively, with sequences presented in the direction of 5′ to 3′.

For each duplex, the nucleic acid builder (NAB) in AmberTools18 (https://ambermd.org) was used to build its initial structure. One strand of the DNA duplex, containing stacking terms either in the middle or at the end, was designed as 5′‐GCZXYZGC‐3′ or 5′‐GCGCZXY‐3′, respectively, with the other strand being the Watson‐Crick base pairing complement. XY represents the stacking terms of the target, and Z can be any nucleotide (**Figure** **3**a). One strand of the duplex with dangling terms was designed as 5′‐GCGCGXY‐3′ or 5′‐YXGCGCG‐3′, and the other strand was complementary to the sequence excluding the nucleotide Y. Y can be any nucleotide at the 3′ or the 5′ end.

A constructed DNA duplex was subjected to molecular dynamics simulations using OL15 force field.^[^ ^16^ ^]^ The duplex was solvated in an octahedral box using TIP3P water molecules with a 12.0 Å buffer, and neutralized with sodium ions. Then energy minimization was carried out for 10 000 steps, utilizing a force constant of 100 kcal (mol Å^2^)^−1^ to constrain the duplex. Subsequently, a second 10000‐step energy minimization was performed without any restraints. After minimization, the system was gradually heated from 0 to 310 K within 20 ps, keeping a weak force constant of 10 kcal (mol Å^2^)^−1^ to constrain the DNA duplex with NVT ensemble. Then an unrestrained equilibration was performed for 100 ps in the NPT ensemble under 1 atm and 310 K. Once the system reached equilibrium, the final molecular dynamics simulation was run for 5.0 ns with the same equilibration settings. The timestep was set to 2 fs and a Langevin thermostat was used to control the temperature with the collision frequency of 1.0 ps^−1^. The non‐bonded cutoff was set to 12.0 Å and the Particle Mesh Ewald (PME) algorithm was applied to treat the long‐range electrostatic interactions. Hydrogen atoms were constrained with the SHAKE algorithm.

The DNA duplex was clustered into two conformations from the 5 ns molecular dynamics trajectory with water molecules and sodium ions removed, using the hierarchical clustering method of Cpptraj program. The clustered conformations had their backbone atoms and non‐target nucleotides removed, and hydrogen atoms were then added to the N1 position of the pyrimidine base and the N9 position of the purine base. The remaining residues were performed quantum chemical calculations. Single point energy was calculated using Orca 4.1.2 (https://orcaforum.kofo.mpg.de) with ωB97X‐D3 level of theory,^[^ ^17^ ^]^ def2‐TZVP basis set,^[^ ^18^ ^]^ and def2‐TZVP/C auxiliary basis set. The solvation model was the conductor‐like polarized continuum model, and the solvent probe radius was 1.5 Å to overcome the inconsistency caused by implicit solvent calculations.

The “Energy” *E* is calculated according to the following equation:

(1) $$E\;=E_{\mathrm{HB}}\;+E_{\mathrm{stack}}$$

For each stacking term, six interactions were considered: two base pairing hydrogen bonding energies, two intra‐stand stacking energies and two inter‐strand stacking energies. In Figure 3b, Base1‐Base4 and Base2‐Base3 are base pairs, and the energy of stacking and hydrogen bonding can be calculated as the sum of their interaction energies (*E*_int_), where 1, 2, 3, and 4 represent Base1, Base2, Base3 and Base4, respectively. The equations are as follows:

(2) $$E_{\mathrm{HB}}=E_{\mathrm{int},1-4}\;+E_{\mathrm{int},2-3}$$

(3) $$E_{\mathrm{stack}}=E_{\mathrm{int},1-2}\;+E_{\mathrm{int},3-4}+E_{\mathrm{int},1-3}+E_{\mathrm{int},2-4}$$

For each dangling term, three interactions were considered: a base pairing hydrogen bonding energy, an intrastrand stacking energy and an interstrand stacking energy. The equation is as follows:

(4) $$E_{\mathrm{stack}}=E_{\mathrm{int},1-3}\;+E_{\mathrm{int},3-4}$$

Each interaction energy is corrected by the balance method of Boys and Bernardi which minimizes the basis set superposition error.^[^ ^19^ ^]^ For example, the equation for calculating *E*_int,1‐2_ is as follows:

(5) $$E_{\mathrm{int},1-2}=E_{1,2}^{1,2,3,4}\;-E_{1}^{1,2,3,4}-E_{2}^{1,2,3,4}$$

Finally, for the same type of interaction energy, the mean energy of the two clustered conformations was used. Interaction energies of 200 stacking terms and 32 dangling terms are provided in Tables S2 and S3 (Supporting Information), respectively. The energy value for each stacking term with left and right parentheses was calculated as the mean energy of all stacking terms involving the same two nucleotides in the middle. For example, in the “xCGy” stacking term, “x” and “y” can be any one of “A,” “T,” “C,” “G,” or “(”, resulting in 25 possible combinations. The energy value of “(CG)” is equal to the mean energy of the remaining 24 combinations. The values of “Energy” feature for 400 stacking terms and 32 dangling terms are provided in Table S4 (Supporting Information). The energies of remaining dangling terms, looping terms, and initiating terms were set to 0. The energy values of 400 stacking terms were found to range from −52.174 to −20.615 kcal mol^−1^ (Figure 3c).

### 2.3 Model Architecture

The normalized descriptor matrices were fed into a convolutional neural network with a kernel size of *N* × 4 (height × weight), where *N* is the number of rows in the descriptor matrices. The output of the convolution layer was flattened and expanded into a 1D vector, which served as the input to a feed‐forward neural network. The feed‐forward neural network contained two hidden layers with 256 and 128 nodes respectively.

Before training, weight parameters in the model were randomly initialized using the Xavier initialization method. The dataset was randomly divided into a training set and a validation set in a ratio of 8:2. During training, the Adam optimizer was employed to minimize the loss function and update weight parameters, with the loss defined as the mean squared error between the predicted and experimental values. To minimize overfitting, additional dropout layers were added after the convolution layer and each hidden layer. Additional hyperparameters included a batch size of 999 and a learning rate of 0.001. The early stopping method was used to obtain the best trained model. All models were implemented using Keras (https://keras.io/).

## 3 Results and Discussion

### 3.1 Prediction of DNA Hybridization Free Energies

**Figure 4:**
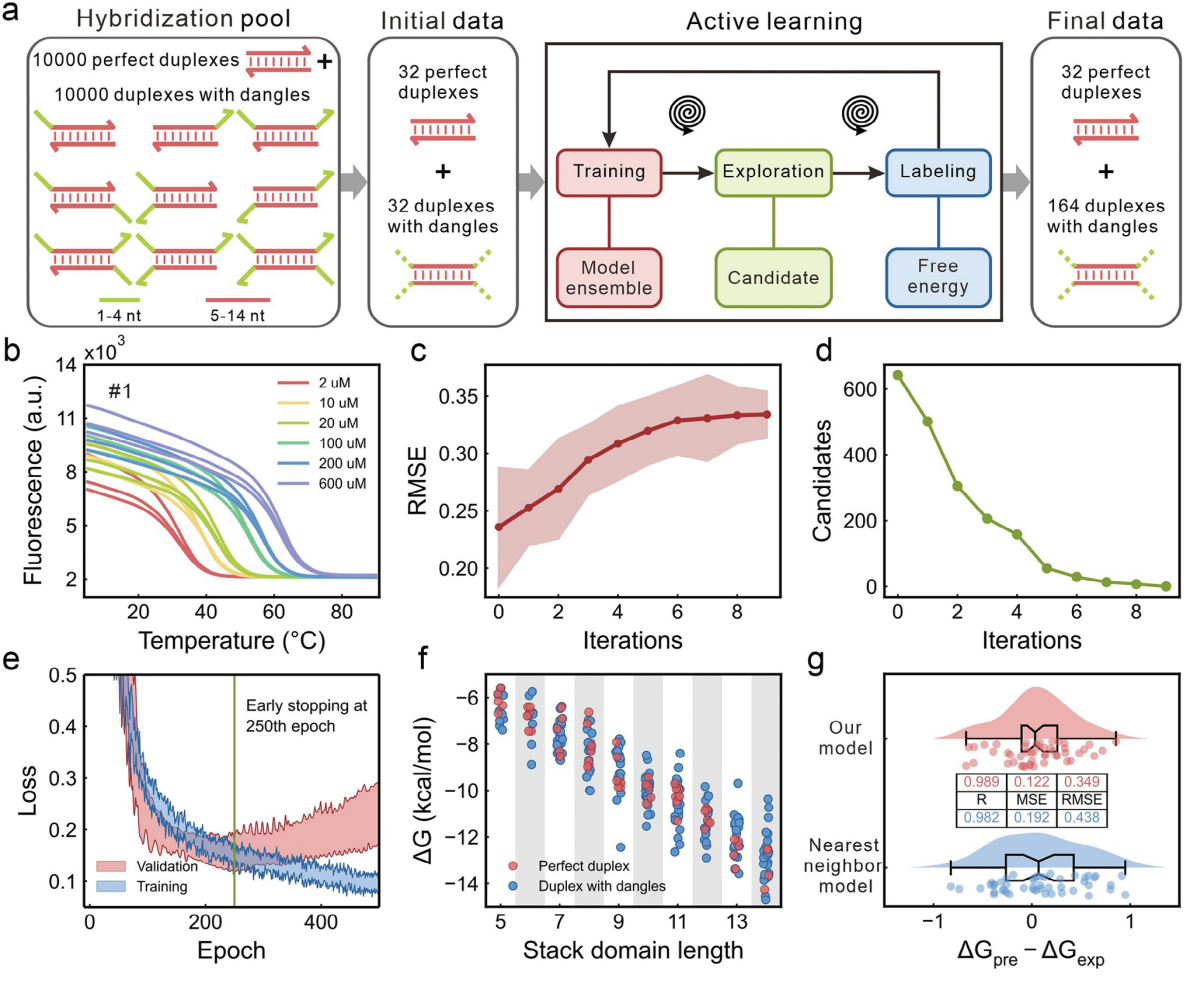
Results of model training and DNA hybridization free energies prediction. a) DNA hybridization dataset constructed using the active learning method. b) Example of HRM curves for a duplex. c) RMSE of models on the training set with increasing iterations during active learning. The shaded red area delineates the upper and lower limit of the models' RMSE on the training set. The red points represent the mean values. d) The number of candidates versus the number of iterations during active learning. e) The loss versus training epoch for four independently trained models in the last iteration. The shaded blue and red areas delineate the upper and lower limits of the models' loss on the training and validation sets, respectively. Model training was halted at 250 epochs. f) Experimental hybridization free energies versus the stack domain length for all duplexes. g) Benchmark of prediction accuracy for our model and the nearest neighbor model.

Here, we used our model to predict DNA hybridization free energies. To construct a hybridization dataset, a hybridization pool was established, including 10 000 perfect duplexes and 10 000 duplexes with dangles (**Figure** **4**a). Short duplexes tend to denature at room temperatures, rendering their melting temperatures unmeasurable. Long duplexes may adopt one or more secondary structures in their single strand, and their melting temperatures at different concentrations are close or even overlapped, leading to biased experimental measurements. Consequently, the length of the stack domain was designed ranging from 5 to 14 nucleotides. Since the free energy of dangles with more than 4 nucleotides can be approximated to that of dangles with 4 nucleotides,^[^ ^6a^ ^]^ the length of the dangle domain was designed ranging from 1 to 4 nucleotides. Given the possibility of dangles positions in duplexes, a total of ten types of duplexes are considered in the pool. High resolution melting (HRM) is used to measure DNA hybridization thermodynamics parameters since it requires lower DNA concentrations compared to isothermal titration calorimetry and offers more efficient data acquisition than ultraviolet melting.^[^ ^20^ ^]^ HRM analysis involves measuring melt curves of duplexes using the real‐time fluorescent quantitative PCR (Figure 4b) and determined hybridization free energies by fitting analysis. These determined free energies serve as labels for hybridization dataset; see Section S2 (Supporting Information) for details.

To enhance the efficiency of data collection and reduce experimental costs, the active learning method was adopted. A total of 64 duplexes were randomly selected from the hybridization pool as the initial hybridization dataset, including 32 perfect duplexes and 32 duplexes with dangles. After obtaining hybridization free energies of these duplexes (Figure S2, Supporting Information), four machine learning models with different initialization of weight parameters *ω* were trained. Then these four models were used to predict hybridization free energies of remaining duplexes in the pool. The maximal standard deviation for these models is defined as the error indicator, denoted as *ϵ*:

(6) $$\varepsilon \;=\max_{i}\;\left(\sqrt{{\left(G_{\omega ,i}-\left\langle G_{\omega ,i}\right\rangle \right)}^{2}}\right)$$

where *G*_ω,*i*_ is the hybridization free energy predicted by the model i, and 〈*G*_ω,*i*_〉 is the expectation of four models. We set a threshold for *ϵ* at 0.6. During each iteration of active learning, duplexes with *ϵ* values greater than the threshold were considered as candidates that need to be labeled. Selection was made based on the magnitude of deviations, from the largest to the smallest, and up to 16 duplexes were selected in each iteration. After measuring hybridization free energies of these candidates, they were added to the dataset for subsequent retraining. Throughout the iterative process of the active learning, as the number of iterations increased, the mean RMSE of the four models on the training set gradually rose from the initial value of 0.236 to 0.334 (Figure 4c), indicating that the models were trained on increasingly challenging and informative data.^[^ ^21^ ^]^ Meanwhile, the number of candidates steadily decreased from an initial count of 642 to 0 (Figure 4d). A total of 132 duplexes were selected during the active learning process (Figure S3, Supporting Information). Consequently, the hybridization dataset comprised 196 duplexes, including the initial 64 duplexes. In the final iteration of training, all four models achieved the early stopping criteria, indicating they were all sufficiently trained (Figure 4e). Thus, we selected the model that performed the best on the validation set for evaluation.

A total of 196 duplexes were used in the final hybridization dataset, with 157 duplexes in the training set and 39 duplexes in the validation set. To evaluate the model's performance, a test set consisting of 48 duplexes was established. The 48 duplexes comprised 24 perfect duplexes and 24 duplexes with dangles (Figure S4, Supporting Information). For these perfect duplexes, the stack domain ranged from 5 to 14 nucleotides. For these duplexes with dangles, the stack domain ranged from 5 to 14 nucleotides and the dangle domain spanned 1 to 4 nucleotides, covering 9 possible dangle positions. Experimental results show that the distribution of hybridization free energies for all duplexes ranged from −14.7 to −5.58 kcal mol^−1^ (Figure 4f).

To demonstrate the predictive ability of our model, it is necessary to benchmark it against the nearest neighbor model. The nearest neighbor model incorporates 44 parameters, including 2 initiations, 10 stacks, and 32 dangles, all derived through multiple linear regression.^[^ ^22^ ^]^ We used this model to predict hybridization free energies of the same test set. The results show that our model outperformed the nearest neighbor model. Specifically, the correlation coefficient, MSE, and RMSE for our model compared to the nearest neighbor model are 0.989 versus 0.982, 0.122 versus 0.192, and 0.349 versus 0.438, respectively (Figure 4g). This improvement is likely due to the more comprehensive physical interactions captured by the descriptor matrices and the superior fitting power of the deep learning models.

### 3.2 Prediction of DNA Strand Displacement Rate Constants

**Figure 5:**
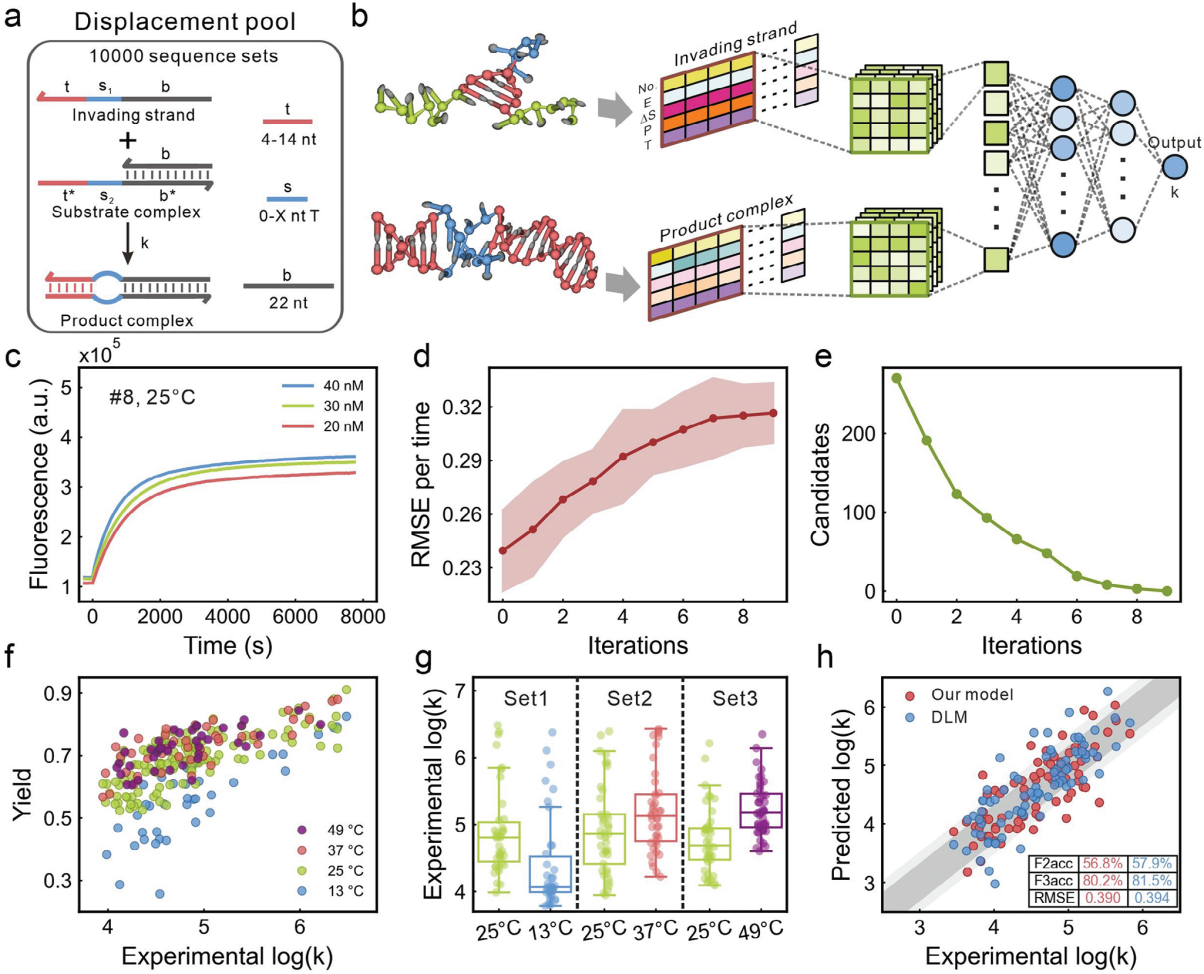
Results of model training and prediction of the DNA strand displacement rate constants. a) The strand displacement pool with 10 000 sequence sets. Invading strand consists of a toehold domain t, a spacer domain s1 and a branch migration domain b. The same letters with and without an asterisk are complementary domain. Spacer s1 and s2 are not complementary. b) The architecture of our model for predicting rate constants. c) Example fluorescence kinetics traces of a strand displacement reaction. d) The RMSE of models on the training set versus the number of iterations during active learning. e) The number of candidates versus the number of iterations during active learning. f) Rate constants versus yields of 240 DNA strand displacement reactions. g) Rate constants of DNA strand displacement reactions at different temperatures. There are 38, 41 and 41 reactions at 13 °C, 37 °C, and 49 °C, respectively. h) Prediction accuracy of our model and the DLM. The dark gray and light gray areas delineate metrics F2acc and F3acc, respectively.

To further challenge and highlight the generality of our model, we applied it to predict DNA strand displacement rate constants. To construct a strand displacement dataset, a strand displacement pool including 10 000 sequence sets was established (**Figure** **5**a). The length of the toehold domain was designed to range from 4 to 14 nucleotides. The was composed of 0 to X thymine nucleotides, where X was no greater than 10 and less than the length of the toehold domain. The branch migration domain was designed to encompass 22 nucleotides. Each sequence set included an invading strand, a substrate complex and a product complex. The invading strand aligns with the toehold domain of the substrate complex, triggering branch migration, which results in the formation of the product complex. The Nupack software was utilized to predict the secondary structure of each strand and the complexes, providing an insight into the most probable configurations. Subsequently, the Nupack‐calculated equilibrium unpaired probability of each nucleotide, serving as the fourth feature, was incorporated into the descriptor matrices. Moreover, recognizing that the rate constants of strand displacement reactions are temperature‐dependent, the fifth row was added to the descriptor matrices to represent the temperature feature with values normalized. For a strand displacement reaction, two descriptor matrices were fed into the model, each representing the invading strand and the product complex, respectively. The convolution kernel was 5 × 4, and other hyperparameters remained unchanged (Figure 5b). To acquire the labels for the samples within the strand displacement pool, fluorescence kinetic characterization analysis was executed, which leveraged the fluorescence spectrophotometer to measure the time kinetic traces of the reactions (Figure 5c). Subsequently, strand displacement rate constants were determined through a fitting analysis; see Section S3 (Supporting Information) for details.

Here the active learning was employed again to construct the strand displacement dataset. A total of 100 sequence sets were randomly selected from the strand displacement pool as the initial dataset (Figure S6, Supporting Information). We defined a threshold for maximal standard deviation at 0.5. During iteration of active learning, sequence sets with maximal standard deviation greater than the threshold were considered as candidates. In each iteration, up to 10 sequence sets were selected. During the iterative process of active learning, as the number of iterations increased, the mean RMSE of models on the training set gradually rose from the initial 0.239 to 0.317 (Figure 5d), while the number of candidates steadily decreased from the initial 270 to 0 (Figure 5e). A total of 81 sequence sets were selected during the active learning process (Figure S7, Supporting Information). Combined with the initial dataset, this resulted in a total of 181 sequence sets in the strand displacement dataset. A test set consisting of 48 sequence sets was established based on the aforementioned sequence design principle (Figure S8, Supporting Information).

We also investigated the influence of the temperature on DNA strand displacement rate constants. The strand displacement reactions of the above 229 sequence sets were initially conducted at 25 °C. We observed that the kinetic traces of reactions with smaller rate constants were more susceptible to interference from leaky reactions. Therefore, we selected 120 sequence sets with a mean yield greater than 0.5 within 9000 s and randomly assigned them to one of three different temperatures (13 °C, 37 °C, and 49 °C) for further analysis (Figure S9, Supporting Information). This resulted in a total of 240 strand displacement reactions. The best‐fit rate constants of these reactions across different temperatures spanned 2.5 orders of magnitude, with mean yields ranging from 0.26 to 0.92 (Figure 5f).

Further analysis of the rate constants for the 120 sequence sets at different temperatures revealed that the median values of log(k) at 13 °C, 37 °C, and 49 °C were 4.06, 5.13, and 5.18, respectively. At 25 °C, the median log(*k*) values for the same sequence sets were 4.81, 4.86, and 4.69, respectively (Figure 5g). The changes of rate constants under different temperatures relative to 25 °C were 15.6%, 5.6% and 10.4% respectively. These results indicate that higher temperatures generally lead to increased rate constants, while lower temperatures have a more pronounced effect on reducing rate constants.

A final strand displacement dataset was composed of 268 reactions across 181 sequence sets at varying temperatures (13 °C, 25 °C, 37 °C, and 49 °C), which 214 reactions were used as the training set and 54 reactions were used as the validation set. Additionally, 81 reactions from 48 sequence sets at different temperatures were used as the test set. For these sequences, the toehold domain encompassed 4 to 14 nucleotides, the spacer domain encompassed 0 to 10 thymine nucleotides. We trained both our model and the recurrent neural network‐based deep learning model (DLM) reported by Zhang et al.^[^ ^9b^ ^]^ Here, we employed two metrics defined by Zhang et al:^[^ ^9a^ ^]^ F2acc and F3acc, which respectively represent the proportions of predicted values with ratios less than 2 and 3 compared to the experimental values. Specifically, the F2acc, F3acc and RMSE for our model compared to DLM are 56.8% versus 57.9%, 80.2% versus 81.5%, and 0.39 versus 0.394, respectively (Figure 5h). The results suggest that the prediction performance of our model is comparable to that of the DLM.

## 4 Conclusion

In summary, we achieved accurate predictions of DNA hybridization free energies and strand displacement rate constants by combining quantum chemical calculations with deep learning model. To comprehensively describe molecular interaction information, we expanded the nearest neighbor model's 16 stacks to 400. Energy values were obtained through quantum chemical calculations, while entropy was derived using a physically motivated statistical model. DNA strands were processed into descriptor matrices and served as inputs for a convolutional neural network.

For the construction of hybridization dataset, 196 duplexes were selected from 20000 duplexes in DNA hybridization pools by using active learning. This approach significantly improved the efficiency of sample labeling. Notably, our model outperformed the nearest neighbor model in predicting hybridization free energies on the same test set. Moreover, our model can also predict rate constants of DNA strand displacement reactions. We constructed 10 000 sequence sets as strand displacement pool, from which 181 sequence sets were selected by active learning. A total of 268 reactions at different temperatures were investigated. The results show that our model performs on par with a recurrent neural network‐based deep learning model on the same test set.

Our model not only excels in accurately predicting hybridization free energies but also demonstrates superior predictive capabilities for rate constants in both basic and spacer‐containing strand displacement reactions. This underscores its superiority over the nearest neighbor model and the recurrent neural network‐based model.^[^ ^9b^ ^]^ With the rapid advancement of synthetic biology and DNA computing, we believe that our model will be pivotal in designing engineered oligonucleotides with desired thermodynamic and kinetic properties within DNA‐based systems, including PCR primers, sequencing probes, and components of DNA molecular networks. Accurate prediction of reaction parameters will minimize the need for extensive experimental optimization and ensure the expected function of nanodevices. Furthermore, the model will facilitate the design of numerous primers and probes, as well as the sequence construction of large‐scale DNA reaction networks.

## Conflict of Interest

The authors declare no conflict of interest.

## Supporting information



Supporting Information

## Data Availability

The data that support the findings of this study are available in the Supporting Information of this article.
