# Supplementary material for: Predicting DNA Reactions with a Quantum Chemistry‐Based Deep Learning Model
Source: Adv Sci (Weinh). 2024 Sep 19;11(42):2409880. doi: 10.1002/advs.202409880 (PMC11558088; doi:10.1002/advs.202409880)
Supplement: Supplementary file 1 — Supporting Information [file ADVS-11-2409880-s001.docx]

Supporting Information

Predicting DNA Reactions with a Quantum Chemistry-Based Deep Learning Model

Likun Wang^#^, Na Li^#^, Mengyao Cao, Yun Zhu, Xiewei Xiong, Li Li, Tong Zhu* and Hao Pei*

Table of contents

[1. Material and methods 2](#_Toc170029515)

[1.1 DNA oligonucleotides synthesis and preparation 2](#_Toc170029516)

[1.2 Thermodynamic fluorescence experiments 2](#_Toc170029517)

[1.3 Kinetic fluorescence experiments 2](#_Toc170029518)

[2. High resolution melting and free energy fitting 4](#_Toc170029519)

[2.1 Free energy fitting models 4](#_Toc170029520)

[2.2 Melt curves and fitting procedure 5](#_Toc170029521)

[3. Strand displacement and rate constant fitting 8](#_Toc170029522)

[3.1 Rate constant fitting models 8](#_Toc170029523)

[3.2 Normalized fluorescence data and best fit traces 9](#_Toc170029524)

[4. Additional experiments 13](#_Toc170029525)

[4.1 Contribution of different features to model performance 13](#_Toc170029526)

[References 55](#_Toc170029527)

# Material and methods

In this paper, we trained and validated our model on two datasets: free energies of DNA hybridization and rate constants of DNA strand displacement. We would like to introduce experimental methods for thermodynamic and kinetic fluorescence characterizations respectively. In addition, we would like to introduce the training method of the model.

## DNA oligonucleotides synthesis and preparation

All DNA oligonucleotides were ordered from Sangon Biotech. Oligonucleotides with a length greater than 10 nt were purified by high affinity purification, while those with a length less than 10 nt and modiﬁed oligonucleotides were puriﬁed by high performance liquid chromatography. Upon receipt, the lyophilized oligonucleotides were suspended in 1× TE buffer (10 mM Tris•HCl, pH 8.0, 1 mM EDTA), and stored at 4 ℃. The concentration of each oligonucleotide was quantitated by measuring the absorbance at 260 nm using Agilent Cary 60 spectrophotometer. All DNA duplexes in thermodynamic fluorescence experiments were prepared by annealing top and bottom strands in a 1:1 ratio. All DNA substrate complexes in kinetic fluorescence experiments were prepared by annealing a 1.2:1.2:1.1:1 ratio of ﬂuorophore labeled, quencher labeled, top and bottom strands. The buffer for all experiments and annealed complexes was 1× TE with 12.5 mM Mg^2+^, pH 8.0. All complexes were annealed in an ABI 2720 thermal cycler by heating to 95 ℃ for 5 min and then gradually cooling to 20 ℃ at a constant rate over a period of 2 h.

## Thermodynamic fluorescence experiments

Fluorescence versus temperature profiles of DNA duplexes were measured by a Bio-Rad CFX96 Touch Real-Time PCR. Each duplex was formulated at 6 different concentrations (1, 5, 10, 50, 100, 300 uM) in the buffer with 1X EvaGreen fluorescence dye, and each concentration was replicated 3 times to reduce experimental error. The solution was annealed and then subjected to high resolution melting. Melt curves of duplexes were monitored from 5 ℃ to 90 ℃, and fluorescence data were recorded every 0.2 ℃. Almost all oligonucleotides were in duplex states at low temperatures, EvaGreen specifically intercalated duplexes results in high fluorescence in the buffer. While at high temperatures almost all oligonucleotides were in single states, leading to low fluorescence in the buffer, which is partially caused by the temperature dependence of EvaGreen.

## Kinetic fluorescence experiments

Fluorescence versus time curves were performed in 1.4 mL quartz cuvettes by a Horiba Fluorologmax spectrofluorometer with a 4-sample changer. Kinetic fluorescence data were collected every 60 s with an external temperature water bath to control the temperature. Prior to experiments, all cuvettes were successively washed with deionized water 5 times, 70% ethanol 3 times, and deionized water 5 times. Substrate complexes were added to three cuvettes for each strand displacement to reach the final concentration of 10 nM. After the fluorescence stabilized, the cuvettes were removed from the instrument, and the one with the lowest fluorescence was placed in position 1, the one with the middle fluorescence was placed in position 2, and the one with the highest fluorescence was placed in position 3. After recording the fluorescence data 5 times, 40, 30, and 20 nM invading strands were added to each cuvette successively, with a mixing time of approximately 60 s. Kinetics fluorescence experiments for each strand displacement were measured between 4000 and 9000 seconds.

We measured strand displacement by observing fluorescence time courses using the X-probe architecture to minimize the number of unique chemically modified oligonucleotides. The initial fluorescence of the substrate complex was low, owing to the co-localization of the fluorophore and quencher, however, upon the addition of the invading strand, an increase in fluorescence was observed. To normalize the experimental fluorescence data into instantaneous reaction yields, we measured negative and positive control values. The negative control value equaled the mean ﬂuorescence of substrate complexes in the first 5 times, corresponding to 0% yield. The positive control value equaled the increments of fluorescence when adding 10 nM of unquenched fluorophore strand to the buffer, corresponding to 100% yield. The instantaneous yield of each experimental data point was calculated by$(F_{n}-F_{\text{Negative}})/F_{\text{Positive}}$. All experimental fluorescence data were normalized and recorded as *Y*(*t*).

# High resolution melting and free energy fitting

Here, we describe a high resolution melting (HRM) method for measuring hybridization free energies of DNA duplexes.

## Free energy fitting models

There are two methods to obtain free energies from fluorescence versus temperature curves: (1) A median line is determined by the upper and lower baselines of each melt curve, where melting temperature$T_{m}$is the temperature at which the median line and the melt curve intersect. Because,

$$\Delta G=\Delta H-T\Delta S$$

$$\Delta G=-RTln\left( K \right)$$

Thus we have,

$$\frac{1}{T}=\frac{R}{\Delta H}ln(K)+\frac{\Delta S}{\Delta H}$$

Each melt curve is transformed to the reciprocal temperature versus the natural logarithm of the equilibrium constant.$\Delta H$and$\Delta S$can be obtained by averaging values from fits of individual curves.

(2) Taking the negative first-order derivative of the fluorescence to the temperature for each melt curve, and the temperature corresponding to the maximum is considered to be melting temperature$T_{m}$. For a pair of complementary oligonucleotides A and B:

$$AB\rightleftharpoons A+B K=\frac{[A][B]}{[AB]}$$

At$T_{m}$,

$$\left[ AB \right]=\left[ A \right]=\left[ B \right]=\frac{C_{0}}{4}$$

Where$C_{0}$is the initial concentration of oligonucleotide A. Thus we have,

$$\frac{1}{T_{m}}=\frac{R}{\Delta H}\ln\left( \frac{C_{0}}{4} \right)+\frac{\Delta S}{\Delta H}$$

$\Delta H$and$\Delta S$are fitted by the equation between concentrations and the reciprocal of mean melting temperature.$\Delta H$and$\Delta S$are typically assumed to be temperature invariant, so$\Delta G$can be calculated by$\Delta G=\Delta H-T\Delta S.$

However, the first method has proven to be unreliable, as it is indispensable to determine upper and lower baselines for each melt curve. Choices of baselines are subjective in most cases and slight changes in baselines cause significant deviations. As a result, we adopt the second method. Unless otherwise specified, the free energy mentioned in the text refer to those at 37°C.

## Melt curves and fitting procedure

To obtain experimental hybridization free energies, we measured melt curves of duplexes hybridization at different concentrations (2 μM, 10 μM, 20 μM, 100 μM, 200 μM, 600 μM) using real-time fluorescent quantitative PCR. By solving the derivative of the fluorescence intensity with respect to temperatures, we obtained melting temperatures. We derived ΔH and ΔS of the hybridization reaction by fitting the functional relationship between melting temperatures and concentrations. Finally, we calculated DNA hybridization free energies at 37°C using the Gibbs free energy equation.

Melt curves and fitting procedure for 244 duplexes are presented in Figure S2 to Figure S4, with an example illustrated in Figure S1. 64 initial duplexes are shown in Figure S2. 132 duplexes selected by active learning are shown in Figure S3. 48 duplexes as the test set are shown in Figure S4. Sequence sets and thermodynamic parameters of 244 DNA duplexes are shown in Table S5.


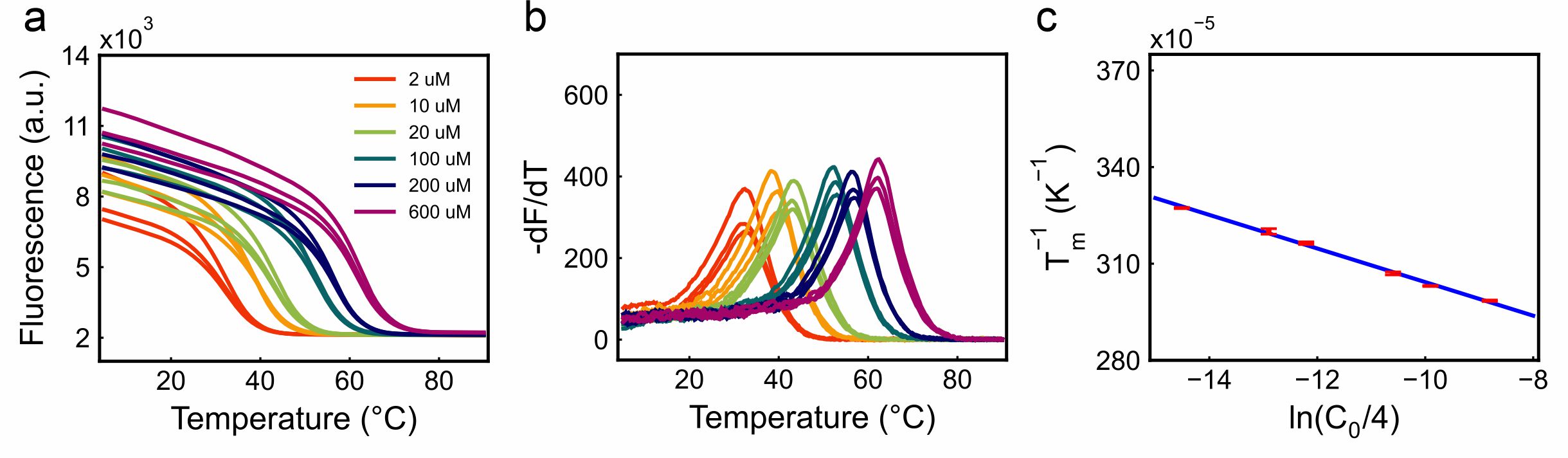


Figure S1. Procedure for measuring hybridization free energies of a DNA duplex. (a) Melt curves of different concentrations of #1 duplex. (b) Negative first-order derivative of the fluorescence to the temperature for each melt curves are calculated. (c) Hybridization thermodynamic parameters of #1 duplex are fitted by the equation between concentrations and the reciprocal of T_m_.


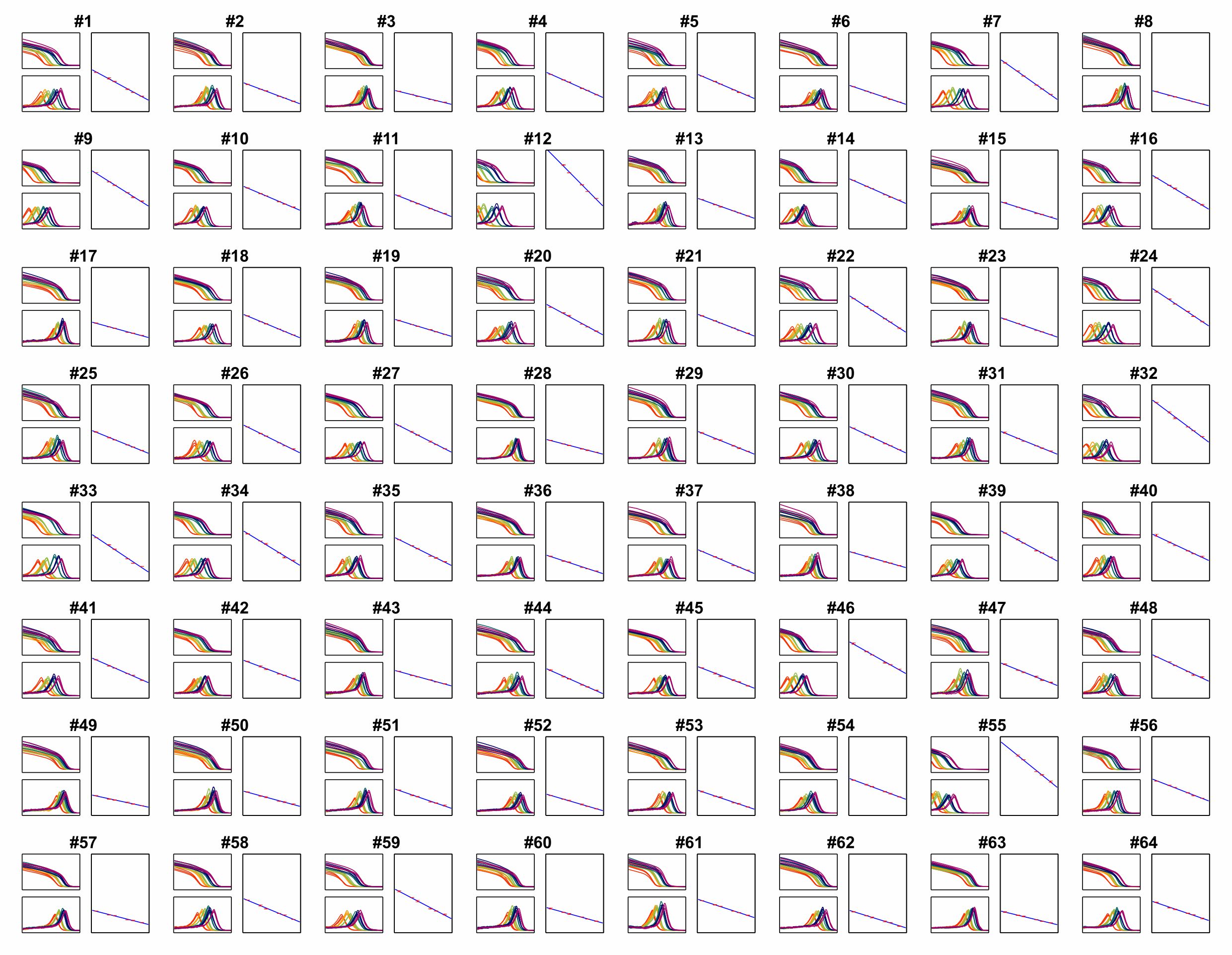


Figure S2. Melt curves and fitting procedure of 64 initial duplexes.


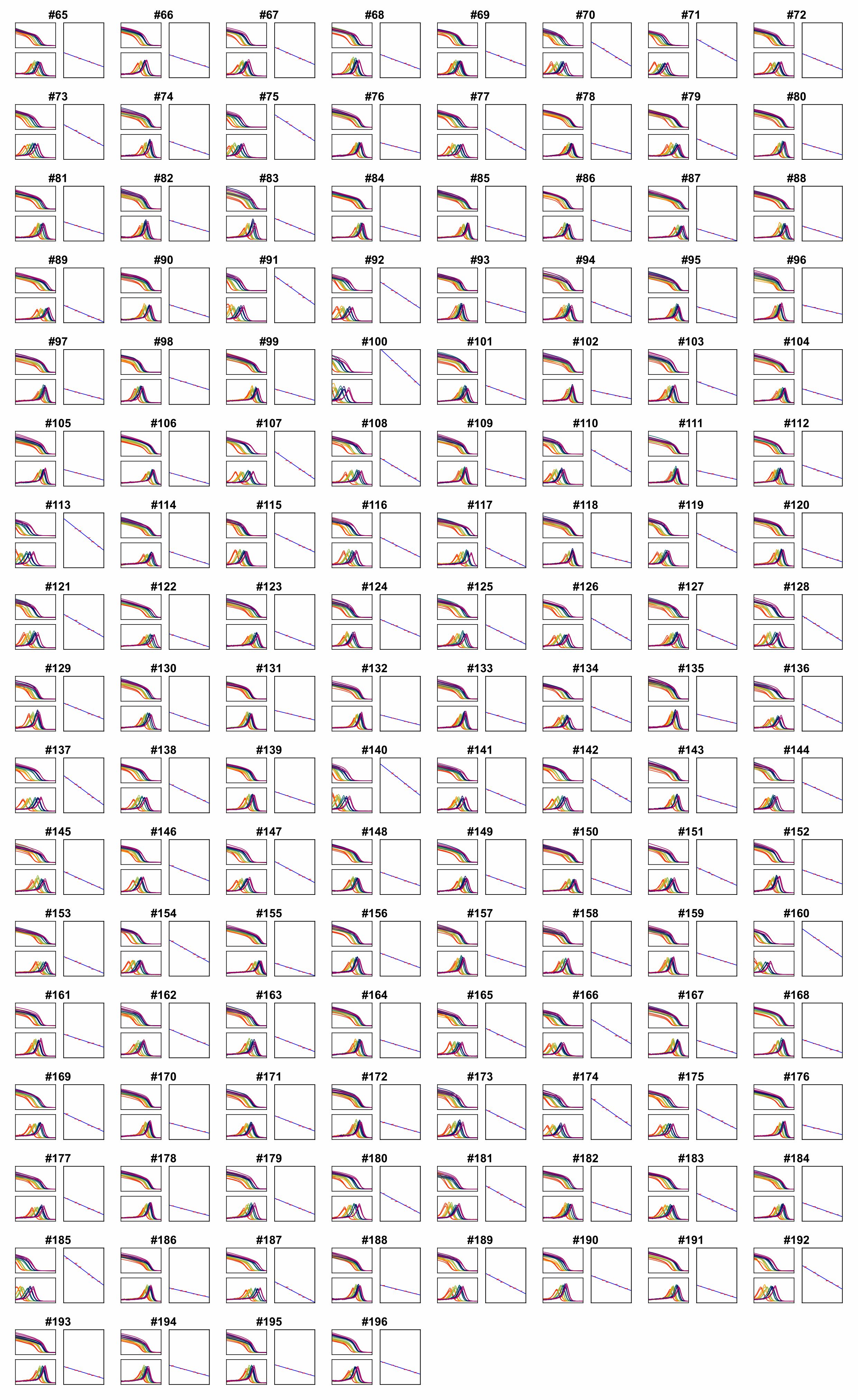


Figure S3. Melt curves and fitting procedure of 132 duplexes selected by active learning.


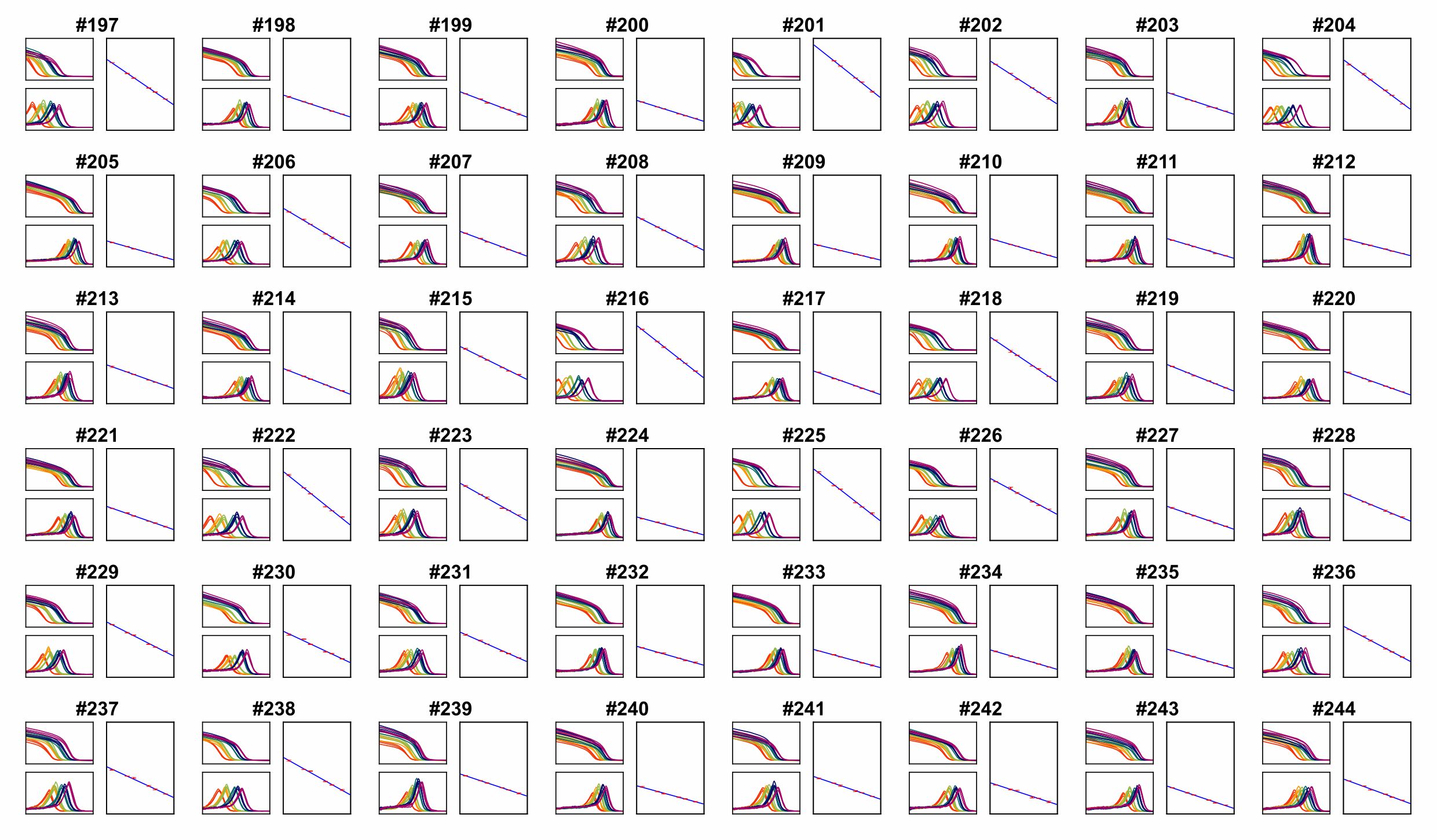


Figure S4. Melt curves and fitting procedure of 48 duplexes as the test set.

# Strand displacement and rate constant fitting

Here, we describe a fluorescence versus time curves analysis method for measuring rate constants of DNA strand displacement.

## Rate constant fitting models

Generally, DNA strand displacement is considered a second-order reaction, with its rate depends on the concentration of the invading strand when the concentration of the invading strand is higher than the substrate complex. While Genot et al. discovered the reaction rate is insensitive to the concentration of the invading strand with 14 nt toehold domains and 17 nt spacer domains, the overall displacement kinetic is approximately first-order.^[^[^1^](#_ENREF_1)^]^ Besides that, Machinek et al. found the first-order rate constant fitting model is much better for strand displacement involving 10 nt toehold domains and proximal or central mismatch within the branch migration domain.^[^[^2^](#_ENREF_2)^]^

Therefore, we considered two rate constant fitting models. These models assume that some of substrate complexes do not participate in the complete strand displacement. Each model contains two parameters, rate constant *k* and yield *y*. First-order rate constant fitting model as follows:

$$A+BC_{\text{good}}\to ABC$$

$$ABC\overset{k_{1}}{\to}AC+B$$

$$\frac{[BC_{\text{good}}]_{0}}{[BC]_{0}}=\text{Yield}$$

The concentration of invading strand A is higher than substrate complex BC and toehold binding is sufficiently rapid, so the concentration of complex stand ABC is approximated to$BC_{\text{good}}$.

Second-order rate constant fitting model as follows:

$$A+BC_{\text{good}}\overset{k_{2}}{\to}AC+B$$

$$\frac{[BC_{\text{good}}]_{0}}{[BC]_{0}}=\text{Yield}$$

Each strand displacement was performed three times with different concentrations of invading strands. We assumed that they have the same rate constant and different yields, so each rate constant fitting model has four parameters: rate constant *k*, yield *y_1_* in the first experiment with 20 nM invading strand, yield *y_2_* in the second experiment with 30 nM invading strand and yield *y_3_* in the third experiment with 40 nM invading strand*.* Parameters of models were fitted using least_squares method in lmfit library (https://lmfit.github.io/lmfit-py) to minimize the root mean squared error (RMSE) between experimental data *Y*(*t*) and simulated data *F*(*t, k, y_1_, y_2_, y_3_*), where simulated data was calculated by odeint function in scipy library (https://scipy.org) at time *t* with four parameters. The initial value of *k* (log) was randomly selected from a range of 1 to 9, *y_1_* was randomly selected from a range of 0 to 1, *y_2_* was set equal to *y_1_* plus a variable number between 0 and 1, *y_3_* was set equal to *y_2_* plus a variable number between 0 and 1, and values of *y_1_*, *y_2_* and *y_3_* were limited to the maximum in each normalized experimental data and 1. Different results were obtained by fitting different initial values, and the result with the smallest RMSE was selected as optimal parameters for each model. Finally, two sets of parameters were obtained for each reaction: *k_1_*, *y_1__k_1_*, *y_2__k_1_*, *y_3__k_1_* and *k_2_*, *y_1__k_2_*, *y_2__k_2_*, *y_3__k_2_*.

Before active learning, we found that 79 of 100 initial reactions are closer to first-order because their RMSE values are smaller. The premise for using the first-order rate constant fitting model is that the binding of the toehold is rapid and irreversible, and the displacement rate of the branch migration domain is slow. However, a subset of the 79 reactions have shorter toehold domains, rendering the approximation of overall displacement kinetics as first-order unreasonable. Moreover, a comparison of the yields obtained from the first-order and second-order rate constant fitting models revealed that the large standard deviations in the yields obtained from the first-order rate constant fitting model made it untenable. Despite the fact that the first-order rate constant fitting model may prove advantageous for certain reactions, the second-order rate constant fitting model provides more reasonable and useful parameterization. As such, we decided to use second-order rate constants as the labels.

## Normalized fluorescence data and best fit traces

Normalized fluorescence data and best fit traces for 349 strand displacement across 229 sequence sets are presented in Figure S6 to Figure S9, with an example illustrated in Figure S5. Reactions of 100 initial sequence sets are shown in Figure S6. Reactions of 81 sequence sets selected by active learning are shown in Figure S7. Reactions of 48 sequence sets as the test set are shown in Figure S8. Reactions of 120 sequence sets at different temperatures are shown in Figure S9. The red dotted line represents first-order rate constant fitting model, while the blue dotted line represents second-order rate constant fitting model. Sequence sets, kinetic parameters and experiment temperatures of 349 DNA strand displacement are shown in Table S6 and Table S7.


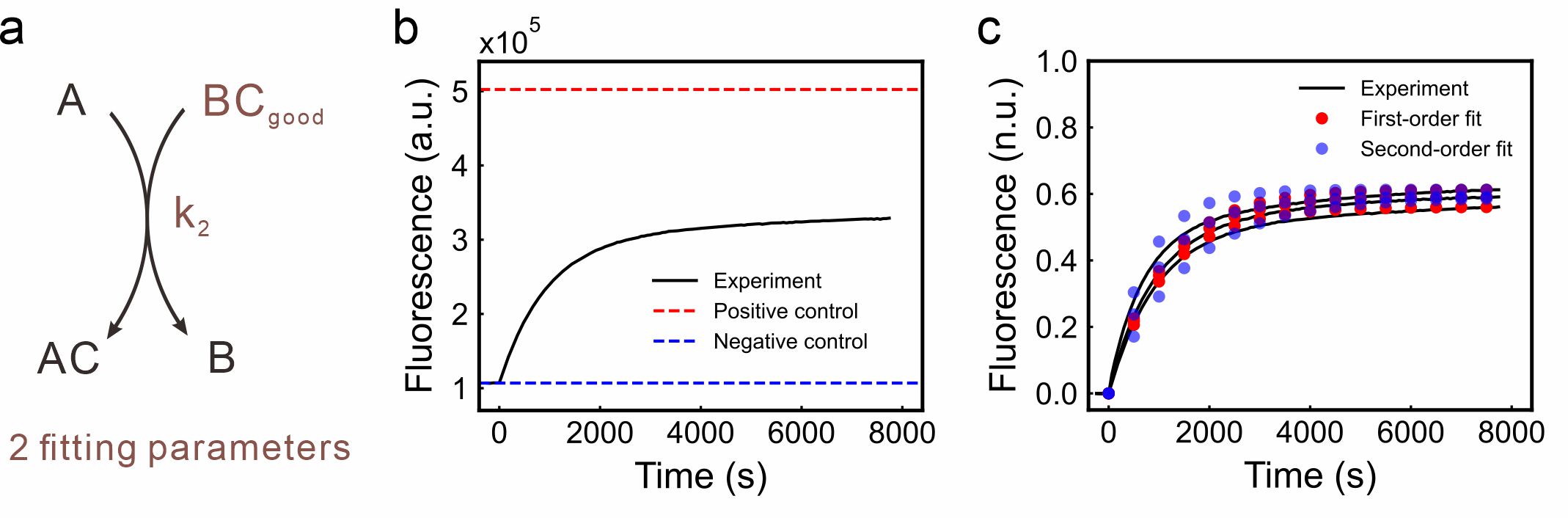


Figure S5. Procedure for measuring the rate constant of a DNA strand displacement. (a) Schematic of the second-order rate constant fitting model. (b) Determining instantaneous yield of #8 reaction using positive and negative controls. (c) Normalized fluorescence data and best fit traces for #8 reaction.


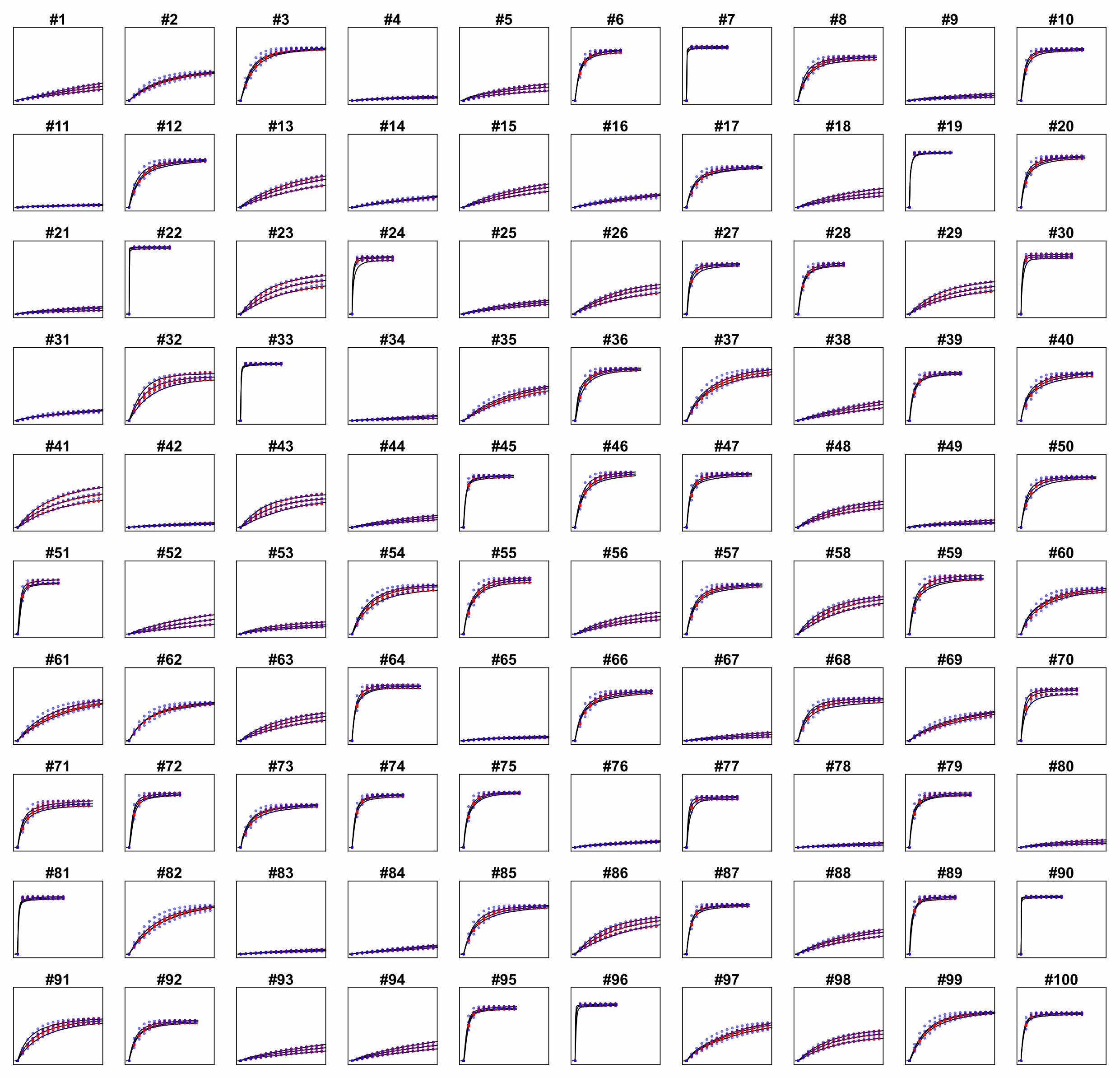


Figure S6. Normalized fluorescence data and best fit traces for strand displacement of 100 initial sequence sets.


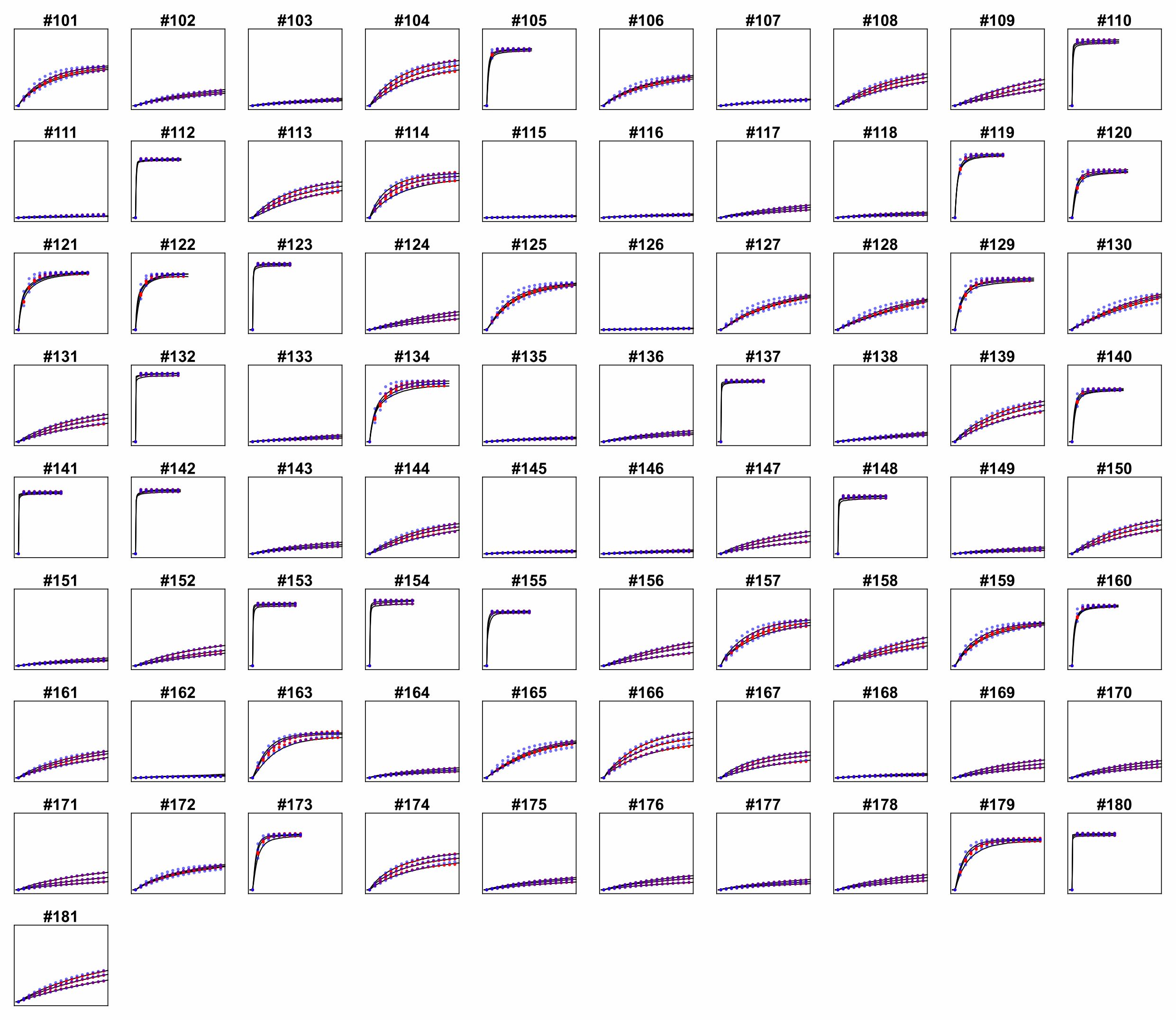


Figure S7. Normalized fluorescence data and best fit traces for strand displacement of 81 sequence sets selected by active learning.


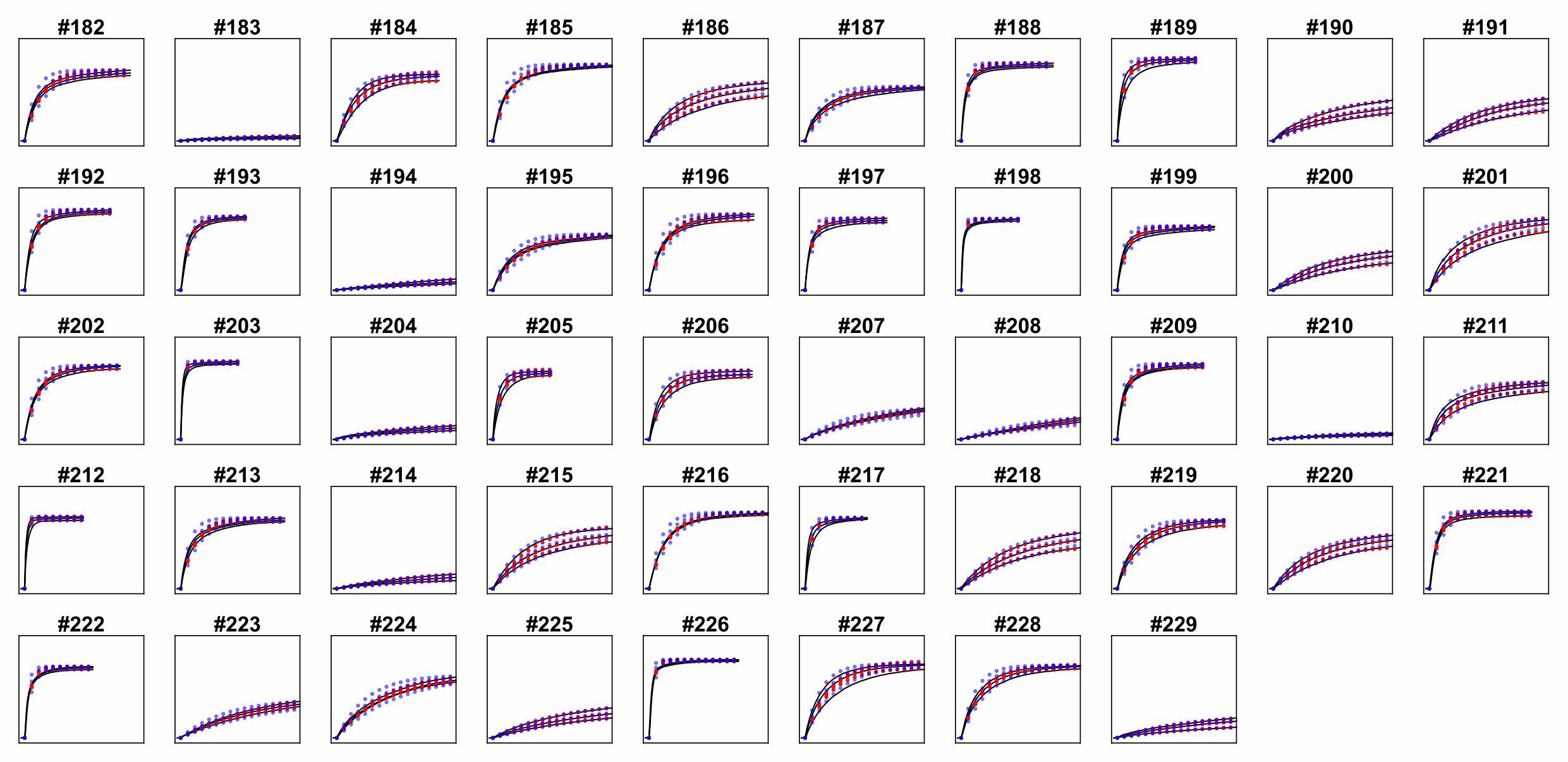


Figure S8. Normalized fluorescence data and best fit traces for strand displacement of 48 sequence sets as the test set.


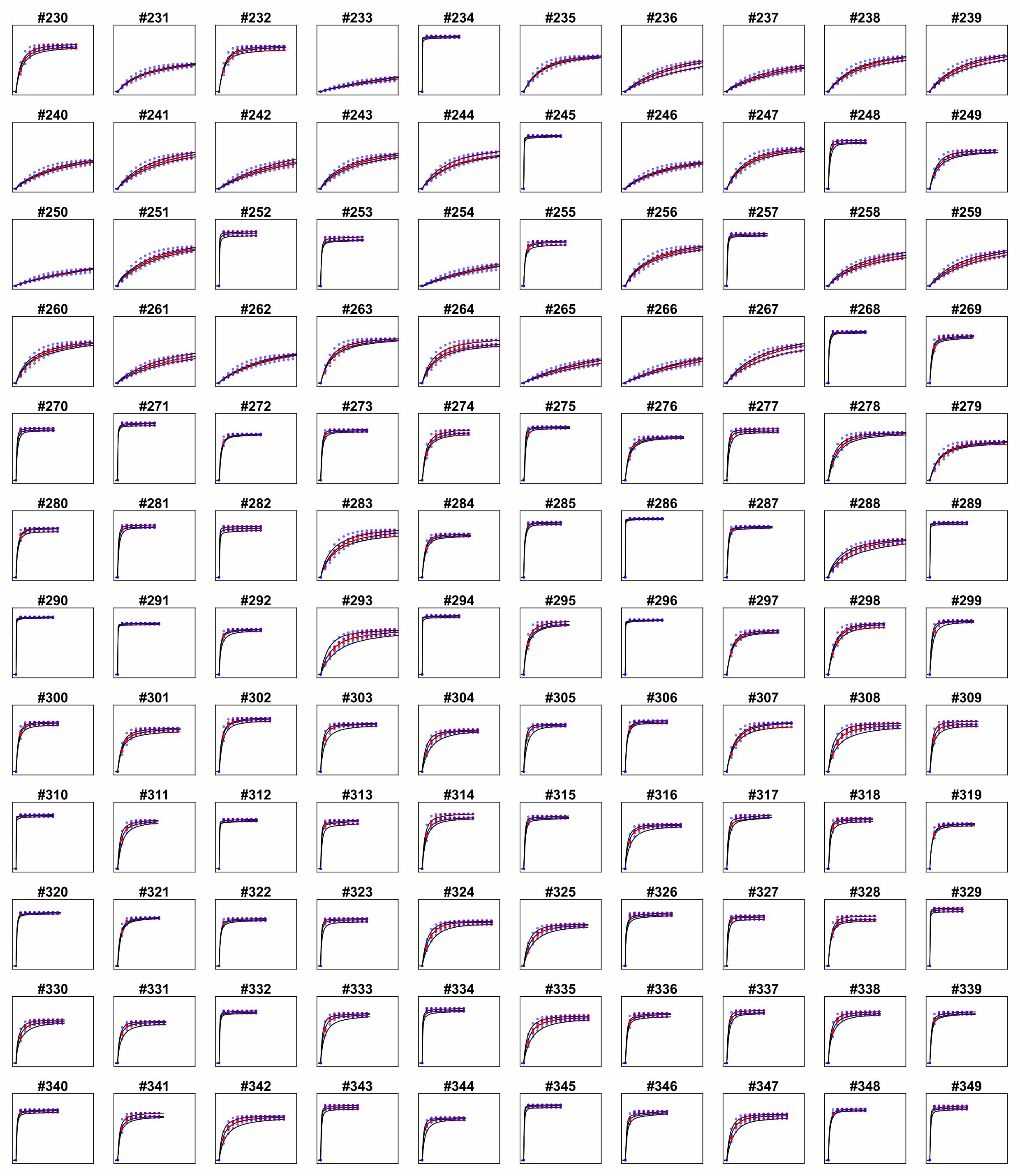


Figure S9. Normalized fluorescence data and best-fit traces for strand displacement of 120 sequence sets at different temperatures.

# Additional experiments

## Contribution of different features to model performance

To assess the model's ability to capture key interaction parameters, we tested its performance with certain features replaced or removed (Figure S10).

After replacing the energy calculated using quantum chemistry with the energy of the nearest neighbor model, the model's RMSE on the test set increased. This highlights the effectiveness of expanding the 16 stacks in the nearest neighbor model to 400. The “Number” had a negligible impact on prediction accuracy, with RMSE variations on test set remaining below 1%. This is because the number is essentially arbitrary and has little relation to molecular properties. After removing the feature E_QC_, RMSE significantly increased, underscoring it is a key interaction parameter. The “Entropy” also impacted prediction accuracy, particularly in predicting DNA hybridization free energies, suggesting it captures critical information about dangles. For predicting DNA strand displacement rate constants, “Energy” and unpaired probability of bases emerge as key features, which the former describes the physical interactions between base pairs and the latter provides essential insights into the stability of DNA secondary structures.


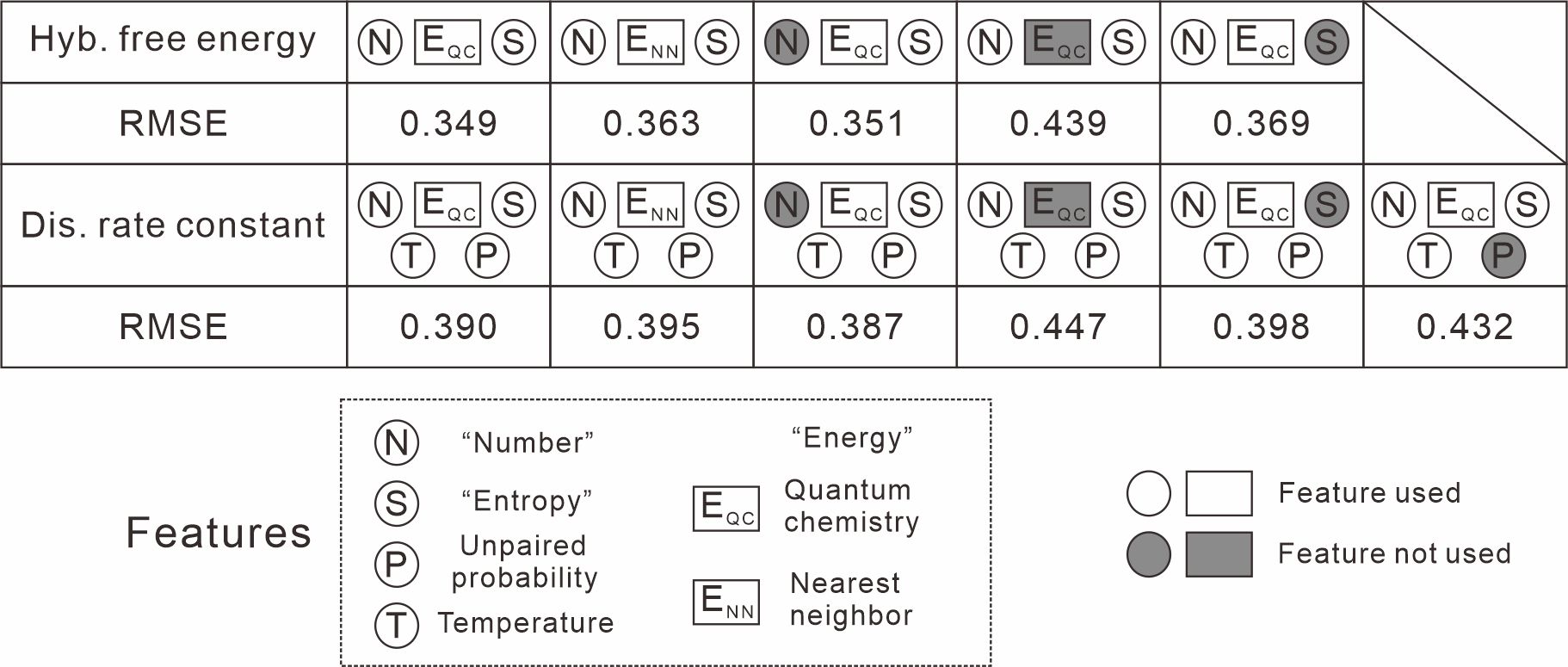


Figure S10. Evaluating the prediction accuracy with different features in the descriptor matrices.

**Table S1.** “Number” for all types of terms. Unless otherwise specified, sequences in the article are presented in the direction of 5' to 3'.

|  | number |  | number |  | number |  | number |
| --- | --- | --- | --- | --- | --- | --- | --- |
| AAAA | 1.01 | AATA | 2.01 | AACA | 3.01 | AAGA | 4.01 |
| AAAT | 1.02 | AATT | 2.02 | AACT | 3.02 | AAGT | 4.02 |
| AAAC | 1.03 | AATC | 2.03 | AACC | 3.03 | AAGC | 4.03 |
| AAAG | 1.04 | AATG | 2.04 | AACG | 3.04 | AAGG | 4.04 |
| AAA) | 1.05 | AAT) | 2.05 | AAC) | 3.05 | AAG) | 4.05 |
| TAAA | 1.06 | TATA | 2.06 | TACA | 3.06 | TAGA | 4.06 |
| TAAT | 1.07 | TATT | 2.07 | TACT | 3.07 | TAGT | 4.07 |
| TAAC | 1.08 | TATC | 2.08 | TACC | 3.08 | TAGC | 4.08 |
| TAAG | 1.09 | TATG | 2.09 | TACG | 3.09 | TAGG | 4.09 |
| TAA) | 1.1 | TAT) | 2.1 | TAC) | 3.1 | TAG) | 4.1 |
| CAAA | 1.11 | CATA | 2.11 | CACA | 3.11 | CAGA | 4.11 |
| CAAT | 1.12 | CATT | 2.12 | CACT | 3.12 | CAGT | 4.12 |
| CAAC | 1.13 | CATC | 2.13 | CACC | 3.13 | CAGC | 4.13 |
| CAAG | 1.14 | CATG | 2.14 | CACG | 3.14 | CAGG | 4.14 |
| CAA) | 1.15 | CAT) | 2.15 | CAC) | 3.15 | CAG) | 4.15 |
| GAAA | 1.16 | GATA | 2.16 | GACA | 3.16 | GAGA | 4.16 |
| GAAT | 1.17 | GATT | 2.17 | GACT | 3.17 | GAGT | 4.17 |
| GAAC | 1.18 | GATC | 2.18 | GACC | 3.18 | GAGC | 4.18 |
| GAAG | 1.19 | GATG | 2.19 | GACG | 3.19 | GAGG | 4.19 |
| GAA) | 1.2 | GAT) | 2.2 | GAC) | 3.2 | GAG) | 4.2 |
| (AAA | 1.21 | (ATA | 2.21 | (ACA | 3.21 | (AGA | 4.21 |
| (AAT | 1.22 | (ATT | 2.22 | (ACT | 3.22 | (AGT | 4.22 |
| (AAC | 1.23 | (ATC | 2.23 | (ACC | 3.23 | (AGC | 4.23 |
| (AAG | 1.24 | (ATG | 2.24 | (ACG | 3.24 | (AGG | 4.24 |
| (AA) | 1.25 | (AT) | 2.25 | (AC) | 3.25 | (AG) | 4.25 |
| ATAA | 5.01 | ATTA | 6.01 | ATCA | 7.01 | ATGA | 8.01 |
| ATAT | 5.02 | ATTT | 6.02 | ATCT | 7.02 | ATGT | 8.02 |
| ATAC | 5.03 | ATTC | 6.03 | ATCC | 7.03 | ATGC | 8.03 |
| ATAG | 5.04 | ATTG | 6.04 | ATCG | 7.04 | ATGG | 8.04 |
| ATA) | 5.05 | ATT) | 6.05 | ATC) | 7.05 | ATG) | 8.05 |
| TTAA | 5.06 | TTTA | 6.06 | TTCA | 7.06 | TTGA | 8.06 |
| TTAT | 5.07 | TTTT | 6.07 | TTCT | 7.07 | TTGT | 8.07 |
| TTAC | 5.08 | TTTC | 6.08 | TTCC | 7.08 | TTGC | 8.08 |
| TTAG | 5.09 | TTTG | 6.09 | TTCG | 7.09 | TTGG | 8.09 |
| TTA) | 5.1 | TTT) | 6.1 | TTC) | 7.1 | TTG) | 8.1 |
| CTAA | 5.11 | CTTA | 6.11 | CTCA | 7.11 | CTGA | 8.11 |
| CTAT | 5.12 | CTTT | 6.12 | CTCT | 7.12 | CTGT | 8.12 |
| CTAC | 5.13 | CTTC | 6.13 | CTCC | 7.13 | CTGC | 8.13 |
| CTAG | 5.14 | CTTG | 6.14 | CTCG | 7.14 | CTGG | 8.14 |
| CTA) | 5.15 | CTT) | 6.15 | CTC) | 7.15 | CTG) | 8.15 |
| GTAA | 5.16 | GTTA | 6.16 | GTCA | 7.16 | GTGA | 8.16 |
| GTAT | 5.17 | GTTT | 6.17 | GTCT | 7.17 | GTGT | 8.17 |
| GTAC | 5.18 | GTTC | 6.18 | GTCC | 7.18 | GTGC | 8.18 |
| GTAG | 5.19 | GTTG | 6.19 | GTCG | 7.19 | GTGG | 8.19 |
| GTA) | 5.2 | GTT) | 6.2 | GTC) | 7.2 | GTG) | 8.2 |
| (TAA | 5.21 | (TTA | 6.21 | (TCA | 7.21 | (TGA | 8.21 |
| (TAT | 5.22 | (TTT | 6.22 | (TCT | 7.22 | (TGT | 8.22 |
| (TAC | 5.23 | (TTC | 6.23 | (TCC | 7.23 | (TGC | 8.23 |
| (TAG | 5.24 | (TTG | 6.24 | (TCG | 7.24 | (TGG | 8.24 |
| (TA) | 5.25 | (TT) | 6.25 | (TC) | 7.25 | (TG) | 8.25 |
| ACAA | 9.01 | ACTA | 10.01 | ACCA | 11.01 | ACGA | 12.01 |
| ACAT | 9.02 | ACTT | 10.02 | ACCT | 11.02 | ACGT | 12.02 |
| ACAC | 9.03 | ACTC | 10.03 | ACCC | 11.03 | ACGC | 12.03 |
| ACAG | 9.04 | ACTG | 10.04 | ACCG | 11.04 | ACGG | 12.04 |
| ACA) | 9.05 | ACT) | 10.05 | ACC) | 11.05 | ACG) | 12.05 |
| TCAA | 9.06 | TCTA | 10.06 | TCCA | 11.06 | TCGA | 12.06 |
| TCAT | 9.07 | TCTT | 10.07 | TCCT | 11.07 | TCGT | 12.07 |
| TCAC | 9.08 | TCTC | 10.08 | TCCC | 11.08 | TCGC | 12.08 |
| TCAG | 9.09 | TCTG | 10.09 | TCCG | 11.09 | TCGG | 12.09 |
| TCA) | 9.1 | TCT) | 10.1 | TCC) | 11.1 | TCG) | 12.1 |
| CCAA | 9.11 | CCTA | 10.11 | CCCA | 11.11 | CCGA | 12.11 |
| CCAT | 9.12 | CCTT | 10.12 | CCCT | 11.12 | CCGT | 12.12 |
| CCAC | 9.13 | CCTC | 10.13 | CCCC | 11.13 | CCGC | 12.13 |
| CCAG | 9.14 | CCTG | 10.14 | CCCG | 11.14 | CCGG | 12.14 |
| CCA) | 9.15 | CCT) | 10.15 | CCC) | 11.15 | CCG) | 12.15 |
| GCAA | 9.16 | GCTA | 10.16 | GCCA | 11.16 | GCGA | 12.16 |
| GCAT | 9.17 | GCTT | 10.17 | GCCT | 11.17 | GCGT | 12.17 |
| GCAC | 9.18 | GCTC | 10.18 | GCCC | 11.18 | GCGC | 12.18 |
| GCAG | 9.19 | GCTG | 10.19 | GCCG | 11.19 | GCGG | 12.19 |
| GCA) | 9.2 | GCT) | 10.2 | GCC) | 11.2 | GCG) | 12.2 |
| (CAA | 9.21 | (CTA | 10.21 | (CCA | 11.21 | (CGA | 12.21 |
| (CAT | 9.22 | (CTT | 10.22 | (CCT | 11.22 | (CGT | 12.22 |
| (CAC | 9.23 | (CTC | 10.23 | (CCC | 11.23 | (CGC | 12.23 |
| (CAG | 9.24 | (CTG | 10.24 | (CCG | 11.24 | (CGG | 12.24 |
| (CA) | 9.25 | (CT) | 10.25 | (CC) | 11.25 | (CG) | 12.25 |
| AGAA | 13.01 | AGTA | 14.01 | AGCA | 15.01 | AGGA | 16.01 |
| AGAT | 13.02 | AGTT | 14.02 | AGCT | 15.02 | AGGT | 16.02 |
| AGAC | 13.03 | AGTC | 14.03 | AGCC | 15.03 | AGGC | 16.03 |
| AGAG | 13.04 | AGTG | 14.04 | AGCG | 15.04 | AGGG | 16.04 |
| AGA) | 13.05 | AGT) | 14.05 | AGC) | 15.05 | AGG) | 16.05 |
| TGAA | 13.06 | TGTA | 14.06 | TGCA | 15.06 | TGGA | 16.06 |
| TGAT | 13.07 | TGTT | 14.07 | TGCT | 15.07 | TGGT | 16.07 |
| TGAC | 13.08 | TGTC | 14.08 | TGCC | 15.08 | TGGC | 16.08 |
| TGAG | 13.09 | TGTG | 14.09 | TGCG | 15.09 | TGGG | 16.09 |
| TGA) | 13.1 | TGT) | 14.1 | TGC) | 15.1 | TGG) | 16.1 |
| CGAA | 13.11 | CGTA | 14.11 | CGCA | 15.11 | CGGA | 16.11 |
| CGAT | 13.12 | CGTT | 14.12 | CGCT | 15.12 | CGGT | 16.12 |
| CGAC | 13.13 | CGTC | 14.13 | CGCC | 15.13 | CGGC | 16.13 |
| CGAG | 13.14 | CGTG | 14.14 | CGCG | 15.14 | CGGG | 16.14 |
| CGA) | 13.15 | CGT) | 14.15 | CGC) | 15.15 | CGG) | 16.15 |
| GGAA | 13.16 | GGTA | 14.16 | GGCA | 15.16 | GGGA | 16.16 |
| GGAT | 13.17 | GGTT | 14.17 | GGCT | 15.17 | GGGT | 16.17 |
| GGAC | 13.18 | GGTC | 14.18 | GGCC | 15.18 | GGGC | 16.18 |
| GGAG | 13.19 | GGTG | 14.19 | GGCG | 15.19 | GGGG | 16.19 |
| GGA) | 13.2 | GGT) | 14.2 | GGC) | 15.2 | GGG) | 16.2 |
| (GAA | 13.21 | (GTA | 14.21 | (GCA | 15.21 | (GGA | 16.21 |
| (GAT | 13.22 | (GTT | 14.22 | (GCT | 15.22 | (GGT | 16.22 |
| (GAC | 13.23 | (GTC | 14.23 | (GCC | 15.23 | (GGC | 16.23 |
| (GAG | 13.24 | (GTG | 14.24 | (GCG | 15.24 | (GGG | 16.24 |
| (GA) | 13.25 | (GT) | 14.25 | (GC) | 15.25 | (GG) | 16.25 |
| A)A | 17.1 | A)C | 19.1 | A(A | 21.1 | A(C | 23.1 |
| T)A | 17.2 | T)C | 19.2 | T(A | 21.2 | T(C | 23.2 |
| C)A | 17.3 | C)C | 19.3 | C(A | 21.3 | C(C | 23.3 |
| G)A | 17.4 | G)C | 19.4 | G(A | 21.4 | G(C | 23.4 |
| A)T | 18.1 | A)G | 20.1 | A(T | 22.1 | A(G | 24.1 |
| T)T | 18.2 | T)G | 20.2 | T(T | 22.2 | T(G | 24.2 |
| C)T | 18.3 | C)G | 20.3 | C(T | 22.3 | C(G | 24.3 |
| G)T | 18.4 | G)G | 20.4 | G(T | 22.4 | G(G | 24.4 |
| *A | 25 | A | 29 | A) | 33 | (A | 37 |
| *T | 26 | T | 30 | T) | 34 | (T | 38 |
| *C | 27 | C | 31 | C) | 35 | (C | 39 |
| *G | 28 | G | 32 | G) | 36 | (G | 40 |
| . | 41 |  |  |  |  |  |  |

**Table S2.** Interaction energies of 200 stacking terms. All values are reported in kcal/mol.

|  | a_hb1 | a_hb2 | a_sk1 | a_sk2 | a_sk3 | a_sk4 | b_hb1 | b_hb2 | b_sk1 | b_sk2 | b_sk3 | b_sk4 |
| --- | --- | --- | --- | --- | --- | --- | --- | --- | --- | --- | --- | --- |
| AAAA | -8.46 | -8.49 | -5.01 | -4.73 | -0.70 | -2.67 | -8.49 | -10.08 | -4.53 | -5.28 | -1.30 | -1.80 |
| AAAT | -9.06 | -3.73 | -5.21 | -5.75 | -0.73 | -1.19 | -9.83 | -8.57 | -5.51 | -4.93 | -0.50 | -1.15 |
| AAAC | -8.89 | -7.56 | -4.16 | -4.41 | -0.44 | -1.72 | -7.62 | -9.03 | -3.51 | -4.85 | -1.15 | -0.69 |
| AAAG | -10.33 | -10.93 | -5.50 | -4.95 | -0.68 | -1.95 | -4.10 | -7.70 | -5.83 | -4.44 | -0.92 | -1.56 |
| TAAA | -11.33 | -11.21 | -5.29 | -3.86 | -0.71 | -1.76 | -8.99 | -8.23 | -5.98 | -3.81 | -0.18 | -2.96 |
| TAAT | -8.83 | -7.43 | -5.06 | -5.31 | -0.96 | -1.07 | -9.67 | -7.58 | -5.74 | -4.68 | -0.42 | -0.40 |
| TAAC | -10.17 | -9.73 | -4.50 | -4.34 | -0.67 | -1.72 | -8.47 | -7.01 | -6.60 | -4.38 | -0.42 | -1.54 |
| TAAG | -10.52 | -10.77 | -4.90 | -4.92 | -0.52 | -1.51 | -9.93 | -9.09 | -5.33 | -4.66 | -1.15 | -1.47 |
| CAAA | -7.28 | -7.47 | -5.00 | -4.60 | -0.36 | -1.80 | -9.21 | -7.54 | -5.46 | -4.55 | -0.72 | -1.57 |
| CAAT | -9.89 | -10.79 | -6.10 | -3.26 | -1.01 | -1.69 | -9.17 | -9.72 | -3.76 | -4.53 | -0.92 | -0.46 |
| CAAC | -8.52 | -9.86 | -5.04 | -3.87 | -0.76 | -0.96 | -5.85 | -9.07 | -5.07 | -5.19 | -0.48 | -1.28 |
| CAAG | -4.64 | -8.75 | -4.23 | -4.86 | -0.80 | -2.06 | -8.14 | -9.61 | -4.86 | -4.90 | -0.41 | -2.10 |
| GAAA | -10.05 | -9.11 | -5.40 | -3.74 | -1.27 | -1.36 | -9.23 | -6.63 | -5.35 | -4.29 | -0.56 | -1.83 |
| GAAT | -5.81 | -7.72 | -5.01 | -4.47 | -0.76 | -1.38 | -6.68 | -8.76 | -5.31 | -4.72 | -1.57 | -1.75 |
| GAAC | -10.59 | -9.99 | -5.40 | -4.09 | -1.03 | -1.12 | -9.25 | -8.22 | -3.47 | -4.78 | -0.46 | -2.06 |
| GAAG | -10.16 | -10.34 | -5.29 | -4.13 | -1.20 | -0.87 | -8.39 | -6.74 | -5.62 | -4.83 | -1.00 | -0.99 |
| AATA | -11.02 | -8.31 | -5.34 | -5.81 | -1.04 | 0.14 | -7.68 | -8.84 | -6.60 | -5.39 | -1.52 | -0.13 |
| AATT | -8.40 | -9.55 | -5.05 | -5.46 | -1.03 | -0.23 | -8.69 | -8.86 | -5.43 | -5.81 | -1.15 | -0.27 |
| AATC | -9.92 | -9.68 | -4.88 | -5.39 | -1.19 | 0.36 | -9.29 | -7.78 | -5.67 | -6.06 | -1.26 | 0.59 |
| AATG | -8.49 | -10.35 | -5.73 | -5.70 | -1.07 | 1.11 | -10.33 | -10.08 | -5.10 | -5.17 | -1.18 | -0.31 |
| TATA | -8.06 | -9.02 | -6.28 | -5.45 | -2.12 | -0.35 | -8.76 | -8.11 | -5.16 | -3.22 | -1.23 | -0.39 |
| TATC | -8.04 | -9.50 | -5.58 | -6.03 | 0.64 | 1.11 | -7.49 | -8.51 | -5.38 | -5.79 | -1.26 | -0.35 |
| TATG | -9.41 | -8.94 | -5.48 | -6.23 | -1.57 | -0.41 | -8.62 | -10.97 | -5.67 | -5.77 | -1.29 | -0.11 |
| CATC | -9.89 | -7.96 | -5.56 | -5.19 | -1.37 | -0.28 | -9.82 | -8.81 | -5.78 | -4.84 | -0.98 | -0.16 |
| CATG | -10.01 | -8.99 | -6.01 | -5.55 | -1.34 | -0.32 | -9.57 | -4.77 | -4.95 | -3.37 | -1.35 | -0.64 |
| GATC | -8.88 | -6.25 | -5.41 | -5.47 | -1.42 | -0.37 | -9.40 | -8.33 | -5.16 | -5.38 | -0.89 | -0.27 |
| AACA | -17.29 | -8.76 | -4.99 | -4.65 | -0.65 | -0.35 | -16.06 | -10.80 | -4.93 | -4.31 | -2.09 | -0.23 |
| AACT | -16.22 | -9.81 | -4.74 | -4.73 | -2.34 | -0.13 | -19.28 | -8.35 | -5.21 | -5.67 | -0.88 | -0.25 |
| AACC | -16.22 | -8.49 | -5.31 | -5.02 | -2.13 | -0.16 | -19.51 | -6.91 | -6.29 | -3.33 | -1.14 | -0.42 |
| AACG | -16.62 | -10.02 | -5.79 | -4.39 | -1.57 | -0.36 | -16.96 | -3.01 | -5.74 | -5.31 | -2.07 | -0.29 |
| TACA | -12.42 | -6.77 | -5.09 | -4.39 | -3.57 | -0.16 | -17.60 | -10.44 | -4.15 | -4.12 | -1.40 | -0.58 |
| TACT | -14.56 | -9.34 | -4.28 | -4.74 | -2.75 | -0.26 | -15.03 | -11.29 | -4.98 | -4.61 | -2.32 | -0.15 |
| TACC | -14.62 | -9.62 | -4.47 | -5.07 | -1.65 | 0.15 | -16.05 | -10.05 | -3.79 | -5.04 | -2.33 | -0.18 |
| TACG | -18.43 | -9.64 | -5.83 | -5.31 | -2.34 | -0.38 | -17.93 | -8.80 | -5.22 | -4.51 | -2.07 | -0.47 |
| CACA | -16.79 | -11.19 | -4.83 | -5.84 | -2.18 | -0.51 | -16.66 | -10.02 | -3.44 | -4.29 | -2.40 | -0.30 |
| CACT | -15.34 | -12.25 | -4.98 | -4.73 | -0.63 | -1.56 | -15.89 | -7.12 | -5.11 | -4.42 | -2.15 | -0.12 |
| CACC | -15.98 | -9.50 | -4.47 | -5.56 | -1.78 | -0.23 | -16.77 | -8.94 | -4.65 | -4.60 | -1.19 | -0.46 |
| CACG | -15.95 | -9.11 | -6.00 | -3.02 | -2.26 | -0.34 | -18.75 | -8.41 | -5.84 | -4.68 | -2.40 | -0.20 |
| GACA | -15.56 | -9.92 | -5.47 | -5.29 | -2.48 | -0.21 | -17.06 | -9.42 | -5.76 | -4.36 | -1.59 | -0.19 |
| GACT | -16.38 | -8.35 | -5.74 | -5.09 | -0.94 | -0.50 | -15.10 | -9.88 | -5.96 | -4.48 | -1.43 | 0.78 |
| GACC | -17.16 | -8.62 | -5.45 | -4.79 | -1.71 | -0.40 | -19.13 | -9.47 | -5.78 | -4.48 | -1.38 | -0.57 |
| GACG | -14.72 | -6.97 | -5.58 | -5.03 | -2.95 | -0.52 | -15.40 | -8.03 | -4.57 | -2.83 | -2.22 | -0.23 |
| AAGA | -16.64 | -8.20 | -4.22 | -5.15 | -0.60 | -2.58 | -14.63 | -7.88 | -5.28 | -5.10 | -0.31 | -2.20 |
| AAGT | -16.39 | -8.09 | -4.24 | -5.93 | -1.31 | -1.34 | -13.64 | -8.26 | -3.67 | -4.00 | -1.16 | -2.54 |
| AAGC | -16.58 | -9.41 | -3.77 | -3.93 | -1.06 | -1.35 | -12.33 | -9.62 | -3.36 | -4.74 | -0.65 | -2.12 |
| AAGG | -17.42 | -7.80 | -5.11 | -4.28 | -0.53 | -2.18 | -9.83 | -9.14 | -3.81 | -1.39 | -1.87 | -0.21 |
| TAGA | -16.46 | -9.04 | -5.22 | -5.27 | -1.54 | -1.51 | -15.09 | -5.77 | -6.34 | -3.12 | -1.68 | -2.74 |
| TAGT | -15.96 | -7.14 | -3.39 | -5.56 | -0.63 | -2.07 | -18.39 | -8.94 | -3.59 | -4.64 | -1.16 | -1.04 |
| TAGC | -13.87 | -6.68 | -3.82 | -5.88 | -0.08 | -1.48 | -13.95 | -6.75 | -3.93 | -4.48 | -0.30 | -0.55 |
| TAGG | -18.46 | -6.41 | -4.89 | -4.80 | -0.07 | -1.47 | -17.38 | -5.56 | -5.39 | -3.32 | -0.89 | -1.93 |
| CAGA | -18.33 | -9.28 | -5.70 | -4.56 | -1.06 | -1.37 | -13.04 | -10.20 | -5.42 | -3.70 | -1.36 | -1.38 |
| CAGT | -16.14 | -9.54 | -4.76 | -4.82 | -1.64 | -1.27 | -13.27 | -6.19 | -4.88 | -3.23 | -0.34 | -2.56 |
| CAGC | -15.82 | -7.63 | -3.93 | -4.31 | -2.00 | -1.07 | -16.11 | -9.39 | -4.63 | -3.57 | -0.77 | -1.43 |
| CAGG | -18.23 | -10.79 | -5.08 | -4.82 | -0.91 | -1.67 | -18.13 | -10.45 | -4.05 | -5.21 | -0.86 | -1.30 |
| GAGA | -13.57 | -5.27 | -4.18 | -5.68 | -0.50 | -2.07 | -13.27 | -8.98 | -2.97 | -4.89 | -1.15 | -1.49 |
| GAGT | -16.71 | -7.26 | -5.10 | -4.37 | -0.53 | -2.38 | -18.31 | -6.71 | -4.49 | -5.22 | -0.79 | -1.89 |
| GAGC | -9.11 | -3.80 | -6.21 | -4.93 | -2.03 | 0.45 | -15.75 | -10.15 | -4.18 | -5.88 | -1.29 | -1.45 |
| GAGG | -19.46 | -8.45 | -5.15 | -5.39 | -0.98 | -0.39 | -15.39 | -9.42 | -5.42 | -4.00 | -1.27 | -1.97 |
| ATAA | -5.12 | -4.71 | -4.57 | -3.87 | -0.19 | -3.30 | -8.87 | -10.06 | -3.41 | -4.17 | -0.15 | -1.95 |
| ATAT | -7.12 | -10.07 | -4.60 | -5.07 | 0.85 | -1.49 | -7.44 | -2.97 | -3.21 | -3.43 | -0.24 | -2.89 |
| ATAC | -10.61 | -10.23 | -4.67 | -4.49 | 0.81 | -1.58 | -9.61 | -9.26 | -4.72 | -4.88 | 0.04 | -0.12 |
| ATAG | -9.01 | -5.27 | -2.72 | -3.82 | -0.12 | -2.03 | -6.40 | -8.10 | -3.20 | -4.92 | -0.19 | -2.42 |
| TTAA | -10.84 | -9.62 | -4.59 | -3.69 | -0.19 | -1.99 | -8.11 | -6.24 | -4.13 | -4.80 | -0.10 | -3.11 |
| TTAC | -8.32 | -9.14 | -5.03 | -4.86 | -0.27 | -1.69 | -6.36 | -6.44 | -5.02 | -3.79 | -0.65 | -0.81 |
| TTAG | -9.85 | -10.48 | -3.23 | -5.11 | 0.25 | -1.10 | -8.07 | -6.16 | -4.00 | -2.33 | -0.11 | -3.41 |
| CTAC | -10.49 | -9.52 | -4.06 | -4.67 | 0.12 | -1.15 | -9.00 | -10.29 | -4.82 | -4.96 | -0.14 | -3.03 |
| CTAG | -8.43 | -8.41 | -4.33 | -4.02 | -0.16 | -2.88 | -12.15 | -9.02 | -5.89 | -2.29 | -0.32 | -1.90 |
| GTAC | -9.64 | -7.91 | -4.48 | -4.60 | -0.27 | 0.49 | -9.54 | -9.07 | -3.16 | -3.37 | -0.10 | -2.96 |
| ATCA | -17.20 | -7.48 | -4.36 | -4.40 | -0.29 | -0.57 | -16.01 | -2.47 | -2.87 | -3.31 | -0.19 | -2.54 |
| ATCT | -18.05 | -10.11 | -6.62 | -4.37 | -0.26 | -0.94 | -15.90 | -9.26 | -5.71 | -4.70 | -0.26 | -1.60 |
| ATCC | -16.91 | -9.87 | -6.14 | -4.97 | -0.08 | -0.44 | -17.11 | -10.26 | -6.70 | -4.51 | -0.04 | -0.71 |
| ATCG | -17.29 | -8.03 | -6.38 | -4.41 | -0.42 | -0.85 | -18.03 | -10.98 | -6.23 | -4.80 | -0.29 | -1.01 |
| TTCA | -16.32 | -9.46 | -5.69 | -4.68 | -0.52 | -0.39 | -16.44 | -9.22 | -5.16 | -3.67 | 0.73 | -0.50 |
| TTCT | -17.28 | -8.64 | -5.92 | -3.20 | -0.42 | -1.40 | -17.33 | -8.18 | -5.30 | -3.67 | -0.21 | -2.47 |
| TTCC | -14.64 | -8.56 | -5.31 | -3.09 | -1.09 | -0.41 | -14.74 | -8.98 | -4.96 | -5.03 | -0.47 | -0.90 |
| TTCG | -19.83 | -10.54 | -5.00 | -4.81 | 0.43 | -0.66 | -16.02 | -8.56 | -5.59 | -4.33 | -0.73 | -0.57 |
| CTCA | -18.62 | -10.60 | -4.81 | -4.73 | -0.47 | -1.32 | -14.71 | -10.30 | -6.42 | -3.85 | -0.30 | -0.84 |
| CTCT | -14.84 | -10.27 | -5.78 | -4.10 | -0.59 | -1.79 | -18.84 | -8.48 | -4.10 | -4.57 | -0.23 | -2.18 |
| CTCC | -16.98 | -8.46 | -5.29 | -4.26 | -0.18 | -0.92 | -17.52 | -8.07 | -5.25 | -3.60 | -0.06 | -1.54 |
| CTCG | -15.21 | -9.79 | -5.74 | -3.34 | -0.23 | -1.49 | -16.29 | -11.65 | -6.45 | -4.87 | -0.07 | -0.87 |
| GTCA | -20.81 | -5.87 | -5.02 | -4.25 | 0.59 | -0.55 | -14.79 | -7.63 | -5.72 | -3.04 | -1.05 | -1.29 |
| GTCT | -19.60 | -7.13 | -5.77 | -4.59 | -0.66 | -1.35 | -16.85 | -9.49 | -5.94 | -4.17 | -0.43 | -1.22 |
| GTCC | -18.28 | -7.90 | -5.57 | -4.52 | -0.28 | -1.59 | -15.48 | -10.29 | -5.59 | -4.15 | 1.03 | -1.00 |
| GTCG | -16.71 | -10.10 | -6.44 | -5.34 | -0.43 | -0.88 | -17.25 | -7.33 | -6.95 | -3.88 | -0.43 | -0.96 |
| ATGA | -14.05 | -10.29 | -3.07 | -4.72 | -0.27 | -3.23 | -16.93 | -7.10 | -2.36 | -4.74 | -0.48 | -2.84 |
| ATGT | -18.94 | -7.41 | -4.06 | -4.67 | -0.25 | -4.61 | -18.25 | -6.03 | -4.13 | -1.66 | -1.20 | -3.56 |
| ATGC | -15.13 | -7.30 | -4.18 | -3.00 | -0.26 | -4.26 | -18.27 | -8.99 | -4.44 | -2.88 | -0.84 | -2.32 |
| ATGG | -18.58 | -11.31 | -3.78 | -3.93 | -2.02 | -2.64 | -13.45 | -8.64 | -4.73 | -2.83 | -0.13 | -4.56 |
| TTGA | -15.89 | -9.07 | -3.58 | -3.94 | -1.14 | -2.25 | -11.97 | -8.30 | -2.67 | -4.13 | -0.62 | -3.28 |
| TTGT | -15.63 | -5.03 | -4.14 | -3.89 | -0.25 | -4.08 | -15.03 | -8.15 | -1.37 | -5.37 | -0.31 | -2.59 |
| TTGC | -17.80 | -10.03 | -4.27 | -3.98 | -0.66 | -3.53 | -17.93 | -10.00 | -3.66 | -4.71 | -0.15 | -3.06 |
| TTGG | -16.28 | -8.81 | -3.49 | -4.57 | -0.52 | -3.84 | -18.24 | -9.47 | -3.83 | -3.62 | -0.31 | -4.13 |
| CTGA | -18.43 | -9.09 | -4.23 | -4.47 | -0.38 | -2.63 | -18.25 | -8.80 | -2.50 | -4.73 | -0.69 | -2.95 |
| CTGT | -15.62 | -9.00 | -4.51 | -4.20 | -0.56 | -3.20 | -16.87 | -9.42 | -4.09 | -2.98 | -1.73 | -2.41 |
| CTGC | -16.04 | -9.59 | -4.17 | -3.22 | -1.57 | -2.90 | -18.20 | -8.73 | -3.56 | -4.52 | -0.36 | -3.85 |
| CTGG | -13.54 | -10.49 | -3.58 | -4.26 | -0.77 | -2.71 | -18.26 | -6.60 | -3.69 | -3.76 | -0.35 | -3.95 |
| GTGA | -19.16 | -7.84 | -3.32 | -4.83 | -0.59 | -1.87 | -14.51 | -8.48 | -3.09 | -4.01 | -0.20 | -4.13 |
| GTGT | -15.97 | -7.29 | -3.69 | -4.54 | -0.76 | -2.71 | -17.09 | -7.16 | -3.33 | -4.06 | -0.42 | -4.61 |
| GTGC | -20.12 | -9.16 | -2.19 | -4.08 | -0.39 | -3.75 | -15.96 | -7.76 | -4.27 | -3.69 | -0.30 | -3.88 |
| GTGG | -17.63 | -10.81 | -2.62 | -5.34 | -0.53 | -3.19 | -18.99 | -7.44 | -3.95 | -3.78 | -0.24 | -3.09 |
| ACCA | -16.93 | -17.26 | -5.81 | -3.87 | -0.72 | -2.62 | -16.99 | -13.35 | -5.85 | -3.46 | -0.51 | -2.35 |
| ACCT | -13.11 | -17.58 | -5.43 | -3.44 | -1.35 | -2.47 | -16.94 | -14.08 | -5.70 | -3.03 | -0.77 | -2.05 |
| ACCC | -16.47 | -17.81 | -5.95 | -4.32 | -0.43 | -2.94 | -19.56 | -18.03 | -5.53 | -3.41 | -0.78 | -1.80 |
| ACCG | -13.43 | -11.67 | -4.15 | -3.10 | -0.81 | -3.81 | -17.73 | -18.86 | -5.07 | -2.41 | -0.21 | -3.81 |
| TCCA | -17.34 | -12.77 | -3.47 | -3.08 | -3.07 | -1.20 | -16.71 | -16.02 | -3.00 | -3.63 | -5.17 | -2.39 |
| TCCT | -18.98 | -16.40 | -4.96 | -4.18 | -0.42 | -1.91 | -14.56 | -16.22 | -5.42 | -3.59 | -0.45 | -1.80 |
| TCCC | -17.17 | -20.45 | -5.48 | -3.92 | -0.67 | -2.85 | -17.54 | -18.12 | -5.10 | -3.75 | -1.90 | -1.26 |
| TCCG | -16.22 | -14.51 | -6.76 | -3.71 | -0.50 | -1.95 | -16.58 | -21.85 | -5.96 | -2.06 | -0.64 | -3.04 |
| CCCA | -18.40 | -17.11 | -5.15 | -4.27 | -1.00 | -2.43 | -16.44 | -15.01 | -5.61 | -2.65 | -0.22 | -3.44 |
| CCCT | -16.59 | -19.24 | -5.88 | -3.56 | -0.92 | -0.82 | -14.95 | -13.77 | -7.60 | -2.81 | -0.41 | -1.80 |
| CCCC | -19.48 | -16.38 | -6.02 | -3.18 | -0.58 | -3.04 | -13.07 | -18.30 | -4.63 | -4.04 | -0.35 | -2.95 |
| CCCG | -15.28 | -14.28 | -5.37 | -4.10 | -1.39 | -1.00 | -17.78 | -15.29 | -0.20 | -3.63 | -1.37 | -1.80 |
| GCCA | -14.98 | -21.78 | -5.83 | -4.17 | -0.96 | -1.46 | -19.19 | -14.20 | -5.54 | -2.37 | -1.76 | -1.49 |
| GCCT | -17.88 | -17.08 | -3.80 | -4.45 | -0.88 | -2.79 | -19.87 | -16.54 | -4.54 | -3.41 | -1.57 | -2.20 |
| GCCC | -18.34 | -18.58 | -5.21 | -2.89 | -1.32 | -0.84 | -16.58 | -14.91 | -4.75 | -2.57 | -0.25 | -3.45 |
| GCCG | -20.06 | -17.03 | -5.03 | -2.51 | -1.70 | -1.50 | -18.26 | -14.31 | -5.03 | -4.06 | -0.55 | -2.25 |
| ACGA | -6.95 | -17.72 | -5.37 | -2.80 | -0.89 | -3.58 | -18.93 | -19.18 | -2.89 | -5.15 | -0.84 | -3.20 |
| ACGT | -14.56 | -15.41 | -2.07 | -3.69 | -0.37 | -5.49 | -16.52 | -18.05 | -2.20 | -3.15 | -0.35 | -4.70 |
| ACGC | -18.58 | -18.48 | -2.61 | -3.45 | -0.48 | -4.70 | -27.15 | -15.70 | -5.74 | -2.98 | 1.28 | -4.34 |
| ACGG | -10.37 | -19.31 | -3.75 | -3.33 | -1.21 | -2.17 | -16.42 | -19.71 | -3.36 | -3.33 | -0.30 | -4.64 |
| TCGA | -13.60 | -14.75 | -4.50 | -4.02 | -0.52 | -3.52 | -17.04 | -17.08 | -3.87 | -2.37 | -0.97 | -3.58 |
| TCGC | -15.70 | -16.08 | -2.68 | -3.93 | -0.59 | -3.37 | -16.88 | -18.02 | -3.70 | -3.53 | -0.92 | -2.74 |
| TCGG | -14.51 | -17.34 | -3.42 | -3.61 | -0.87 | -2.19 | -18.06 | -19.22 | -3.94 | -3.20 | -0.47 | -4.10 |
| CCGC | -16.75 | -15.64 | -4.58 | -5.43 | -1.09 | -0.31 | -18.69 | -19.88 | -4.70 | -1.44 | -1.03 | -3.88 |
| CCGG | -17.83 | -9.60 | -4.42 | -5.06 | -0.25 | -3.20 | -18.86 | -18.99 | -3.75 | -0.83 | -0.37 | -4.55 |
| GCGC | -15.27 | -20.29 | -3.66 | -3.51 | -1.22 | -2.75 | -17.53 | -17.58 | -2.07 | -3.95 | -1.11 | -3.37 |
| AGCA | -14.28 | -17.64 | -5.37 | -3.85 | -2.89 | -0.09 | -18.21 | -18.31 | -6.43 | -6.70 | -0.52 | 1.28 |
| AGCT | -16.91 | -19.36 | -5.29 | -5.30 | -0.72 | -0.04 | -20.17 | -13.95 | -5.47 | -5.68 | -0.49 | -0.13 |
| AGCC | -17.32 | -18.66 | -5.77 | -6.29 | -0.64 | -0.16 | -11.08 | -15.72 | -4.72 | -3.74 | -0.61 | -0.16 |
| AGCG | -16.34 | -18.14 | -5.99 | -4.42 | -1.73 | -0.15 | -17.54 | -15.40 | -6.01 | -6.48 | -0.74 | -0.27 |
| TGCA | -18.47 | -19.06 | -4.23 | -4.64 | -3.40 | -0.05 | -16.56 | -15.07 | -4.44 | -4.37 | -2.34 | -0.08 |
| TGCC | -14.25 | -20.24 | -3.85 | -4.93 | 0.34 | 1.10 | -17.54 | -12.03 | -5.04 | -5.40 | 1.14 | 0.43 |
| TGCG | -19.06 | -14.76 | -5.58 | -5.71 | -1.62 | -0.10 | -16.72 | -14.46 | -5.24 | -5.81 | -0.39 | -0.18 |
| CGCC | -19.34 | -16.77 | -5.79 | -5.57 | -0.52 | -0.17 | -25.34 | -15.60 | -9.64 | -5.36 | 2.79 | -0.06 |
| CGCG | -13.90 | -19.68 | -6.04 | -6.21 | -0.84 | -0.27 | -17.98 | -15.38 | -5.84 | -7.20 | -0.94 | -0.20 |
| GGCC | -18.45 | -17.55 | -5.86 | -6.15 | -1.29 | -0.04 | -17.88 | -12.27 | -4.45 | -3.03 | -0.49 | 0.80 |
| AAA) | -10.25 | -9.22 | -5.79 | -4.37 | -0.84 | -1.63 | -10.22 | -9.63 | -5.50 | -4.38 | -0.56 | -1.84 |
| TAA) | -10.57 | -10.65 | -6.59 | -5.41 | -1.43 | -0.85 | -9.64 | -8.69 | -4.65 | -3.89 | -2.12 | -0.18 |
| CAA) | -8.03 | -10.72 | -6.66 | -3.60 | -0.68 | -0.57 | -9.36 | -9.57 | -5.86 | -4.98 | -1.13 | -1.27 |
| GAA) | -10.75 | -9.25 | -4.36 | -5.02 | -0.55 | -2.03 | -9.53 | -8.04 | -4.25 | -2.58 | -0.50 | -1.60 |
| (AAA | -9.54 | -7.04 | -5.44 | -4.96 | -1.34 | -2.34 | -4.99 | -8.15 | -5.04 | -3.61 | -0.15 | -2.27 |
| (AAT | -10.21 | -7.65 | -4.01 | -5.43 | -1.16 | -1.58 | -10.77 | -9.76 | -4.27 | -4.98 | -0.73 | -1.93 |
| (AAC | -8.51 | -8.76 | -5.41 | -5.27 | -1.84 | -1.73 | -10.43 | -7.64 | -4.47 | -4.98 | -0.86 | -2.17 |
| (AAG | -7.68 | -10.05 | -4.69 | -3.94 | -1.38 | -0.83 | -10.36 | -10.73 | -5.64 | -3.59 | -1.19 | -1.14 |
| AAT) | -9.73 | -9.64 | -5.61 | -5.42 | -1.15 | -0.31 | -7.44 | -5.17 | -6.70 | -0.40 | -0.26 | -1.29 |
| TAT) | -9.12 | -6.48 | -5.16 | -5.35 | -1.60 | -0.37 | -9.63 | -4.25 | -4.62 | -3.74 | -1.64 | -0.22 |
| CAT) | -9.11 | -9.32 | -5.54 | -5.95 | -1.08 | -0.32 | -9.48 | -9.48 | -5.70 | -5.63 | -1.47 | -0.43 |
| GAT) | -9.58 | -7.91 | -6.03 | -5.98 | -1.20 | -0.37 | -8.84 | -2.03 | -5.32 | -4.72 | -1.40 | -0.29 |
| AAC) | -8.09 | -18.09 | -5.61 | -5.76 | -0.92 | -0.23 | -8.59 | -18.06 | -4.98 | -4.99 | -1.29 | -0.33 |
| TAC) | -10.67 | -18.01 | -5.04 | -5.00 | -2.19 | -0.23 | -10.82 | -18.49 | -5.35 | -5.18 | -3.42 | -0.10 |
| CAC) | -9.76 | -18.13 | -5.05 | -5.25 | -2.26 | -0.32 | -8.22 | -17.88 | -5.46 | -4.65 | -3.11 | -0.14 |
| GAC) | -8.86 | -15.32 | -6.01 | -4.35 | -2.57 | -0.22 | -9.40 | -18.61 | -5.22 | -4.63 | -2.39 | -0.23 |
| (ACA | -16.69 | -8.88 | -5.49 | -4.15 | -1.59 | -0.51 | -14.29 | -10.97 | -6.04 | -4.06 | -1.90 | -0.28 |
| (ACT | -16.94 | -8.06 | -4.43 | -4.94 | -1.50 | -0.22 | -16.60 | -8.29 | -6.15 | -4.05 | -2.01 | -0.21 |
| (ACC | -18.84 | -10.31 | -5.61 | -4.75 | -2.03 | -0.44 | -3.26 | -0.40 | -4.92 | -4.38 | -0.37 | -0.07 |
| (ACG | -15.29 | -8.87 | -3.99 | -4.68 | -1.30 | -0.61 | -18.76 | -10.40 | -4.51 | -4.10 | -2.21 | -0.23 |
| AAG) | -9.37 | -15.43 | -5.36 | -4.66 | -0.97 | -1.96 | -8.89 | -19.68 | -4.43 | -5.37 | -0.78 | -2.07 |
| TAG) | -9.90 | -19.32 | -4.49 | -4.80 | -0.84 | -1.91 | -9.47 | -8.39 | -4.34 | -4.64 | -0.79 | -0.90 |
| CAG) | -7.64 | -17.21 | -3.87 | -4.21 | -1.36 | -1.39 | -7.72 | -18.63 | -5.40 | -4.57 | -0.65 | -0.85 |
| GAG) | -9.46 | -19.55 | -5.48 | -4.00 | -0.65 | -2.86 | -9.41 | -17.76 | -3.98 | -3.72 | -0.44 | -1.85 |
| (AGA | -17.11 | -3.95 | -5.30 | -4.86 | -0.41 | -1.31 | -15.88 | -3.71 | -3.39 | -6.81 | -1.96 | -0.22 |
| (AGT | -16.16 | -6.92 | -4.58 | -2.90 | -0.80 | -1.35 | -18.62 | -6.95 | -4.95 | -4.14 | -0.58 | -1.55 |
| (AGC | -16.09 | -10.48 | -3.73 | -5.05 | -1.26 | -1.35 | -15.25 | -9.79 | -4.11 | -3.95 | -1.14 | -1.43 |
| (AGG | -19.81 | -9.46 | -4.45 | -5.21 | -1.02 | -1.08 | -18.31 | -6.88 | -5.35 | -3.08 | -0.96 | -3.11 |
| ATA) | -7.12 | -9.55 | -5.25 | -3.24 | -0.50 | -1.68 | -5.84 | -1.60 | -4.08 | -0.54 | -0.33 | -1.51 |
| TTA) | -9.26 | -9.55 | -5.01 | -4.23 | -0.13 | -0.71 | -8.41 | -6.99 | -2.59 | -4.35 | -0.24 | -2.27 |
| CTA) | -9.40 | -7.31 | -3.72 | -4.00 | -0.23 | -2.34 | -8.24 | -0.34 | -2.57 | -4.92 | -1.92 | -0.02 |
| GTA) | -5.16 | -9.58 | -5.88 | -3.30 | -0.91 | -1.33 | -10.57 | -9.95 | -4.89 | -3.18 | 0.09 | -0.92 |
| ATC) | -6.17 | -16.78 | -4.11 | -5.22 | -1.18 | -0.32 | -8.70 | -14.43 | -4.32 | -6.90 | -0.16 | -0.01 |
| TTC) | -9.93 | -16.04 | -4.03 | -5.72 | -1.20 | -0.55 | -9.86 | -12.76 | -4.12 | -3.65 | -0.91 | -0.29 |
| CTC) | -3.22 | -17.26 | -5.18 | -6.10 | -0.42 | -0.72 | -9.29 | -19.59 | -5.15 | -6.13 | -0.42 | -0.82 |
| GTC) | -8.82 | -16.70 | -4.68 | -5.35 | -1.61 | -0.36 | -4.99 | -15.30 | -5.65 | -5.00 | -1.60 | -0.48 |
| (TCA | -15.71 | -11.31 | -5.37 | -4.61 | -0.31 | -0.96 | -17.11 | -8.14 | -6.55 | -4.57 | -0.29 | -0.38 |
| (TCT | -19.31 | -8.55 | -6.53 | -3.25 | -0.23 | -1.54 | -15.35 | -7.97 | -6.15 | -4.36 | -0.44 | -1.08 |
| (TCC | -19.43 | -10.33 | -6.85 | -3.96 | -0.37 | -0.96 | -17.52 | -9.83 | -6.79 | -3.45 | -0.40 | -0.99 |
| (TCG | -19.17 | -9.51 | -4.41 | -4.54 | -0.63 | -0.95 | -13.94 | -9.61 | -6.22 | -5.00 | 0.19 | -0.72 |
| ATG) | -10.76 | -20.55 | -5.25 | -2.55 | -0.65 | -3.32 | -7.10 | -15.16 | -3.85 | -3.17 | -0.60 | -4.79 |
| TTG) | -7.25 | -14.86 | -4.32 | -2.97 | -0.53 | -3.08 | -8.41 | -18.45 | -3.39 | -3.26 | -0.23 | -4.58 |
| CTG) | -10.40 | -17.25 | -3.81 | -4.39 | -1.02 | -2.19 | -10.09 | -15.36 | -3.29 | -4.23 | -0.77 | -2.85 |
| GTG) | -8.86 | -16.49 | -3.96 | -3.65 | -1.06 | -2.21 | -7.97 | -18.54 | -5.17 | -2.20 | -0.52 | -3.95 |
| (TGA | -14.66 | -8.95 | -4.40 | -3.60 | -0.26 | -3.61 | -18.15 | -9.43 | -4.39 | -5.65 | -0.32 | -3.01 |
| (TGT | -16.52 | -10.46 | -3.94 | -5.35 | -0.95 | -1.87 | -17.65 | -0.28 | -0.92 | -5.75 | -1.44 | -0.03 |
| (TGC | -14.86 | -9.50 | -3.75 | -3.00 | -1.45 | -1.46 | -18.72 | -9.21 | -4.80 | -3.26 | -1.34 | -2.72 |
| (TGG | -15.78 | -8.85 | -4.21 | -4.63 | -0.26 | -3.38 | -13.10 | 0.01 | -3.94 | 0.00 | 0.01 | -0.33 |
| ACC) | -14.52 | -18.81 | -3.95 | -5.77 | -2.59 | -0.60 | -17.30 | -18.47 | -3.14 | -3.34 | -2.01 | -2.08 |
| TCC) | -16.69 | -19.51 | -4.36 | -5.82 | -1.30 | -1.34 | -17.60 | -17.24 | -3.21 | -5.83 | -2.04 | -1.19 |
| CCC) | -19.47 | -19.46 | -4.32 | -5.73 | -1.63 | -1.94 | -20.12 | -17.43 | -4.50 | -3.99 | -0.26 | -3.74 |
| GCC) | -21.76 | -18.79 | -3.97 | -4.76 | -1.85 | -0.67 | -18.79 | -20.20 | -3.92 | -5.42 | -0.54 | -3.34 |
| (CCA | -18.33 | -20.35 | -5.64 | -4.18 | -0.98 | -2.00 | -17.28 | -17.25 | -4.25 | -3.85 | -0.78 | -2.20 |
| (CCT | -16.03 | -16.85 | -3.41 | -3.80 | -0.33 | -4.07 | -17.55 | -16.84 | -5.29 | -3.02 | -0.49 | -2.56 |
| (CCC | -18.94 | -18.15 | -5.56 | -2.56 | -0.80 | -0.91 | -17.38 | -17.35 | -4.70 | -3.04 | -0.36 | -3.01 |
| (CCG | -15.68 | -16.91 | -5.63 | -4.09 | -0.89 | -2.42 | -29.59 | -13.79 | -3.04 | -4.16 | -6.22 | -1.93 |
| ACG) | -17.61 | -15.32 | -4.69 | -2.17 | -0.60 | -4.56 | -14.17 | -19.25 | -3.83 | -2.37 | -0.41 | -4.64 |
| TCG) | -18.87 | -14.08 | -4.58 | -3.08 | 0.41 | -3.87 | -17.20 | -18.88 | -4.68 | -2.37 | -0.65 | -3.28 |
| CCG) | -13.10 | -18.63 | -2.18 | -4.78 | -0.56 | -3.03 | -15.53 | -12.71 | -4.17 | -1.78 | -0.59 | -4.13 |
| GCG) | -18.25 | -16.17 | -3.14 | -2.34 | -1.02 | -4.35 | -16.06 | -18.55 | -4.23 | -2.43 | -0.93 | -4.37 |
| AGC) | -17.82 | -16.05 | -5.95 | -5.40 | -0.27 | 0.32 | -18.67 | -15.66 | -5.44 | -5.46 | 0.23 | 0.13 |
| TGC) | -16.80 | -17.57 | -4.69 | -5.83 | -2.54 | -0.06 | -18.16 | -17.78 | -5.43 | -6.03 | -2.23 | -0.14 |
| CGC) | -16.57 | -18.51 | -5.23 | -5.87 | -1.50 | -0.19 | -19.00 | -18.53 | -5.82 | -4.97 | -1.22 | -0.25 |
| GGC) | -16.68 | -18.09 | -4.95 | -4.96 | -0.88 | -0.08 | -18.41 | -14.64 | -6.55 | -5.72 | -1.18 | -0.28 |

**Table S3.** Interaction energies of 32 dangling terms. All values are reported in kcal/mol.

|  | a_hb1 | a_sk1 | a_sk2 | b_hb1 | b_sk1 | b_sk2 |  | a_hb1 | a_sk1 | a_sk2 | b_hb1 | b_sk1 | b_sk2 |
| --- | --- | --- | --- | --- | --- | --- | --- | --- | --- | --- | --- | --- | --- |
| A)A | -9.35 | -4.26 | -3.15 | -9.58 | -3.00 | -4.66 | A(A | -9.53 | -5.39 | -1.28 | -9.77 | -4.16 | -0.33 |
| T)A | -9.03 | -5.17 | -2.16 | -4.97 | -4.54 | 1.63 | T(A | -8.77 | -5.57 | -0.65 | 0.01 | -5.72 | 0.00 |
| C)A | -11.99 | -1.51 | -4.61 | -19.12 | -3.89 | -4.16 | C(A | -10.20 | -4.62 | -0.46 | -8.93 | -0.04 | 0.01 |
| G)A | -15.24 | -5.13 | -2.63 | -14.46 | -4.67 | -4.55 | G(A | -8.55 | -6.34 | -2.30 | -0.08 | -5.17 | -0.30 |
| A)T | -10.46 | -2.81 | -3.34 | -9.09 | -5.92 | -0.34 | A(T | -10.64 | -5.42 | -1.75 | -7.29 | -4.30 | -3.86 |
| T)T | -10.39 | -5.54 | -0.51 | -9.94 | -4.62 | -0.77 | T(T | -9.21 | -4.25 | -1.79 | -10.19 | -0.20 | 0.00 |
| C)T | -17.59 | -3.46 | -2.25 | -15.73 | -3.18 | -2.93 | C(T | -10.97 | -4.56 | -0.34 | -9.42 | -4.92 | -1.16 |
| G)T | -16.70 | -5.71 | -0.64 | -18.62 | -6.78 | -0.27 | G(T | -12.27 | -5.04 | -3.78 | -9.51 | -6.20 | -2.93 |
| A)C | -5.87 | -4.68 | -0.33 | -6.29 | -2.67 | -0.15 | A(C | -19.28 | -4.36 | -3.55 | -18.53 | -4.98 | -0.29 |
| T)C | -10.97 | -4.52 | -0.42 | -9.42 | -5.23 | -2.60 | T(C | -15.11 | -5.48 | -2.41 | -17.94 | -5.88 | -2.02 |
| C)C | -16.99 | -2.22 | -2.91 | -13.43 | -3.13 | -0.61 | C(C | -18.25 | -4.12 | -0.78 | -18.87 | -3.08 | -0.79 |
| G)C | -15.48 | -4.91 | -2.95 | -17.37 | -5.03 | -2.33 | G(C | -14.79 | -5.20 | -3.78 | -19.01 | -4.40 | -5.07 |
| A)G | -5.47 | -5.44 | -2.94 | -3.56 | -5.58 | -3.64 | A(G | -19.29 | -6.60 | -2.01 | -18.02 | -6.83 | -1.10 |
| T)G | -10.03 | -4.39 | -3.28 | -6.52 | -5.41 | -2.39 | T(G | -16.49 | -5.65 | -0.22 | -16.92 | -6.54 | -0.36 |
| C)G | -17.04 | -4.72 | -3.85 | -16.54 | -5.14 | -2.09 | C(G | -15.64 | -5.72 | -0.17 | -18.96 | -3.06 | -0.07 |
| G)G | -17.49 | -5.78 | -4.14 | -15.94 | -5.19 | -0.86 | G(G | -15.29 | -5.74 | -2.39 | -16.61 | -6.71 | -2.91 |

**Table S4.** “Energy” of 400 stacking terms and 32 dangling terms. All values are reported in kcal/mol.

|  | energy |  | energy |  | energy |  | energy |
| --- | --- | --- | --- | --- | --- | --- | --- |
| AAAA | -30.75 | AATA | -30.76 | AACA | -37.55 | AAGA | -36.40 |
| AAAT | -28.08 | AATT | -29.95 | AACT | -38.80 | AAGT | -35.28 |
| AAAC | -27.01 | AATC | -30.08 | AACC | -37.46 | AAGC | -34.46 |
| AAAG | -29.44 | AATG | -31.20 | AACG | -36.06 | AAGG | -31.79 |
| AAA) | -32.12 | AAT) | -26.56 | AAC) | -38.47 | AAG) | -39.48 |
| TAAA | -32.15 | TATA | -29.07 | TACA | -35.34 | TAGA | -36.89 |
| TAAT | -28.58 | TATT | -30.76 | TACT | -37.15 | TAGT | -36.25 |
| TAAC | -29.78 | TATC | -28.09 | TACC | -36.35 | TAGC | -30.89 |
| TAAG | -32.39 | TATG | -32.23 | TACG | -40.46 | TAGG | -35.28 |
| TAA) | -32.34 | TAT) | -26.09 | TAC) | -42.25 | TAG) | -34.89 |
| CAAA | -27.78 | CATA | -32.23 | CACA | -39.23 | CAGA | -37.70 |
| CAAT | -30.64 | CATT | -31.20 | CACT | -37.15 | CAGT | -34.32 |
| CAAC | -27.97 | CATC | -30.32 | CACC | -37.06 | CAGC | -35.32 |
| CAAG | -27.68 | CATG | -28.44 | CACG | -38.48 | CAGG | -40.75 |
| CAA) | -31.20 | CAT) | -31.75 | CAC) | -40.11 | CAG) | -36.75 |
| GAAA | -29.41 | GATA | -28.09 | GACA | -38.66 | GAGA | -32.01 |
| GAAT | -26.97 | GATT | -30.08 | GACT | -36.53 | GAGT | -36.88 |
| GAAC | -30.23 | GATC | -28.62 | GACC | -39.47 | GAGC | -32.16 |
| GAAG | -29.78 | GATG | -30.32 | GACG | -34.53 | GAGG | -38.64 |
| GAA) | -29.22 | GAT) | -26.83 | GAC) | -38.91 | GAG) | -39.57 |
| (AAA | -27.43 | (ATA | -26.09 | (ACA | -37.42 | (AGA | -32.45 |
| (AAT | -31.23 | (ATT | -26.56 | (ACT | -36.69 | (AGT | -34.75 |
| (AAC | -31.03 | (ATC | -26.83 | (ACC | -27.69 | (AGC | -36.82 |
| (AAG | -30.61 | (ATG | -31.75 | (ACG | -37.48 | (AGG | -39.36 |
| (AA) | -29.74 | (AT) | -29.33 | (AC) | -37.47 | (AG) | -35.80 |
| ATAA | -25.18 | ATTA | -28.58 | ATCA | -30.85 | ATGA | -35.04 |
| ATAT | -23.84 | ATTT | -28.08 | ATCT | -38.89 | ATGT | -37.38 |
| ATAC | -29.66 | ATTC | -26.97 | ATCC | -38.87 | ATGC | -35.94 |
| ATAG | -24.10 | ATTG | -30.64 | ATCG | -39.36 | ATGG | -38.30 |
| ATA) | -20.62 | ATT) | -31.23 | ATC) | -34.15 | ATG) | -38.87 |
| TTAA | -28.71 | TTTA | -32.15 | TTCA | -35.65 | TTGA | -33.41 |
| TTAT | -25.18 | TTTT | -30.75 | TTCT | -37.01 | TTGT | -32.91 |
| TTAC | -26.19 | TTTC | -29.41 | TTCC | -34.08 | TTGC | -39.88 |
| TTAG | -26.80 | TTTG | -27.78 | TTCG | -38.11 | TTGG | -38.56 |
| TTA) | -26.87 | TTT) | -27.43 | TTC) | -34.52 | TTG) | -35.66 |
| CTAA | -26.80 | CTTA | -32.39 | CTCA | -38.48 | CTGA | -38.57 |
| CTAT | -24.10 | CTTT | -29.44 | CTCT | -37.88 | CTGT | -37.29 |
| CTAC | -31.00 | CTTC | -29.78 | CTCC | -36.06 | CTGC | -38.35 |
| CTAG | -29.90 | CTTG | -27.68 | CTCG | -37.99 | CTGG | -35.98 |
| CTA) | -22.50 | CTT) | -30.61 | CTC) | -37.15 | CTG) | -37.82 |
| GTAA | -26.19 | GTTA | -29.78 | GTCA | -34.72 | GTGA | -36.02 |
| GTAT | -29.66 | GTTT | -27.01 | GTCT | -38.61 | GTGT | -35.82 |
| GTAC | -27.30 | GTTC | -30.23 | GTCC | -36.81 | GTGC | -37.77 |
| GTAG | -31.00 | GTTG | -27.97 | GTCG | -38.35 | GTGG | -38.80 |
| GTA) | -27.79 | GTT) | -31.03 | GTC) | -35.27 | GTG) | -37.28 |
| (TAA | -26.87 | (TTA | -32.34 | (TCA | -37.66 | (TGA | -38.22 |
| (TAT | -20.62 | (TTT | -32.12 | (TCT | -37.38 | (TGT | -32.58 |
| (TAC | -27.79 | (TTC | -29.22 | (TCC | -40.42 | (TGC | -37.04 |
| (TAG | -22.50 | (TTG | -31.20 | (TCG | -37.25 | (TGG | -27.23 |
| (TA) | -26.30 | (TT) | -29.74 | (TC) | -36.90 | (TG) | -36.45 |
| ACAA | -32.91 | ACTA | -36.25 | ACCA | -44.86 | ACGA | -43.74 |
| ACAT | -37.38 | ACTT | -35.28 | ACCT | -42.97 | ACGT | -43.28 |
| ACAC | -35.82 | ACTC | -36.88 | ACCC | -48.51 | ACGC | -51.47 |
| ACAG | -37.29 | ACTG | -34.32 | ACCG | -42.53 | ACGG | -43.95 |
| ACA) | -32.58 | ACT) | -34.75 | ACC) | -46.29 | ACG) | -44.81 |
| TCAA | -33.41 | TCTA | -36.89 | TCCA | -43.93 | TCGA | -42.90 |
| TCAT | -35.04 | TCTT | -36.40 | TCCT | -44.45 | TCGT | -43.74 |
| TCAC | -36.02 | TCTC | -32.01 | TCCC | -49.11 | TCGC | -44.07 |
| TCAG | -38.57 | TCTG | -37.70 | TCCG | -46.88 | TCGG | -45.46 |
| TCA) | -38.22 | TCT) | -32.45 | TCC) | -48.06 | TCG) | -45.57 |
| CCAA | -38.56 | CCTA | -35.28 | CCCA | -45.86 | CCGA | -45.46 |
| CCAT | -38.30 | CCTT | -31.79 | CCCT | -44.18 | CCGT | -43.95 |
| CCAC | -38.80 | CCTC | -38.64 | CCCC | -46.01 | CCGC | -46.70 |
| CCAG | -35.98 | CCTG | -40.75 | CCCG | -40.74 | CCGG | -43.85 |
| CCA) | -27.23 | CCT) | -39.36 | CCC) | -51.30 | CCG) | -40.59 |
| GCAA | -39.88 | GCTA | -30.89 | GCCA | -46.86 | GCGA | -44.07 |
| GCAT | -35.94 | GCTT | -34.46 | GCCT | -47.50 | GCGT | -51.47 |
| GCAC | -37.77 | GCTC | -32.16 | GCCC | -44.84 | GCGC | -46.16 |
| GCAG | -38.35 | GCTG | -35.32 | GCCG | -46.15 | GCGG | -46.70 |
| GCA) | -37.04 | GCT) | -36.82 | GCC) | -52.00 | GCG) | -45.92 |
| (CAA | -35.66 | (CTA | -34.89 | (CCA | -48.54 | (CGA | -45.57 |
| (CAT | -38.87 | (CTT | -39.48 | (CCT | -45.12 | (CGT | -44.81 |
| (CAC | -37.28 | (CTC | -39.57 | (CCC | -46.38 | (CGC | -45.92 |
| (CAG | -37.82 | (CTG | -36.75 | (CCG | -52.17 | (CGG | -40.59 |
| (CA) | -36.45 | (CT) | -35.80 | (CC) | -46.47 | (CG) | -45.03 |
| AGAA | -37.01 | AGTA | -37.15 | AGCA | -46.50 | AGGA | -44.45 |
| AGAT | -38.89 | AGTT | -38.80 | AGCT | -46.75 | AGGT | -42.97 |
| AGAC | -38.61 | AGTC | -36.53 | AGCC | -42.44 | AGGC | -47.50 |
| AGAG | -37.88 | AGTG | -37.15 | AGCG | -46.60 | AGGG | -44.18 |
| AGA) | -37.38 | AGT) | -36.69 | AGC) | -45.01 | AGG) | -45.12 |
| TGAA | -35.65 | TGTA | -35.34 | TGCA | -46.35 | TGGA | -43.93 |
| TGAT | -30.85 | TGTT | -37.55 | TGCT | -46.50 | TGGT | -44.86 |
| TGAC | -34.72 | TGTC | -38.66 | TGCC | -40.13 | TGGC | -46.86 |
| TGAG | -38.48 | TGTG | -39.23 | TGCG | -44.82 | TGGG | -45.86 |
| TGA) | -37.66 | TGT) | -37.42 | TGC) | -48.62 | TGG) | -48.54 |
| CGAA | -38.11 | CGTA | -40.46 | CGCA | -44.82 | CGGA | -46.88 |
| CGAT | -39.36 | CGTT | -36.06 | CGCT | -46.60 | CGGT | -42.53 |
| CGAC | -38.35 | CGTC | -34.53 | CGCC | -50.68 | CGGC | -46.15 |
| CGAG | -37.99 | CGTG | -38.48 | CGCG | -47.23 | CGGG | -40.74 |
| CGA) | -37.25 | CGT) | -37.48 | CGC) | -48.83 | CGG) | -52.17 |
| GGAA | -34.08 | GGTA | -36.35 | GGCA | -40.13 | GGGA | -49.11 |
| GGAT | -38.87 | GGTT | -37.46 | GGCT | -42.44 | GGGT | -48.51 |
| GGAC | -36.81 | GGTC | -39.47 | GGCC | -43.33 | GGGC | -44.84 |
| GGAG | -36.06 | GGTG | -37.06 | GGCG | -50.68 | GGGG | -46.01 |
| GGA) | -40.42 | GGT) | -27.69 | GGC) | -46.22 | GGG) | -46.38 |
| (GAA | -34.52 | (GTA | -42.25 | (GCA | -48.62 | (GGA | -48.06 |
| (GAT | -34.15 | (GTT | -38.47 | (GCT | -45.01 | (GGT | -46.29 |
| (GAC | -35.27 | (GTC | -38.91 | (GCC | -46.22 | (GGC | -52.00 |
| (GAG | -37.15 | (GTG | -40.11 | (GCG | -48.83 | (GGG | -51.30 |
| (GA) | -36.90 | (GT) | -37.47 | (GC) | -45.97 | (GG) | -46.47 |
| A)A | -17.00 | A)C | -10.00 | A(A | -15.22 | A(C | -25.50 |
| T)A | -12.12 | T)C | -16.58 | T(A | -10.36 | T(C | -24.42 |
| C)A | -22.64 | C)C | -19.64 | C(A | -12.12 | C(C | -22.95 |
| G)A | -23.34 | G)C | -24.04 | G(A | -11.37 | G(C | -26.12 |
| A)T | -15.98 | A)G | -13.31 | A(T | -16.63 | A(G | -26.93 |
| T)T | -15.89 | T)G | -16.00 | T(T | -12.82 | T(G | -23.08 |
| C)T | -22.57 | C)G | -24.68 | C(T | -15.68 | C(G | -21.80 |
| G)T | -24.36 | G)G | -24.70 | G(T | -19.87 | G(G | -24.83 |

**Table S5.** Sequence sets and hybridization thermodynamic parameters of 244 DNA duplexes.

| ID | sequenceA | sequenceB | $\Delta$G  (kcal/mol) | $\Delta$H  (kcal/mol) | $\Delta$S  (kcal/mol/K) |
| --- | --- | --- | --- | --- | --- |
| 1 | TTGCCCGT | ACGGGCAA | -8.30 | -38.08 | -0.09601 |
| 2 | GCTGCATCCA | TGGATGCAGC | -10.56 | -55.11 | -0.14364 |
| 3 | GAAGTGCCAATGGG | CCCATTGGCACTTC | -13.65 | -83.59 | -0.22550 |
| 4 | ACTGATTCT | AGAATCAGT | -8.55 | -46.11 | -0.12110 |
| 5 | CACGTACC | GGTACGTG | -8.90 | -48.11 | -0.12643 |
| 6 | ACTGCCTCCTA | TAGGAGGCAGT | -11.30 | -61.12 | -0.16063 |
| 7 | GCAGCA | TGCTGC | -7.44 | -29.15 | -0.07000 |
| 8 | CCGGAACATGGCA | TGCCATGTTCCGG | -13.37 | -76.92 | -0.20489 |
| 9 | TTGTCTA | TAGACAA | -6.43 | -32.78 | -0.08495 |
| 10 | AATTGTAGT | ACTACAATT | -7.91 | -48.41 | -0.13057 |
| 11 | GTGTTTAGCT | AGCTAAACAC | -9.42 | -52.63 | -0.13931 |
| 12 | TGCCT | AGGCA | -5.85 | -20.31 | -0.04662 |
| 13 | ACGAGATGGTA | TACCATCTCGT | -10.27 | -58.13 | -0.15432 |
| 14 | TATTATAT | ATATAATA | -6.63 | -46.93 | -0.12994 |
| 15 | AACGCTTAAGGG | CCCTTAAGCGTT | -11.27 | -66.34 | -0.17756 |
| 16 | ATTACCC | GGGTAAT | -6.90 | -34.19 | -0.08798 |
| 17 | GCTTCTCTTGGGT | ACCCAAGAGAAGC | -12.39 | -75.32 | -0.20291 |
| 18 | TGCGTGGGA | TCCCACGCA | -9.88 | -49.59 | -0.12804 |
| 19 | TATTCTCCCTCG | CGAGGGAGAATA | -11.38 | -68.34 | -0.18364 |
| 20 | TCCCGCT | AGCGGGA | -8.30 | -37.89 | -0.09541 |
| 21 | AACTACTCCCA | TGGGAGTAGTT | -9.92 | -53.34 | -0.14001 |
| 22 | GCGCAA | TTGCGC | -7.44 | -31.77 | -0.07845 |
| 23 | AATAGGACCTCC | GGAGGTCCTATT | -10.76 | -59.91 | -0.15846 |
| 24 | TCGGAT | ATCCGA | -6.65 | -30.94 | -0.07832 |
| 25 | GGTACGTCG | CGACGTACC | -9.67 | -50.92 | -0.13299 |
| 26 | AGGACTGG | CCAGTCCT | -8.74 | -41.68 | -0.10621 |
| 27 | CCGGACC | GGTCCGG | -8.53 | -40.50 | -0.10309 |
| 28 | AGACATCCAACTTG | CAAGTTGGATGTCT | -12.53 | -78.80 | -0.21367 |
| 29 | GGCTCAACTT | AAGTTGAGCC | -9.84 | -50.98 | -0.13263 |
| 30 | CTACGCGC | GCGCGTAG | -8.97 | -44.02 | -0.11301 |
| 31 | GCATCGTCC | GGACGATGC | -9.85 | -50.95 | -0.13251 |
| 32 | ATGCG | CGCAT | -6.34 | -27.49 | -0.06818 |
| 33 | GCGGTTGC | CAACCGC | -7.97 | -30.90 | -0.07394 |
| 34 | CCGAGTTC | GAACTCGTTA | -7.40 | -33.67 | -0.08470 |
| 35 | ATAAAGGACATA | TATGTCCT | -8.00 | -41.64 | -0.10845 |
| 36 | ACTTACAAGCCCGGG | CGGGCTTGTAAGT | -11.49 | -62.74 | -0.16525 |
| 37 | TACGCTGCAG | ATTACTGCAGCG | -9.99 | -48.60 | -0.12449 |
| 38 | CTGGAGTCAACACAAAAAA | ATAGTTTGTGTTGACTTTGG | -10.86 | -72.34 | -0.19822 |
| 39 | CGCGTAGCCCATAA | GGGCTA | -7.13 | -38.23 | -0.10027 |
| 40 | AGTAATTGAA | CAATTACTGAC | -7.36 | -43.95 | -0.11797 |
| 41 | ACTTCAGGCAAGTT | GATGCCTGATACT | -8.39 | -47.12 | -0.12486 |
| 42 | CTTGTACCATGGG | TGGTACAAGCAG | -8.69 | -55.30 | -0.15027 |
| 43 | TTACACTAGGTCTACA | TGTAGACCTAGT | -11.37 | -75.22 | -0.20588 |
| 44 | AGGTCAGCCGGTAT | CGGCTGACCT | -10.25 | -46.35 | -0.11640 |
| 45 | TTGGCATTGGA | CCAATGCCAAAAT | -10.00 | -53.81 | -0.14125 |
| 46 | GCAGTAATCAT | ATGATTA | -6.37 | -36.51 | -0.09719 |
| 47 | TTCACATTCGCC | GTCGCGAATGTG | -9.26 | -51.45 | -0.13602 |
| 48 | TCTCTATGCCTA | AGTTAGGCATA | -7.91 | -43.74 | -0.11554 |
| 49 | CCCCTGGGCAGAGCCTAA | GGGGCTCTGCCCAGGGAACT | -14.55 | -95.80 | -0.26197 |
| 50 | TACGTGCATGGTACAGAG | GTACCATGCACGC | -12.36 | -76.31 | -0.20620 |
| 51 | GGCTCGTGTTATTA | AATAACACGAGCCA | -11.23 | -60.15 | -0.15772 |
| 52 | GTGACTGCGACGCAC | GTGCGTCGCAGT | -12.90 | -70.18 | -0.18468 |
| 53 | TTTAGGGCCGAGAAT | TCTCGGCCCTTTT | -11.54 | -61.24 | -0.16023 |
| 54 | CCGACGTCCAC | ATGGACGCAT | -8.88 | -55.69 | -0.15093 |
| 55 | AGGTTTTCA | AACCTTA | -5.60 | -25.42 | -0.06389 |
| 56 | TCCATTGATAAC | TTATCAATGGA | -9.01 | -52.86 | -0.14137 |
| 57 | GCCAGTTTGACGCAGTTAGG | GTAACTGCGTCAAACAC | -13.10 | -81.33 | -0.21999 |
| 58 | TGGGATGCTA | GTTAGCATCCC | -9.45 | -49.60 | -0.12945 |
| 59 | ATGCTGGCAATCTA | TTGCCA | -8.02 | -39.25 | -0.10070 |
| 60 | AAGGTTACATGCG | TTTCGCATGTAACC | -12.05 | -74.86 | -0.20252 |
| 61 | TGTACTATGTTGAG | CTCAACATAGCAAC | -9.66 | -63.37 | -0.17317 |
| 62 | CCCCCGGGCGCCC | GTTTGGCGCCCGG | -12.44 | -66.58 | -0.17455 |
| 63 | CTTCCTCATCTGATTGAACAC | TCAATCAGATGAGG | -13.57 | -85.33 | -0.23137 |
| 64 | GTAAGCGTACAAC | GTTGTACGCCGT | -10.11 | -60.61 | -0.16281 |
| 65 | CACACCCAAATCT | TGTCTTGGGTGTCGGA | -9.11 | -57.56 | -0.15621 |
| 66 | TAGATGAAACATTC | ATGTTTCATCTAAAAG | -9.83 | -64.19 | -0.17526 |
| 67 | ACGGTGTTACTTAT | GACTAAGTAACATAGC | -7.77 | -47.28 | -0.12738 |
| 68 | TAAAATCGGAATATTCG | CCAGTATTCCGATTTCCCG | -9.67 | -55.61 | -0.14813 |
| 69 | AAATTCTTAATCGAC | CATACGATTAAGAACG | -8.66 | -55.03 | -0.14952 |
| 70 | ATGGCCCATA | ATGGGTGCG | -7.22 | -33.69 | -0.08534 |
| 71 | TGATACCGTGG | GGTATCATCCA | -6.47 | -37.27 | -0.09930 |
| 72 | CCAACGAGTGTGG | TCCGCACACTCGCC | -9.40 | -50.87 | -0.13371 |
| 73 | TATTGTCCTAGTT | GGATTAGGACAGGAT | -7.17 | -38.26 | -0.10025 |
| 74 | GGCATCACTTTCACGT | GTGAAAGTGATGCGACG | -11.38 | -60.76 | -0.15921 |
| 75 | TACCAAGGAG | TTGGTATCCG | -5.91 | -31.13 | -0.08133 |
| 76 | GTAGATATCACCGAGGGA | TCCCTCGGTGATATTT | -12.62 | -79.48 | -0.21556 |
| 77 | TTTCGGCTACCA | AGCCGAATCAA | -7.95 | -36.97 | -0.09358 |
| 78 | TACCGCGTCGCTTGTA | CAGCGACGCGGT | -12.64 | -76.14 | -0.20473 |
| 79 | ACCAGCTACGCCT | AGGCGTAGCTCCGT | -10.31 | -48.37 | -0.12273 |
| 80 | AGTCCACATTGCTCTCA | GAGCAATGTGGACTGACA | -12.47 | -70.68 | -0.18767 |
| 81 | ATTCTATAAGATACGTGCTTGA | CTTGCACGTATCTTATA | -11.16 | -66.92 | -0.17977 |
| 82 | TGGTTTAAGTAGATTACCGG | GGTAATCTACTTAA | -10.73 | -71.48 | -0.19586 |
| 83 | CCCACGATCTC | CCTGGAGATCGTGG | -9.80 | -50.51 | -0.13126 |
| 84 | GTCGGCTTGATTGAACTCGA | GAGTGTTCAATCAAGCCGT | -13.20 | -76.83 | -0.20515 |
| 85 | AGACACAACGCAACCG | GCTAGGTTGCGTTGTGTC | -12.89 | -73.05 | -0.19397 |
| 86 | CTATTATTACCGCAGAA | GCGGTAATAAT | -10.60 | -70.07 | -0.19173 |
| 87 | CGCAGCGCCGAGGTC | AATGGACCTCGGCGCTGC | -13.77 | -68.60 | -0.17680 |
| 88 | TAGGCCGGATTAGTGAGCTT | TCACTAATCCGGCCAT | -12.65 | -66.44 | -0.17343 |
| 89 | TGGCCCTGGTCTAGTCCTCA | TCTTGACTAGACCAGGG | -11.15 | -49.12 | -0.12243 |
| 90 | GGCAACGCACTATCTTC | AGATAGTGCGTTGGGGT | -11.56 | -65.66 | -0.17442 |
| 91 | TGCATGAT | GTCATCAT | -5.87 | -28.51 | -0.07299 |
| 92 | TACGTGCGT | ACGCA | -6.57 | -31.03 | -0.07888 |
| 93 | AATATTATCAAATTAGATTG | CTAATTTGATAATACGAG | -10.37 | -70.83 | -0.19494 |
| 94 | AGCGGCTATACTT | CGAAGTATAGCCG | -10.25 | -54.64 | -0.14313 |
| 95 | TCGACATAGCTCCCC | GGGAGCTATGTCGA | -12.50 | -71.49 | -0.19021 |
| 96 | AGAAAACAGTAGTAGAAG | CTTCTACTACTGTTAGGA | -12.41 | -87.43 | -0.24189 |
| 97 | CGTTGACTTTCGCCTG | CAGGCGAAAGTCAG | -12.86 | -75.74 | -0.20275 |
| 98 | GTAGTAATATAATCG | TATATTACTAC | -8.40 | -66.84 | -0.18844 |
| 99 | GGTGGCGATCATAGGG | CTATGATCGCCACC | -12.81 | -72.22 | -0.19156 |
| 100 | TGACTCTT | AGTCAG | -5.59 | -22.22 | -0.05363 |
| 101 | GGTCGGCGTTAAT | CGATTAACGCCGA | -10.98 | -57.40 | -0.14968 |
| 102 | GAGACCAAGTTGTCTGGCTGAA | GCCAGACAACTTGGATGA | -14.70 | -97.11 | -0.26571 |
| 103 | AGGCAATAGATCG | GATCTATTGCAAA | -9.79 | -58.68 | -0.15763 |
| 104 | TTTAACGGTCGGTATGT | ACATACCGACCGTT | -12.74 | -71.53 | -0.18956 |
| 105 | ATCTTCACAACACGGAT | ATCCGTGTTGTGAA | -12.60 | -77.73 | -0.20998 |
| 106 | TTACGGGCAACGGGTCCGT | GACCCGTTGCCCG | -13.57 | -72.67 | -0.19055 |
| 107 | CAGGCCACGG | TGGCCTG | -7.68 | -29.61 | -0.07070 |
| 108 | GGGTCCGC | AAGGCGGACC | -8.65 | -34.29 | -0.08268 |
| 109 | TAGTGCTTTCCTATGTT | AACATAGGAAAGCA | -12.16 | -76.49 | -0.20741 |
| 110 | TCGATCTTC | GCCGATCGC | -6.93 | -36.35 | -0.09485 |
| 111 | TACCCGACAAATGATAGGCAA | AACTATCATTTGTCGGCAA | -13.70 | -91.74 | -0.25161 |
| 112 | TTCGTAGGTAAGCA | TCTTGCTTACCTAC | -10.42 | -60.85 | -0.16259 |
| 113 | CAAGATCCCAGA | GGATC | -5.85 | -25.90 | -0.06464 |
| 114 | CCTTGTGAGGCGGT | ACCGCCTCACAA | -12.12 | -63.85 | -0.16680 |
| 115 | AACAATATT | ATATTGTTAG | -6.96 | -43.36 | -0.11735 |
| 116 | ACACTGGATATA | TCCAGTGTAAGA | -7.96 | -41.38 | -0.10775 |
| 117 | AGCAGTACCAACTCA | TTTCAGTTGGTACTGCGT | -10.21 | -42.20 | -0.10315 |
| 118 | AGACTTACACGGGTCC | GACCCGTGTAAGTC | -13.15 | -77.87 | -0.20866 |
| 119 | TATTCATTTCCT | AATGAATAGAAT | -7.00 | -42.00 | -0.11285 |
| 120 | GTGATGAGTCTGTCTGCGG | AGACAGACTCATCTCCC | -11.05 | -61.60 | -0.16300 |
| 121 | CTATATTGGA | CCATCCAATA | -7.24 | -35.86 | -0.09229 |
| 122 | GAGTAGGCGGCTG | CAGCCGCCTACA | -12.32 | -62.93 | -0.16318 |
| 123 | TTGCGCCCAAAGA | TCTTTGGGCGC | -11.13 | -54.65 | -0.14033 |
| 124 | TGCCATCTAGC | CGATAGATGG | -7.85 | -48.07 | -0.12969 |
| 125 | GGTCCGTTCGG | CCGAACGGG | -9.24 | -41.99 | -0.10560 |
| 126 | AACTATGGCGAACG | CAACGCCATA | -7.97 | -35.55 | -0.08894 |
| 127 | GGCCGCCATGC | GCATGGCGG | -10.41 | -49.57 | -0.12626 |
| 128 | TTATCTCGGGAT | TCCCGAG | -7.71 | -32.12 | -0.07871 |
| 129 | AAGCAAAAATTA | TAATTTTTGTG | -8.33 | -51.19 | -0.13820 |
| 130 | AGAGAAGGCCAGTC | AATGGCCTTCTCG | -11.03 | -58.93 | -0.15443 |
| 131 | TAGTCCTCTATTTAAGTGTG | CTTAAATAGAGG | -11.00 | -84.78 | -0.23789 |
| 132 | CGCGAAAAAATGACGCTA | GCGTCATTTTTTCG | -12.89 | -80.80 | -0.21896 |
| 133 | CAACAACCTTTACCTGC | TGGTAAAGGTTGTT | -11.40 | -69.63 | -0.18776 |
| 134 | ATGAATCCCACAGTCA | CTGTGGGATAA | -9.26 | -50.65 | -0.13345 |
| 135 | ACTAAATGCAACACCCGT | GGTGTTGCATTTAG | -12.75 | -86.53 | -0.23790 |
| 136 | TCGTCGGGGGGG | AACCCCCGAGATG | -8.70 | -42.21 | -0.10804 |
| 137 | AGCAGCCC | AGGCTGA | -7.39 | -27.92 | -0.06620 |
| 138 | AGCGAATCACCCAATG | GGGTGATTA | -8.27 | -42.10 | -0.10908 |
| 139 | GTGGAATTTTTTA | AAAAAATTCCACAAG | -10.42 | -62.68 | -0.16849 |
| 140 | TACGAT | ATCGTCCTT | -5.88 | -25.98 | -0.06480 |
| 141 | AAACCGACTCCCAA | GAGTCGGTGCAT | -9.42 | -49.32 | -0.12865 |
| 142 | TTTTTCCGAGTAGC | ACTCGGAC | -7.48 | -34.72 | -0.08783 |
| 143 | CTTCTGGTTAGGCAAGTA | TCGTTGCCTAACCAGAAC | -11.86 | -67.50 | -0.17940 |
| 144 | TCTAATCCTGG | CCAGGATAGTA | -7.79 | -45.58 | -0.12186 |
| 145 | AAGCAGCTGAGC | GTCAGCTGCT | -9.67 | -45.61 | -0.11587 |
| 146 | TATCTAAGGTCG | CCTTAGATA | -7.93 | -51.45 | -0.14031 |
| 147 | GTCCATCCAGTATCCT | CTACTGGAT | -7.53 | -38.75 | -0.10067 |
| 148 | GTTTCTCCTAGGCGG | CTAGGAGAAAC | -9.94 | -59.58 | -0.16006 |
| 149 | TCTAATCCGTGACGTTA | TCGTCACGGATT | -10.84 | -58.78 | -0.15458 |
| 150 | GTCCCCGGTCCTCTCTAG | AGAGGACCGGGGATT | -11.86 | -58.31 | -0.14977 |
| 151 | TGAAGGGACAA | GCGTTGTCCCTT | -8.66 | -45.69 | -0.11939 |
| 152 | GCCCAGAACATCATAAG | AATGATGTTCT | -9.25 | -58.17 | -0.15773 |
| 153 | ATACCCCTCCTA | TAGGAGGGGTA | -10.59 | -49.18 | -0.12441 |
| 154 | CAGAGTACA | TACTCTGACAC | -6.98 | -36.73 | -0.09592 |
| 155 | AATCCTGCGCGCTA | TAGCGCGCAGGAT | -13.38 | -66.00 | -0.16965 |
| 156 | CACACTCAGACC | TCTGAGTGTGGGT | -9.71 | -55.44 | -0.14745 |
| 157 | GAGCGTATTATCTAC | GATAATACGCTC | -10.24 | -64.61 | -0.17531 |
| 158 | TAGACGTAATGGCAT | CATTACGTCT | -9.41 | -59.28 | -0.16078 |
| 159 | TCCATTTATACAATTCATT | GGGAATTGTATAAAT | -9.69 | -66.33 | -0.18261 |
| 160 | TTTCCGAAGA | CGGAAA | -5.75 | -28.84 | -0.07446 |
| 161 | CGTACGATTATTT | AAATAATCGTA | -9.58 | -63.16 | -0.17277 |
| 162 | AACGGTGATACA | TGTATCAC | -8.16 | -49.61 | -0.13366 |
| 163 | GTGCACGGATTT | AAATCCGTGCT | -10.21 | -54.09 | -0.14149 |
| 164 | GCAGCTCTACGTATT | ACGTAGAGCTG | -11.68 | -68.15 | -0.18208 |
| 165 | CCTTGGACAAT | ATTGTCCAACTC | -8.13 | -43.35 | -0.11356 |
| 166 | GGAGAACCTAC | CGCCTAGGTTGC | -6.77 | -33.37 | -0.08576 |
| 167 | CCTGACCGCTGAAATTCCC | ATTTCAGCGGTCAA | -11.58 | -61.41 | -0.16068 |
| 168 | GGAGGTAGCAGGTTAGGTT | TAACCTGCTACCT | -11.39 | -62.49 | -0.16476 |
| 169 | TCACATGCTGA | ACAATCAGCATGT | -8.85 | -44.61 | -0.11529 |
| 170 | GAAGGAGTATTATCGCAGTGT | CTGCGATAATACTC | -12.66 | -79.67 | -0.21605 |
| 171 | CCATGTAGGGAGGTG | TCCCTACATG | -9.52 | -53.81 | -0.14280 |
| 172 | AGTCCTGTCTTGAACCGC | TTCAAGACAGGACTTTTA | -11.80 | -72.51 | -0.19573 |
| 173 | GTTTAGTTGGC | CTGCGCCAACTA | -8.12 | -41.20 | -0.10666 |
| 174 | ACTACAC | TGTAGTACT | -6.66 | -29.21 | -0.07271 |
| 175 | GCATCCTTGTAC | GGTCGTACAAGG | -7.95 | -40.62 | -0.10533 |
| 176 | CACCGATTCATGAGCA | TGCTCATGAATCGGCT | -13.76 | -84.06 | -0.22667 |
| 177 | CTTTTGGCTAGCATCTT | TGCTAGCCA | -9.46 | -47.14 | -0.12149 |
| 178 | CAAAGCAACAAAGTGG | CCACTTTGTTGCTCT | -12.83 | -77.78 | -0.20943 |
| 179 | GAAGCTTCACGT | GTGAAGCTTC | -9.75 | -51.22 | -0.13372 |
| 180 | TGTTGACGC | CTGCGTCAA | -8.37 | -38.68 | -0.09772 |
| 181 | ATCGTATTGGG | CCCAATATAA | -7.10 | -37.46 | -0.09789 |
| 182 | GAACGAGCCTTAC | CTCTGTAAGGCTCG | -11.05 | -63.44 | -0.16893 |
| 183 | GCAACTCTGTT | AAGCAACAGAGTT | -8.48 | -44.90 | -0.11742 |
| 184 | TCTTCCCATAGCCGACCTT | TCGGCTATGGGAA | -11.17 | -58.65 | -0.15308 |
| 185 | GATACTG | CAAGCAGTA | -5.90 | -27.47 | -0.06955 |
| 186 | GGCAACCATACCTCACTG | CGGGTGAGGTATGGTTGTTG | -13.86 | -88.31 | -0.24006 |
| 187 | TAAGCGGGCTT | GCCCGCTT | -10.01 | -39.80 | -0.09606 |
| 188 | ACCCTGTTTTCCATCT | TGGAAAACAGGGT | -12.25 | -82.43 | -0.22628 |
| 189 | TAAGTGCTT | AAGCACTT | -8.23 | -40.31 | -0.10343 |
| 190 | GCCTATTAACACCTA | GGTGTTAATAC | -8.48 | -54.78 | -0.14928 |
| 191 | CTAGTGCGTACAAA | TGTACGCACTAG | -11.61 | -63.72 | -0.16800 |
| 192 | CAGAAGACA | TCTTCTG | -6.90 | -34.79 | -0.08994 |
| 193 | GGACGAGAAGTACG | GTACTTCTCGTCC | -11.70 | -68.57 | -0.18337 |
| 194 | CAGGTCGACTAAGATAC | GTATCTTAGTCGA | -11.38 | -75.15 | -0.20560 |
| 195 | CAGAGGAGTATTGTC | GACAATACTCCAC | -11.12 | -72.11 | -0.19664 |
| 196 | AGCGCACAGTAAATGC | CATTTACTGTG | -9.74 | -63.99 | -0.17493 |
| 197 | ACGTAT | ATACGT | -6.40 | -30.21 | -0.07678 |
| 198 | GTATGTCGTTC | GAACGACATAC | -10.48 | -62.72 | -0.16843 |
| 199 | ACTCTCTGGG | CCCAGAGAGT | -9.74 | -54.10 | -0.14303 |
| 200 | CTTGCAACCTCA | TGAGGTTGCAAG | -11.67 | -65.94 | -0.17497 |
| 201 | CGATT | AATCG | -5.58 | -25.89 | -0.06547 |
| 202 | GATGGA | TCCATC | -6.40 | -31.95 | -0.08239 |
| 203 | CAATAAGTGGT | ACCACTTATTG | -9.92 | -62.78 | -0.17043 |
| 204 | CACGC | GCGTG | -6.68 | -27.73 | -0.06788 |
| 205 | TTTTGCCCACGTT | AACGTGGGCAAAA | -12.77 | -72.44 | -0.19240 |
| 206 | TCGTGAT | ATCACGA | -7.34 | -34.46 | -0.08743 |
| 207 | TAGACCGAGTT | AACTCGGTCTA | -10.25 | -55.34 | -0.14537 |
| 208 | CGGATATC | GATATCCG | -8.08 | -40.77 | -0.10541 |
| 209 | CTCCCACCTCCACC | GGTGGAGGTGGGAG | -14.26 | -85.74 | -0.23046 |
| 210 | GGTGGTGTTGAGG | CCTCAACACCACC | -12.19 | -71.41 | -0.19095 |
| 211 | GTGGCAAGTGTAC | GTACACTTGCCAC | -12.10 | -69.51 | -0.18512 |
| 212 | CATCGGTTACTTTT | AAAAGTAACCGATG | -12.54 | -80.27 | -0.21839 |
| 213 | ACAATCGTTTA | TAAACGATTGT | -9.70 | -58.11 | -0.15609 |
| 214 | GTCCCGGAGA | TCTCCGGGAC | -10.30 | -53.42 | -0.13903 |
| 215 | AATTCATC | GATGAATT | -7.12 | -41.61 | -0.11120 |
| 216 | TGCAC | GTGCA | -6.16 | -26.24 | -0.06474 |
| 217 | AGCTCCTGACTA | TAGTCAGGAGCT | -10.83 | -58.12 | -0.15246 |
| 218 | GTGTGA | TCACAC | -6.78 | -30.53 | -0.07657 |
| 219 | CCGTGTTCA | TGAACACGG | -9.58 | -51.63 | -0.13557 |
| 220 | TATCCGGCTTTT | AAAAGCCGGATA | -10.86 | -57.77 | -0.15124 |
| 221 | TTTCAGGAAATGGC | ATGCCATTTCCT | -10.62 | -59.38 | -0.15721 |
| 222 | CCGCGA | CGGTCGCG | -7.00 | -25.67 | -0.06021 |
| 223 | AAATTCGGTTCC | CTTCCCGAATT | -7.38 | -36.76 | -0.09474 |
| 224 | GTACCCCTGCTACGCGACAT | TCGCGTAGCAGGGGGAT | -13.61 | -76.64 | -0.20324 |
| 225 | TGCAGTCAA | TGACTG | -6.70 | -26.36 | -0.06339 |
| 226 | TTTCTGTCACGT | TGACA | -6.71 | -38.13 | -0.10131 |
| 227 | TTCGAGCCTATCCAT | ATGGATAGGCT | -10.60 | -59.90 | -0.15894 |
| 228 | GCTACTAATCGT | ATTAGTAGCGAT | -8.31 | -49.25 | -0.13201 |
| 229 | GCAGCTAAGGTA | TCTTAGCTCAAT | -7.45 | -40.34 | -0.10603 |
| 230 | ACGTACATGGTA | ACCATGTACCAA | -8.60 | -43.81 | -0.11352 |
| 231 | AATGCTCTGGTG | CACCAGAG | -8.69 | -46.47 | -0.12182 |
| 232 | TCATGGTCTGTAGG | CCTACAGACCATCG | -11.66 | -72.84 | -0.19726 |
| 233 | GTGAGTCCCGATTC | GAATCGGGACTCA | -12.49 | -75.39 | -0.20281 |
| 234 | AGCAGAAACCGGACTG | TCCGGTTTCTGCT | -12.39 | -70.60 | -0.18768 |
| 235 | TATCCCCGTACGAG | CTATCGTACGGGGAT | -12.23 | -70.89 | -0.18915 |
| 236 | CCTCAGGC | GCCTGAGAACA | -8.05 | -39.02 | -0.09985 |
| 237 | TGAATTCGTA | TCGAATTCTGG | -8.40 | -44.64 | -0.11684 |
| 238 | TAACAGCA | GCTGTTATAC | -7.46 | -36.65 | -0.09410 |
| 239 | CTATGTTTAGTGTAA | TTACACTAAACTTCT | -9.50 | -62.15 | -0.16977 |
| 240 | GTCTGTGCCATCCGTA | GGATGGCACAGACGG | -12.39 | -75.27 | -0.20273 |
| 241 | CTTCAGCGGAAATTCT | AATTTCCGCTCTT | -9.97 | -60.40 | -0.16261 |
| 242 | TGGATGCCTGGCGC | CCAGGCATCCAAA | -11.27 | -62.98 | -0.16672 |
| 243 | CACAGTGCGTCGTCTAA | TAGACGACGCAC | -11.87 | -61.49 | -0.15998 |
| 244 | CCCTAAGGCCTC | ACTGAGGCCTTAG | -10.39 | -55.89 | -0.14671 |

**Table S6.** Sequence sets of 229 DNA strand displacement.

| ID | sequence |
| --- | --- |
| 1A | TTCGGCAATCAACGATTTACGTTTTTTATCTACTCT |
| 1B | GCACGGCGTACGGACTCACGGTGTTTCGGCAATCAACGATTTACGT |
| 1C | AGAGTAGATTTTTTTACGTAAATCGTTGATTGCCGAATGGTCGGTGCTCGCAGGCTCGGCA |
| 2A | ATGTAGTCGCTGCACAGCGATTTTTGAGCCGTACAGT |
| 2B | GCACGGCGTACGGACTCACGGTGTATGTAGTCGCTGCACAGCGATT |
| 2C | ACTGTACGGCTCATTTTTTTTTAATCGCTGTGCAGCGACTACATTGGTCGGTGCTCGCAGGCTCGGCA |
| 3A | ATAGCAAGCGTATAAAATGGGTTTCACGTACGG |
| 3B | GCACGGCGTACGGACTCACGGTGTATAGCAAGCGTATAAAATGGGT |
| 3C | CCGTACGTGATTTTTTTTTACCCATTTTATACGCTTGCTATTGGTCGGTGCTCGCAGGCTCGGCA |
| 4A | GGGGTAATACATAAGATTGCTTTTTGTGT |
| 4B | GCACGGCGTACGGACTCACGGTGTGGGGTAATACATAAGATTGCTT |
| 4C | ACACAAGCAATCTTATGTATTACCCCTGGTCGGTGCTCGCAGGCTCGGCA |
| 5A | TGGTTAGAGTCTGCCCAGACAGAACAATTTTA |
| 5B | GCACGGCGTACGGACTCACGGTGTTGGTTAGAGTCTGCCCAGACAG |
| 5C | TAAAATTGTTTTTCTGTCTGGGCAGACTCTAACCATGGTCGGTGCTCGCAGGCTCGGCA |
| 6A | ACTAGCTTGCCTGGTACACTGGTTTTTAACTACATCAAC |
| 6B | GCACGGCGTACGGACTCACGGTGTACTAGCTTGCCTGGTACACTGG |
| 6C | GTTGATGTAGTTTTTCCAGTGTACCAGGCAAGCTAGTTGGTCGGTGCTCGCAGGCTCGGCA |
| 7A | TTCAGACTATCGCATTTCAACAATAAATCTAAGT |
| 7B | GCACGGCGTACGGACTCACGGTGTTTCAGACTATCGCATTTCAACA |
| 7C | ACTTAGATTTATTGTTGAAATGCGATAGTCTGAATGGTCGGTGCTCGCAGGCTCGGCA |
| 8A | AGATTTGGTAGGCCACAAGTGGTTGGAGCCCTC |
| 8B | GCACGGCGTACGGACTCACGGTGTAGATTTGGTAGGCCACAAGTGG |
| 8C | GAGGGCTCCTTTTTTTTCCACTTGTGGCCTACCAAATCTTGGTCGGTGCTCGCAGGCTCGGCA |
| 9A | CACAGACAAACTCAGAATGCACTTTTGTTCTGT |
| 9B | GCACGGCGTACGGACTCACGGTGTCACAGACAAACTCAGAATGCAC |
| 9C | ACAGAACGTGCATTCTGAGTTTGTCTGTGTGGTCGGTGCTCGCAGGCTCGGCA |
| 10A | ACATTTTAAATCATAGTCAACATTTTTTTTAGCGCTGTG |
| 10B | GCACGGCGTACGGACTCACGGTGTACATTTTAAATCATAGTCAACA |
| 10C | CACAGCGCTTTTTTTTTGTTGACTATGATTTAAAATGTTGGTCGGTGCTCGCAGGCTCGGCA |
| 11A | CCGCGGACGGCGCCTACGCCGGAGGCT |
| 11B | GCACGGCGTACGGACTCACGGTGTCCGCGGACGGCGCCTACGCCGG |
| 11C | AGCCTCCGGCGTAGGCGCCGTCCGCGGTGGTCGGTGCTCGCAGGCTCGGCA |
| 12A | ATGTCCCCCGAGGCGCGGCGCGTTTTTTTTTTTTCACGGAATG |
| 12B | GCACGGCGTACGGACTCACGGTGTATGTCCCCCGAGGCGCGGCGCG |
| 12C | CATTCCGTGAAATTCGCGCCGCGCCTCGGGGGACATTGGTCGGTGCTCGCAGGCTCGGCA |
| 13A | TCCGGATGTCCTAATGGTTTTATTTTTTTGACTATGAA |
| 13B | GCACGGCGTACGGACTCACGGTGTTCCGGATGTCCTAATGGTTTTA |
| 13C | TTCATAGTCTTAAAACCATTAGGACATCCGGATGGTCGGTGCTCGCAGGCTCGGCA |
| 14A | GAGGTGAGGGCTCTCCACTTCGTTTTTTTTTTTCGGGAGAAAA |
| 14B | GCACGGCGTACGGACTCACGGTGTGAGGTGAGGGCTCTCCACTTCG |
| 14C | TTTTCTCCCGATTTTTTCGAAGTGGAGAGCCCTCACCTCTGGTCGGTGCTCGCAGGCTCGGCA |
| 15A | GTGACTGAGTCGAACATAGCGATTTTTTTGTTAATACTCT |
| 15B | GCACGGCGTACGGACTCACGGTGTGTGACTGAGTCGAACATAGCGA |
| 15C | AGAGTATTAACTTTTTTTTTTTCGCTATGTTCGACTCAGTCACTGGTCGGTGCTCGCAGGCTCGGCA |
| 16A | TGGAACGAAGTTCCATAAGTCATTTTTTAGACGTGCGACT |
| 16B | GCACGGCGTACGGACTCACGGTGTTGGAACGAAGTTCCATAAGTCA |
| 16C | AGTCGCACGTCTTGACTTATGGAACTTCGTTCCATGGTCGGTGCTCGCAGGCTCGGCA |
| 17A | TCACAGCGTTATATCATCTAACCTGCGCCGATTGC |
| 17B | GCACGGCGTACGGACTCACGGTGTTCACAGCGTTATATCATCTAAC |
| 17C | GCAATCGGCGCAGTTTTTTTTTTGTTAGATGATATAACGCTGTGATGGTCGGTGCTCGCAGGCTCGGCA |
| 18A | CTATCTTCGACTTTAAACCTTCGAAGTCTT |
| 18B | GCACGGCGTACGGACTCACGGTGTCTATCTTCGACTTTAAACCTTC |
| 18C | AAGACTTCTTTTTGAAGGTTTAAAGTCGAAGATAGTGGTCGGTGCTCGCAGGCTCGGCA |
| 19A | TCTTATTAGGACAAAAGGGATTGCAAAGGATAT |
| 19B | GCACGGCGTACGGACTCACGGTGTTCTTATTAGGACAAAAGGGATT |
| 19C | ATATCCTTTGCTTTAATCCCTTTTGTCCTAATAAGATGGTCGGTGCTCGCAGGCTCGGCA |
| 20A | TCGCGCACAATTCGTAACACTCTTTTTTTCGCTCGGCCGTA |
| 20B | GCACGGCGTACGGACTCACGGTGTTCGCGCACAATTCGTAACACTC |
| 20C | TACGGCCGAGCGTTTTTTTTTGAGTGTTACGAATTGTGCGCGATGGTCGGTGCTCGCAGGCTCGGCA |
| 21A | CACACAGTGCCAATCCGAAACCTCGTA |
| 21B | GCACGGCGTACGGACTCACGGTGTCACACAGTGCCAATCCGAAACC |
| 21C | TACGTGGTTTCGGATTGGCACTGTGTGTGGTCGGTGCTCGCAGGCTCGGCA |
| 22A | TACTCTGTCCCCCGATTACTTATGCGCTTCCTG |
| 22B | GCACGGCGTACGGACTCACGGTGTTACTCTGTCCCCCGATTACTTA |
| 22C | CAGGAAGCGCATAAGTAATCGGGGGACAGAGTATGGTCGGTGCTCGCAGGCTCGGCA |
| 23A | CTGTCCCCTTCACTCAAGATCATTTTTTTTACAATCCTC |
| 23B | GCACGGCGTACGGACTCACGGTGTCTGTCCCCTTCACTCAAGATCA |
| 23C | GAGGATTGTTTGATCTTGAGTGAAGGGGACAGTGGTCGGTGCTCGCAGGCTCGGCA |
| 24A | CGAACCAATTTGAATAGTAGCATTCTGTCCGC |
| 24B | GCACGGCGTACGGACTCACGGTGTCGAACCAATTTGAATAGTAGCA |
| 24C | GCGGACAGTGCTACTATTCAAATTGGTTCGTGGTCGGTGCTCGCAGGCTCGGCA |
| 25A | AGGAATAGGAGTCAGCCTTCTTTACTG |
| 25B | GCACGGCGTACGGACTCACGGTGTAGGAATAGGAGTCAGCCTTCTT |
| 25C | CAGTATTAAGAAGGCTGACTCCTATTCCTTGGTCGGTGCTCGCAGGCTCGGCA |
| 26A | GGGACGTCTCACGATATCCTATTTTTTTTCATGTT |
| 26B | GCACGGCGTACGGACTCACGGTGTGGGACGTCTCACGATATCCTAT |
| 26C | AACATGAAAATTTTTTTATAGGATATCGTGAGACGTCCCTGGTCGGTGCTCGCAGGCTCGGCA |
| 27A | CTAGAGGTCCCTCGTTTATGCCTTTTTTGATGTCGTA |
| 27B | GCACGGCGTACGGACTCACGGTGTCTAGAGGTCCCTCGTTTATGCC |
| 27C | TACGACATCATTTTTGGCATAAACGAGGGACCTCTAGTGGTCGGTGCTCGCAGGCTCGGCA |
| 28A | TGATACGCCGGCTGATACGCCCTTTAGCGCTGTACTT |
| 28B | GCACGGCGTACGGACTCACGGTGTTGATACGCCGGCTGATACGCCC |
| 28C | AAGTACAGCGCTTTTTTTGGGCGTATCAGCCGGCGTATCATGGTCGGTGCTCGCAGGCTCGGCA |
| 29A | CGTAGGCATATCAGGCATCGGATTTCCATATAT |
| 29B | GCACGGCGTACGGACTCACGGTGTCGTAGGCATATCAGGCATCGGA |
| 29C | ATATATGGTTCCGATGCCTGATATGCCTACGTGGTCGGTGCTCGCAGGCTCGGCA |
| 30A | TAGAATCTAACGTTTCATTCGCCACTAT |
| 30B | GCACGGCGTACGGACTCACGGTGTTAGAATCTAACGTTTCATTCGC |
| 30C | ATAGTGGCGAATGAAACGTTAGATTCTATGGTCGGTGCTCGCAGGCTCGGCA |
| 31A | CCACCCTCTGTCGAAAGGTCTTTTTTCGGCAGCGGT |
| 31B | GCACGGCGTACGGACTCACGGTGTCCACCCTCTGTCGAAAGGTCTT |
| 31C | ACCGCTGCCGTAAGACCTTTCGACAGAGGGTGGTGGTCGGTGCTCGCAGGCTCGGCA |
| 32A | GCCAGCTACGTCTCCATAACAGTTTTTTTCTTGACTAAG |
| 32B | GCACGGCGTACGGACTCACGGTGTGCCAGCTACGTCTCCATAACAG |
| 32C | CTTAGTCAAGTTTTTCTGTTATGGAGACGTAGCTGGCTGGTCGGTGCTCGCAGGCTCGGCA |
| 33A | GAACGTTACTCTTATAAGTTTGTTGGATTCGACAC |
| 33B | GCACGGCGTACGGACTCACGGTGTGAACGTTACTCTTATAAGTTTG |
| 33C | GTGTCGAATCCTTCAAACTTATAAGAGTAACGTTCTGGTCGGTGCTCGCAGGCTCGGCA |
| 34A | CGGTAGGACCAATCATCAGCGGTTTTTACCAAAG |
| 34B | GCACGGCGTACGGACTCACGGTGTCGGTAGGACCAATCATCAGCGG |
| 34C | CTTTGGTTTTTTCCGCTGATGATTGGTCCTACCGTGGTCGGTGCTCGCAGGCTCGGCA |
| 35A | GGATTAGTCCTAAGATCGCAACTTTTTTTAGGCACTATACCGC |
| 35B | GCACGGCGTACGGACTCACGGTGTGGATTAGTCCTAAGATCGCAAC |
| 35C | GCGGTATAGTGCCTTTTTTTTTTGTTGCGATCTTAGGACTAATCCTGGTCGGTGCTCGCAGGCTCGGCA |
| 36A | GTTCAAGCACGCTCCCCAGCGGTTTTCAGTCTTGATCAC |
| 36B | GCACGGCGTACGGACTCACGGTGTGTTCAAGCACGCTCCCCAGCGG |
| 36C | GTGATCAAGACTGTTTTTTTTCCGCTGGGGAGCGTGCTTGAACTGGTCGGTGCTCGCAGGCTCGGCA |
| 37A | AAGGGACTCGTTATCCCATGATTGGAATAAGAC |
| 37B | GCACGGCGTACGGACTCACGGTGTAAGGGACTCGTTATCCCATGAT |
| 37C | GTCTTATTCCATTTATCATGGGATAACGAGTCCCTTTGGTCGGTGCTCGCAGGCTCGGCA |
| 38A | AGTCGCACATCCCTCTGCTAACTTTTTTTTGAGAATTCA |
| 38B | GCACGGCGTACGGACTCACGGTGTAGTCGCACATCCCTCTGCTAAC |
| 38C | TGAATTCTCTTTTTTGTTAGCAGAGGGATGTGCGACTTGGTCGGTGCTCGCAGGCTCGGCA |
| 39A | GACGCGGCTTTAATACTGTGACTTTTTTTTTTGACCCAGGCACGT |
| 39B | GCACGGCGTACGGACTCACGGTGTGACGCGGCTTTAATACTGTGAC |
| 39C | ACGTGCCTGGGTCATGTCACAGTATTAAAGCCGCGTCTGGTCGGTGCTCGCAGGCTCGGCA |
| 40A | CGGGAAGCACGGTCTCATGTAATTTTGAGCCCGAT |
| 40B | GCACGGCGTACGGACTCACGGTGTCGGGAAGCACGGTCTCATGTAA |
| 40C | ATCGGGCTCTTTTTTACATGAGACCGTGCTTCCCGTGGTCGGTGCTCGCAGGCTCGGCA |
| 41A | ATCTATGTGGCTGGACATTCGGTTTTTTTAGTCCATGG |
| 41B | GCACGGCGTACGGACTCACGGTGTATCTATGTGGCTGGACATTCGG |
| 41C | CCATGGACTTTTCCGAATGTCCAGCCACATAGATTGGTCGGTGCTCGCAGGCTCGGCA |
| 42A | AAATGCAATAGATATGGCTGGATTTTTGAAGCCA |
| 42B | GCACGGCGTACGGACTCACGGTGTAAATGCAATAGATATGGCTGGA |
| 42C | TGGCTTCTTTTCCAGCCATATCTATTGCATTTTGGTCGGTGCTCGCAGGCTCGGCA |
| 43A | TGCGAATTACGTGAACTAGAGCTTTATGTTTAGTC |
| 43B | GCACGGCGTACGGACTCACGGTGTTGCGAATTACGTGAACTAGAGC |
| 43C | GACTAAACATTTTTTTTTTGCTCTAGTTCACGTAATTCGCATGGTCGGTGCTCGCAGGCTCGGCA |
| 44A | CGCCTGTTCGTCGCGTGGCGCATTTTTTCTACAAC |
| 44B | GCACGGCGTACGGACTCACGGTGTCGCCTGTTCGTCGCGTGGCGCA |
| 44C | GTTGTAGATTTTTTTTGCGCCACGCGACGAACAGGCGTGGTCGGTGCTCGCAGGCTCGGCA |
| 45A | TGTCTCGGAGGATTTTGTACCATTTTTCTAAGCTAGG |
| 45B | GCACGGCGTACGGACTCACGGTGTTGTCTCGGAGGATTTTGTACCA |
| 45C | CCTAGCTTAGTGGTACAAAATCCTCCGAGACATGGTCGGTGCTCGCAGGCTCGGCA |
| 46A | CAAGGAACTCCGTATGTTCCAGTTCGGGTCCGGCA |
| 46B | GCACGGCGTACGGACTCACGGTGTCAAGGAACTCCGTATGTTCCAG |
| 46C | TGCCGGACCCGCTGGAACATACGGAGTTCCTTGTGGTCGGTGCTCGCAGGCTCGGCA |
| 47A | TCCTCGCTACATTGTTATGATTTTAACAGTACCTAG |
| 47B | GCACGGCGTACGGACTCACGGTGTTCCTCGCTACATTGTTATGATT |
| 47C | CTAGGTACTGTTTTTTTTTTAATCATAACAATGTAGCGAGGATGGTCGGTGCTCGCAGGCTCGGCA |
| 48A | GCTAGGGAGGATCGAAGATTGCTTATCATCC |
| 48B | GCACGGCGTACGGACTCACGGTGTGCTAGGGAGGATCGAAGATTGC |
| 48C | GGATGATTGCAATCTTCGATCCTCCCTAGCTGGTCGGTGCTCGCAGGCTCGGCA |
| 49A | GTCACGTCATCATTCGAATCGGTTTCATAG |
| 49B | GCACGGCGTACGGACTCACGGTGTGTCACGTCATCATTCGAATCGG |
| 49C | CTATGTTTCCGATTCGAATGATGACGTGACTGGTCGGTGCTCGCAGGCTCGGCA |
| 50A | TCCTCGACTCAACCCCTACGCTTTACGTCGGA |
| 50B | GCACGGCGTACGGACTCACGGTGTTCCTCGACTCAACCCCTACGCT |
| 50C | TCCGACGTATAGCGTAGGGGTTGAGTCGAGGATGGTCGGTGCTCGCAGGCTCGGCA |
| 51A | GCTCTCATATCCCCCGCAGATATTTTTTTGAACAATGCTC |
| 51B | GCACGGCGTACGGACTCACGGTGTGCTCTCATATCCCCCGCAGATA |
| 51C | GAGCATTGTTCTTTATCTGCGGGGGATATGAGAGCTGGTCGGTGCTCGCAGGCTCGGCA |
| 52A | CGCGCGCGTAATTAAGCGTTTATTACTCAAAC |
| 52B | GCACGGCGTACGGACTCACGGTGTCGCGCGCGTAATTAAGCGTTTA |
| 52C | GTTTGAGTTTTTTTTAAACGCTTAATTACGCGCGCGTGGTCGGTGCTCGCAGGCTCGGCA |
| 53A | AGTGGTTTCGGAGGGTTCACTGTTTTCCACCTC |
| 53B | GCACGGCGTACGGACTCACGGTGTAGTGGTTTCGGAGGGTTCACTG |
| 53C | GAGGTGGTTTTCAGTGAACCCTCCGAAACCACTTGGTCGGTGCTCGCAGGCTCGGCA |
| 54A | CACACACACCTAATAATCCCTATTTTTGAGGCCGCTCACTT |
| 54B | GCACGGCGTACGGACTCACGGTGTCACACACACCTAATAATCCCTA |
| 54C | AAGTGAGCGGCCTCTTTTTTTTTTTAGGGATTATTAGGTGTGTGTGTGGTCGGTGCTCGCAGGCTCGGCA |
| 55A | GGGCGATCCAACGACGTGACACTTTTTTTTATCGGGGCGG |
| 55B | GCACGGCGTACGGACTCACGGTGTGGGCGATCCAACGACGTGACAC |
| 55C | CCGCCCCGATATTTTTGTGTCACGTCGTTGGATCGCCCTGGTCGGTGCTCGCAGGCTCGGCA |
| 56A | CGATTATCGCAAAAAACCTGGTTTTTTTTCAGCTCA |
| 56B | GCACGGCGTACGGACTCACGGTGTCGATTATCGCAAAAAACCTGGT |
| 56C | TGAGCTGATTACCAGGTTTTTTGCGATAATCGTGGTCGGTGCTCGCAGGCTCGGCA |
| 57A | TCCAGTTTCTTCACCTGATCGATTTTTTTTTAGTTTGCTCTTGCA |
| 57B | GCACGGCGTACGGACTCACGGTGTTCCAGTTTCTTCACCTGATCGA |
| 57C | TGCAAGAGCAAACTTTTTTTCGATCAGGTGAAGAAACTGGATGGTCGGTGCTCGCAGGCTCGGCA |
| 58A | TTCAGGCGCTCAATGTAGGATATTTAGCAGGATAT |
| 58B | GCACGGCGTACGGACTCACGGTGTTTCAGGCGCTCAATGTAGGATA |
| 58C | ATATCCTGCTTTTTTTTTTTATCCTACATTGAGCGCCTGAATGGTCGGTGCTCGCAGGCTCGGCA |
| 59A | CTCTACGTCATTGGTTACATCATTTTTTTGATCGAGGATA |
| 59B | GCACGGCGTACGGACTCACGGTGTCTCTACGTCATTGGTTACATCA |
| 59C | TATCCTCGATCTTGATGTAACCAATGACGTAGAGTGGTCGGTGCTCGCAGGCTCGGCA |
| 60A | AAGCACGAAATACAGTTGTAATTTTTTTTTTCGCCATCCCA |
| 60B | GCACGGCGTACGGACTCACGGTGTAAGCACGAAATACAGTTGTAAT |
| 60C | TGGGATGGCGTTTTTTTTATTACAACTGTATTTCGTGCTTTGGTCGGTGCTCGCAGGCTCGGCA |
| 61A | CCGATAACGTCCCGTTCACCGTCGAAAGTCGAG |
| 61B | GCACGGCGTACGGACTCACGGTGTCCGATAACGTCCCGTTCACCGT |
| 61C | CTCGACTTTCGTTTTTTTTTTACGGTGAACGGGACGTTATCGGTGGTCGGTGCTCGCAGGCTCGGCA |
| 62A | CAGGAACAACAAGAATTGCCTTTTTTTTTTAGTCGCCTCTTGG |
| 62B | GCACGGCGTACGGACTCACGGTGTCAGGAACAACAAGAATTGCCTT |
| 62C | CCAAGAGGCGACTTTTTTTTTTTAAGGCAATTCTTGTTGTTCCTGTGGTCGGTGCTCGCAGGCTCGGCA |
| 63A | GTCGACAGAGACACACGGGATGTTTATCCGAAAAA |
| 63B | GCACGGCGTACGGACTCACGGTGTGTCGACAGAGACACACGGGATG |
| 63C | TTTTTCGGATTTTTTTCATCCCGTGTGTCTCTGTCGACTGGTCGGTGCTCGCAGGCTCGGCA |
| 64A | CGTCAATCGAATACTCCCTTGGTCCTTCTTGGTCCGA |
| 64B | GCACGGCGTACGGACTCACGGTGTCGTCAATCGAATACTCCCTTGG |
| 64C | TCGGACCAAGAAGGTTTTCCAAGGGAGTATTCGATTGACGTGGTCGGTGCTCGCAGGCTCGGCA |
| 65A | CCGTCCACGCCGCAACACCGGTTTTTAGCGA |
| 65B | GCACGGCGTACGGACTCACGGTGTCCGTCCACGCCGCAACACCGGT |
| 65C | TCGCTATACCGGTGTTGCGGCGTGGACGGTGGTCGGTGCTCGCAGGCTCGGCA |
| 66A | TAGGGTACTACTATTCAAAAATTTTTTTTTAGCTGACGCGGGC |
| 66B | GCACGGCGTACGGACTCACGGTGTTAGGGTACTACTATTCAAAAAT |
| 66C | GCCCGCGTCAGCTTTTTTATTTTTGAATAGTAGTACCCTATGGTCGGTGCTCGCAGGCTCGGCA |
| 67A | CCGTCATTACTCAAGGTACGGTTAAAC |
| 67B | GCACGGCGTACGGACTCACGGTGTCCGTCATTACTCAAGGTACGGT |
| 67C | GTTTTTACCGTACCTTGAGTAATGACGGTGGTCGGTGCTCGCAGGCTCGGCA |
| 68A | TCGATTATAGGCACCGAGCTCCTTTACGCTCGCCCT |
| 68B | GCACGGCGTACGGACTCACGGTGTTCGATTATAGGCACCGAGCTCC |
| 68C | AGGGCGAGCGTATTTTTTGGAGCTCGGTGCCTATAATCGATGGTCGGTGCTCGCAGGCTCGGCA |
| 69A | CACGAGGCGATCCTAAGACGCCCACCGTGACACGT |
| 69B | GCACGGCGTACGGACTCACGGTGTCACGAGGCGATCCTAAGACGCC |
| 69C | ACGTGTCACGGTGTTTTGGCGTCTTAGGATCGCCTCGTGTGGTCGGTGCTCGCAGGCTCGGCA |
| 70A | TGGCAATGACTCCAACCTGACATTTTTCACCTCT |
| 70B | GCACGGCGTACGGACTCACGGTGTTGGCAATGACTCCAACCTGACA |
| 70C | AGAGGTGAATGTCAGGTTGGAGTCATTGCCATGGTCGGTGCTCGCAGGCTCGGCA |
| 71A | TAACACATCTTTTGGGGCCAAATTTTTTTTTTTTAGTACGTG |
| 71B | GCACGGCGTACGGACTCACGGTGTTAACACATCTTTTGGGGCCAAA |
| 71C | CACGTACTAAATTTTTTTTTGGCCCCAAAAGATGTGTTATGGTCGGTGCTCGCAGGCTCGGCA |
| 72A | GTGGTCCCGTCCTGTCTAGACTTTTTCCTGCTTGTTGGAT |
| 72B | GCACGGCGTACGGACTCACGGTGTGTGGTCCCGTCCTGTCTAGACT |
| 72C | ATCCAACAAGCAGGTTTTAGTCTAGACAGGACGGGACCACTGGTCGGTGCTCGCAGGCTCGGCA |
| 73A | GTAGATATCCTTGTTGCCAGCCTTTTTTTTCTTGGTCCTTAGGT |
| 73B | GCACGGCGTACGGACTCACGGTGTGTAGATATCCTTGTTGCCAGCC |
| 73C | ACCTAAGGACCAAGTTTTTTTTTTGGCTGGCAACAAGGATATCTACTGGTCGGTGCTCGCAGGCTCGGCA |
| 74A | GCCCAACTCCTACGGCTCGCACTTTTTTTTTACACCCACTACG |
| 74B | GCACGGCGTACGGACTCACGGTGTGCCCAACTCCTACGGCTCGCAC |
| 74C | CGTAGTGGGTGTTTTTTTGTGCGAGCCGTAGGAGTTGGGCTGGTCGGTGCTCGCAGGCTCGGCA |
| 75A | CACGTCATATCCTTTATCCACCTTTTTTTTTAATGCACTTTCATC |
| 75B | GCACGGCGTACGGACTCACGGTGTCACGTCATATCCTTTATCCACC |
| 75C | GATGAAAGTGCATTGGTGGATAAAGGATATGACGTGTGGTCGGTGCTCGCAGGCTCGGCA |
| 76A | CCTGCGCAGCCACGACAGCATCTTTTTTTTTTGCTTTGTGGCCGC |
| 76B | GCACGGCGTACGGACTCACGGTGTCCTGCGCAGCCACGACAGCATC |
| 76C | GCGGCCACAAAGCTTTGATGCTGTCGTGGCTGCGCAGGTGGTCGGTGCTCGCAGGCTCGGCA |
| 77A | CTACTCGACTATCTGATAAACCTTAGACTTGG |
| 77B | GCACGGCGTACGGACTCACGGTGTCTACTCGACTATCTGATAAACC |
| 77C | CCAAGTCTGGTTTATCAGATAGTCGAGTAGTGGTCGGTGCTCGCAGGCTCGGCA |
| 78A | GTGACACGCGTCCTACATACGACCTT |
| 78B | GCACGGCGTACGGACTCACGGTGTGTGACACGCGTCCTACATACGA |
| 78C | AAGGTTTTCGTATGTAGGACGCGTGTCACTGGTCGGTGCTCGCAGGCTCGGCA |
| 79A | CTGCACACCCAGCATACTTGCGTTTTTTACGGAGCGCCTACG |
| 79B | GCACGGCGTACGGACTCACGGTGTCTGCACACCCAGCATACTTGCG |
| 79C | CGTAGGCGCTCCGTTTTTTTTTCGCAAGTATGCTGGGTGTGCAGTGGTCGGTGCTCGCAGGCTCGGCA |
| 80A | GCTCAGTATACGTACGTTTAGGTTTTTATAGA |
| 80B | GCACGGCGTACGGACTCACGGTGTGCTCAGTATACGTACGTTTAGG |
| 80C | TCTATATTTTCCTAAACGTACGTATACTGAGCTGGTCGGTGCTCGCAGGCTCGGCA |
| 81A | AGGTTAACTGCACCCAACATTCTTGTATGTCTAAT |
| 81B | GCACGGCGTACGGACTCACGGTGTAGGTTAACTGCACCCAACATTC |
| 81C | ATTAGACATACATTGAATGTTGGGTGCAGTTAACCTTGGTCGGTGCTCGCAGGCTCGGCA |
| 82A | ATCCCAGTCCGGACGACCGCCTTTTTGATCGCCCTGGCG |
| 82B | GCACGGCGTACGGACTCACGGTGTATCCCAGTCCGGACGACCGCCT |
| 82C | CGCCAGGGCGATCTTTTTTTTTAGGCGGTCGTCCGGACTGGGATTGGTCGGTGCTCGCAGGCTCGGCA |
| 83A | TAAAGCATTTAACGCCGTCCCTTTTAGAGG |
| 83B | GCACGGCGTACGGACTCACGGTGTTAAAGCATTTAACGCCGTCCCT |
| 83C | CCTCTAGGGACGGCGTTAAATGCTTTATGGTCGGTGCTCGCAGGCTCGGCA |
| 84A | CAATGGTGACTCATATATGATGTTTTTTTTGTTA |
| 84B | GCACGGCGTACGGACTCACGGTGTCAATGGTGACTCATATATGATG |
| 84C | TAACAAATTTTTCATCATATATGAGTCACCATTGTGGTCGGTGCTCGCAGGCTCGGCA |
| 85A | ATTGACCTGCCATGCCGAGAACTTCTCGGGTTGTAATC |
| 85B | GCACGGCGTACGGACTCACGGTGTATTGACCTGCCATGCCGAGAAC |
| 85C | GATTACAACCCGAGTTTTGTTCTCGGCATGGCAGGTCAATTGGTCGGTGCTCGCAGGCTCGGCA |
| 86A | ATCCATGAGAATCGGGAATTCCTTTTGCTCGAATA |
| 86B | GCACGGCGTACGGACTCACGGTGTATCCATGAGAATCGGGAATTCC |
| 86C | TATTCGAGCTTTTTTTTGGAATTCCCGATTCTCATGGATTGGTCGGTGCTCGCAGGCTCGGCA |
| 87A | GGTGGGTGTCTGGATGGGAGACTTTTTTTTATGTGGGCCC |
| 87B | GCACGGCGTACGGACTCACGGTGTGGTGGGTGTCTGGATGGGAGAC |
| 87C | GGGCCCACATTTTTTTGTCTCCCATCCAGACACCCACCTGGTCGGTGCTCGCAGGCTCGGCA |
| 88A | TTCGATGGGCAAAGGACACGAATTATTACGTGTC |
| 88B | GCACGGCGTACGGACTCACGGTGTTTCGATGGGCAAAGGACACGAA |
| 88C | GACACGTAATTTTTCGTGTCCTTTGCCCATCGAATGGTCGGTGCTCGCAGGCTCGGCA |
| 89A | ATTAGGCTCAATTAGTCCGGAATTTTTTTTTAAGGGTCTCAGGTT |
| 89B | GCACGGCGTACGGACTCACGGTGTATTAGGCTCAATTAGTCCGGAA |
| 89C | AACCTGAGACCCTTTTTCCGGACTAATTGAGCCTAATTGGTCGGTGCTCGCAGGCTCGGCA |
| 90A | GATCCAAATACCTACCATACAATACAATCAG |
| 90B | GCACGGCGTACGGACTCACGGTGTGATCCAAATACCTACCATACAA |
| 90C | CTGATTGTATTGTATGGTAGGTATTTGGATCTGGTCGGTGCTCGCAGGCTCGGCA |
| 91A | TCAGCTCTCGGATGTCGAACATTTTTTTCAGCGGGT |
| 91B | GCACGGCGTACGGACTCACGGTGTTCAGCTCTCGGATGTCGAACAT |
| 91C | ACCCGCTGTTTTTTTATGTTCGACATCCGAGAGCTGATGGTCGGTGCTCGCAGGCTCGGCA |
| 92A | CCACATCTCCGCTTGATGGAACTTTTTTTTTTGCGCGTAAAC |
| 92B | GCACGGCGTACGGACTCACGGTGTCCACATCTCCGCTTGATGGAAC |
| 92C | GTTTACGCGCATTTTTTTTTTGTTCCATCAAGCGGAGATGTGGTGGTCGGTGCTCGCAGGCTCGGCA |
| 93A | CGCAGCCGGACAAAGGATCGCTTTTTTTTTCTCATTGAG |
| 93B | GCACGGCGTACGGACTCACGGTGTCGCAGCCGGACAAAGGATCGCT |
| 93C | CTCAATGAGTTTTTTAGCGATCCTTTGTCCGGCTGCGTGGTCGGTGCTCGCAGGCTCGGCA |
| 94A | TGCATATTGCGTCAACTTGGATTTTTTTTACTAAGGTAA |
| 94B | GCACGGCGTACGGACTCACGGTGTTGCATATTGCGTCAACTTGGAT |
| 94C | TTACCTTAGTTTTTATCCAAGTTGACGCAATATGCATGGTCGGTGCTCGCAGGCTCGGCA |
| 95A | TCCTGTTTAGTTCTGGCAACCCTTTTATCGTGGCCG |
| 95B | GCACGGCGTACGGACTCACGGTGTTCCTGTTTAGTTCTGGCAACCC |
| 95C | CGGCCACGATGGGTTGCCAGAACTAAACAGGATGGTCGGTGCTCGCAGGCTCGGCA |
| 96A | TACTTTTACGCTGAATACCGATGGCTCTG |
| 96B | GCACGGCGTACGGACTCACGGTGTTACTTTTACGCTGAATACCGAT |
| 96C | CAGAGCCTATCGGTATTCAGCGTAAAAGTATGGTCGGTGCTCGCAGGCTCGGCA |
| 97A | AGTGTGAATAGATCACCAAGAGCTCAAGATATTT |
| 97B | GCACGGCGTACGGACTCACGGTGTAGTGTGAATAGATCACCAAGAG |
| 97C | AAATATCTTGAGTTTTTTTTCTCTTGGTGATCTATTCACACTTGGTCGGTGCTCGCAGGCTCGGCA |
| 98A | GCTATAAAGCCTTAGGAAACGCTAACCACG |
| 98B | GCACGGCGTACGGACTCACGGTGTGCTATAAAGCCTTAGGAAACGC |
| 98C | CGTGGTTTTTTTGCGTTTCCTAAGGCTTTATAGCTGGTCGGTGCTCGCAGGCTCGGCA |
| 99A | TTCATGACACATGAACCGATTCTTTTTTTTTTCTGGCCGGTCAGC |
| 99B | GCACGGCGTACGGACTCACGGTGTTTCATGACACATGAACCGATTC |
| 99C | GCTGACCGGCCAGTTTGAATCGGTTCATGTGTCATGAATGGTCGGTGCTCGCAGGCTCGGCA |
| 100A | ACCTGTCATACACCGATAACGAAGGCAGGATG |
| 100B | GCACGGCGTACGGACTCACGGTGTACCTGTCATACACCGATAACGA |
| 100C | CATCCTGCCTTCGTTATCGGTGTATGACAGGTTGGTCGGTGCTCGCAGGCTCGGCA |
| 101A | TAAGAGCGCAATAAGGCAGTTTTTTTTTTTTTGCACAAATTTACAA |
| 101B | GCACGGCGTACGGACTCACGGTGTTAAGAGCGCAATAAGGCAGTTT |
| 101C | TTGTAAATTTGTGCTTTTTTTTAAACTGCCTTATTGCGCTCTTATGGTCGGTGCTCGCAGGCTCGGCA |
| 102A | AATTTTGTATTCTCTTCAAAGGTTTTTTTTGAGGGAGAAGAC |
| 102B | GCACGGCGTACGGACTCACGGTGTAATTTTGTATTCTCTTCAAAGG |
| 102C | GTCTTCTCCCTCTTTTTTTTTTCCTTTGAAGAGAATACAAAATTTGGTCGGTGCTCGCAGGCTCGGCA |
| 103A | AGTCACGACGTCATTTCTTATATTTTTATTAGA |
| 103B | GCACGGCGTACGGACTCACGGTGTAGTCACGACGTCATTTCTTATA |
| 103C | TCTAATATATAAGAAATGACGTCGTGACTTGGTCGGTGCTCGCAGGCTCGGCA |
| 104A | GAGAAAGGCGTGTTGGTTCATCCTCCGCCA |
| 104B | GCACGGCGTACGGACTCACGGTGTGAGAAAGGCGTGTTGGTTCATC |
| 104C | TGGCGGAGTTGATGAACCAACACGCCTTTCTCTGGTCGGTGCTCGCAGGCTCGGCA |
| 105A | GATGCTCCGGTCGTAGTACACACGCCTGCTAGCCAA |
| 105B | GCACGGCGTACGGACTCACGGTGTGATGCTCCGGTCGTAGTACACA |
| 105C | TTGGCTAGCAGGCGTTTTTGTGTACTACGACCGGAGCATCTGGTCGGTGCTCGCAGGCTCGGCA |
| 106A | AGGCATCGGCCGTAACTTAACCTTTTTTTTTTAAACTCGGCT |
| 106B | GCACGGCGTACGGACTCACGGTGTAGGCATCGGCCGTAACTTAACC |
| 106C | AGCCGAGTTTATTTTTTTGGTTAAGTTACGGCCGATGCCTTGGTCGGTGCTCGCAGGCTCGGCA |
| 107A | GGCTGATATTCCGCGGCACGGATTTTGCCGCGGAGCC |
| 107B | GCACGGCGTACGGACTCACGGTGTGGCTGATATTCCGCGGCACGGA |
| 107C | GGCTCCGCGGCTTTTTTTTTCCGTGCCGCGGAATATCAGCCTGGTCGGTGCTCGCAGGCTCGGCA |
| 108A | CAATGATTCACTTTTTGGGACATCCAATAAT |
| 108B | GCACGGCGTACGGACTCACGGTGTCAATGATTCACTTTTTGGGACA |
| 108C | ATTATTGGATTTTTTTGTCCCAAAAAGTGAATCATTGTGGTCGGTGCTCGCAGGCTCGGCA |
| 109A | ATTCCGGGACGCCGCCGCTACTGTTTTAGAA |
| 109B | GCACGGCGTACGGACTCACGGTGTATTCCGGGACGCCGCCGCTACT |
| 109C | TTCTAAAACTTTTAGTAGCGGCGGCGTCCCGGAATTGGTCGGTGCTCGCAGGCTCGGCA |
| 110A | CACATGACTCGTACCGGCGTTGCCCGACCC |
| 110B | GCACGGCGTACGGACTCACGGTGTCACATGACTCGTACCGGCGTTG |
| 110C | GGGTCGGGCAACGCCGGTACGAGTCATGTGTGGTCGGTGCTCGCAGGCTCGGCA |
| 111A | CAGGACCCCGGTAGCGTCACTATTGGGG |
| 111B | GCACGGCGTACGGACTCACGGTGTCAGGACCCCGGTAGCGTCACTA |
| 111C | CCCCTAGTGACGCTACCGGGGTCCTGTGGTCGGTGCTCGCAGGCTCGGCA |
| 112A | GGATCACTCTTTTTCTTCTCTATATTGGGGCGGA |
| 112B | GCACGGCGTACGGACTCACGGTGTGGATCACTCTTTTTCTTCTCTA |
| 112C | TCCGCCCCAATTTTTTTAGAGAAGAAAAAGAGTGATCCTGGTCGGTGCTCGCAGGCTCGGCA |
| 113A | ATCTCCTCTAACCATACGCCACTTTTTTTCTTACCTC |
| 113B | GCACGGCGTACGGACTCACGGTGTATCTCCTCTAACCATACGCCAC |
| 113C | GAGGTAAGATTTTTGTGGCGTATGGTTAGAGGAGATTGGTCGGTGCTCGCAGGCTCGGCA |
| 114A | TGGATGAAAACGGATGGTTGTTTTTACGTCCACT |
| 114B | GCACGGCGTACGGACTCACGGTGTTGGATGAAAACGGATGGTTGTT |
| 114C | AGTGGACGTTAACAACCATCCGTTTTCATCCATGGTCGGTGCTCGCAGGCTCGGCA |
| 115A | AGCAAAGCGAACCCGTAGTGGGTTTCGTT |
| 115B | GCACGGCGTACGGACTCACGGTGTAGCAAAGCGAACCCGTAGTGGG |
| 115C | AACGTCCCACTACGGGTTCGCTTTGCTTGGTCGGTGCTCGCAGGCTCGGCA |
| 116A | GAGGGCTCACGGGAGCACTCGGGTACC |
| 116B | GCACGGCGTACGGACTCACGGTGTGAGGGCTCACGGGAGCACTCGG |
| 116C | GGTACTCCGAGTGCTCCCGTGAGCCCTCTGGTCGGTGCTCGCAGGCTCGGCA |
| 117A | GCCGCTCGGCGGCGCCAGTCGTTTTTCTAACGGACTTGT |
| 117B | GCACGGCGTACGGACTCACGGTGTGCCGCTCGGCGGCGCCAGTCGT |
| 117C | ACAAGTCCGTTAGTTTTTTTTACGACTGGCGCCGCCGAGCGGCTGGTCGGTGCTCGCAGGCTCGGCA |
| 118A | TCAGTCAGGAGTGATTTCTACATCAGA |
| 118B | GCACGGCGTACGGACTCACGGTGTTCAGTCAGGAGTGATTTCTACA |
| 118C | TCTGTGTAGAAATCACTCCTGACTGATGGTCGGTGCTCGCAGGCTCGGCA |
| 119A | GCCGGCCACGGGCTAATGGGGTTGTCAGGCGAATA |
| 119B | GCACGGCGTACGGACTCACGGTGTGCCGGCCACGGGCTAATGGGGT |
| 119C | TATTCGCCTGACATACCCCATTAGCCCGTGGCCGGCTGGTCGGTGCTCGCAGGCTCGGCA |
| 120A | GGTCCAAGCTACCTGGCTGTACTCCATATAGGAGGG |
| 120B | GCACGGCGTACGGACTCACGGTGTGGTCCAAGCTACCTGGCTGTAC |
| 120C | CCCTCCTATATGGAGTACAGCCAGGTAGCTTGGACCTGGTCGGTGCTCGCAGGCTCGGCA |
| 121A | GTCTTGAAGCCCTAAATTGCATTTTTTTTTCAGTTAGCTTGT |
| 121B | GCACGGCGTACGGACTCACGGTGTGTCTTGAAGCCCTAAATTGCAT |
| 121C | ACAAGCTAACTGTTATGCAATTTAGGGCTTCAAGACTGGTCGGTGCTCGCAGGCTCGGCA |
| 122A | GCGCTTAACGCACCTGCATTCCCCCAGCGCAG |
| 122B | GCACGGCGTACGGACTCACGGTGTGCGCTTAACGCACCTGCATTCC |
| 122C | CTGCGCTGGGGGAATGCAGGTGCGTTAAGCGCTGGTCGGTGCTCGCAGGCTCGGCA |
| 123A | CCCTCATTCCCCAACCGCTCATTTTTCCAAACACAGGCC |
| 123B | GCACGGCGTACGGACTCACGGTGTCCCTCATTCCCCAACCGCTCAT |
| 123C | GGCCTGTGTTTGGTTTATGAGCGGTTGGGGAATGAGGGTGGTCGGTGCTCGCAGGCTCGGCA |
| 124A | CTATAGAAAATTGACGTCTTATTTTTTTTTTACTTTTTAAC |
| 124B | GCACGGCGTACGGACTCACGGTGTCTATAGAAAATTGACGTCTTAT |
| 124C | GTTAAAAAGTTTTTTTTTTATAAGACGTCAATTTTCTATAGTGGTCGGTGCTCGCAGGCTCGGCA |
| 125A | CCGACGCACGCTTTCGGCCGACTTACTGAGAGCCGAT |
| 125B | GCACGGCGTACGGACTCACGGTGTCCGACGCACGCTTTCGGCCGAC |
| 125C | ATCGGCTCTCAGTTTTTTGTCGGCCGAAAGCGTGCGTCGGTGGTCGGTGCTCGCAGGCTCGGCA |
| 126A | GCCATGGACGATATTCGTCCATTTTGGCCT |
| 126B | GCACGGCGTACGGACTCACGGTGTGCCATGGACGATATTCGTCCAT |
| 126C | AGGCCTATGGACGAATATCGTCCATGGCTGGTCGGTGCTCGCAGGCTCGGCA |
| 127A | GGGTGCGTCCTCGACCCGTTCGTACACTAGCGTTA |
| 127B | GCACGGCGTACGGACTCACGGTGTGGGTGCGTCCTCGACCCGTTCG |
| 127C | TAACGCTAGTGTTTTTTTTTTTCGAACGGGTCGAGGACGCACCCTGGTCGGTGCTCGCAGGCTCGGCA |
| 128A | GCACGTGCACATAGTCTGCCGACGCTGTTGGCGT |
| 128B | GCACGGCGTACGGACTCACGGTGTGCACGTGCACATAGTCTGCCGA |
| 128C | ACGCCAACAGCGTTTTTTTTTTTCGGCAGACTATGTGCACGTGCTGGTCGGTGCTCGCAGGCTCGGCA |
| 129A | AGCTCAGCACATCGACGTAAAGTTTTTTCCGGTTACTATGAG |
| 129B | GCACGGCGTACGGACTCACGGTGTAGCTCAGCACATCGACGTAAAG |
| 129C | CTCATAGTAACCGGTTTTTTTTTCTTTACGTCGATGTGCTGAGCTTGGTCGGTGCTCGCAGGCTCGGCA |
| 130A | CTCTGCGCGGTGCCAGAGGTTATTTTTTCGGGTACGCGTC |
| 130B | GCACGGCGTACGGACTCACGGTGTCTCTGCGCGGTGCCAGAGGTTA |
| 130C | GACGCGTACCCGATTTTTTTTTTTAACCTCTGGCACCGCGCAGAGTGGTCGGTGCTCGCAGGCTCGGCA |
| 131A | TGCTGACCAGTATGATCTGCGGTTTTTTTGACCAGTCT |
| 131B | GCACGGCGTACGGACTCACGGTGTTGCTGACCAGTATGATCTGCGG |
| 131C | AGACTGGTCTTTTCCGCAGATCATACTGGTCAGCATGGTCGGTGCTCGCAGGCTCGGCA |
| 132A | GATGATTCGATATAGTTTAACTGAAGTCCGTGTACT |
| 132B | GCACGGCGTACGGACTCACGGTGTGATGATTCGATATAGTTTAACT |
| 132C | AGTACACGGACTTCAGTTAAACTATATCGAATCATCTGGTCGGTGCTCGCAGGCTCGGCA |
| 133A | TGCATAAAGGGAAAGATTCGACATATGA |
| 133B | GCACGGCGTACGGACTCACGGTGTTGCATAAAGGGAAAGATTCGAC |
| 133C | TCATATTTTGTCGAATCTTTCCCTTTATGCATGGTCGGTGCTCGCAGGCTCGGCA |
| 134A | TTCGTCGTTATGAAGCTTCCTCTTTTTTTTCAACCTTGTCA |
| 134B | GCACGGCGTACGGACTCACGGTGTTTCGTCGTTATGAAGCTTCCTC |
| 134C | TGACAAGGTTGAAGAGGAAGCTTCATAACGACGAATGGTCGGTGCTCGCAGGCTCGGCA |
| 135A | CCGGCCATGGCTCGCTGCAAGGTTGCCGC |
| 135B | GCACGGCGTACGGACTCACGGTGTCCGGCCATGGCTCGCTGCAAGG |
| 135C | GCGGCTTTTCCTTGCAGCGAGCCATGGCCGGTGGTCGGTGCTCGCAGGCTCGGCA |
| 136A | ACTTGTATTTTATCCTCTCGCCCGAT |
| 136B | GCACGGCGTACGGACTCACGGTGTACTTGTATTTTATCCTCTCGCC |
| 136C | ATCGTTGGCGAGAGGATAAAATACAAGTTGGTCGGTGCTCGCAGGCTCGGCA |
| 137A | GCCTCTCCCTTAAACGTTATTGGTCTTCTTAA |
| 137B | GCACGGCGTACGGACTCACGGTGTGCCTCTCCCTTAAACGTTATTG |
| 137C | TTAAGAAGACCAATAACGTTTAAGGGAGAGGCTGGTCGGTGCTCGCAGGCTCGGCA |
| 138A | AGAGCTGATATATAAGCGCAACTTTTTTTTGGTATTTTT |
| 138B | GCACGGCGTACGGACTCACGGTGTAGAGCTGATATATAAGCGCAAC |
| 138C | AAAAATACCTTTTTTTTGTTGCGCTTATATATCAGCTCTTGGTCGGTGCTCGCAGGCTCGGCA |
| 139A | ACGGATATCAACTATAGTTAGCTTTTGATTTCTACTA |
| 139B | GCACGGCGTACGGACTCACGGTGTACGGATATCAACTATAGTTAGC |
| 139C | TAGTAGAAATCTTTTTTGCTAACTATAGTTGATATCCGTTGGTCGGTGCTCGCAGGCTCGGCA |
| 140A | GTCGGTCCTCTCCCTTGGGCGCTTTGGTACCACGA |
| 140B | GCACGGCGTACGGACTCACGGTGTGTCGGTCCTCTCCCTTGGGCGC |
| 140C | TCGTGGTACCTTTTTTTTGCGCCCAAGGGAGAGGACCGACTGGTCGGTGCTCGCAGGCTCGGCA |
| 141A | ATCGTCAGGGCCCTAACTTTCTTTGTACCT |
| 141B | GCACGGCGTACGGACTCACGGTGTATCGTCAGGGCCCTAACTTTCT |
| 141C | AGGTACAAAGAAAGTTAGGGCCCTGACGATTGGTCGGTGCTCGCAGGCTCGGCA |
| 142A | CGCAGAACCAACCTTAAACGATCAGCACCCGA |
| 142B | GCACGGCGTACGGACTCACGGTGTCGCAGAACCAACCTTAAACGAT |
| 142C | TCGGGTGCTGTTATCGTTTAAGGTTGGTTCTGCGTGGTCGGTGCTCGCAGGCTCGGCA |
| 143A | TAGTTTTTCCATTGAGTTTAATTTTGTAA |
| 143B | GCACGGCGTACGGACTCACGGTGTTAGTTTTTCCATTGAGTTTAAT |
| 143C | TTACAATTAAACTCAATGGAAAAACTATGGTCGGTGCTCGCAGGCTCGGCA |
| 144A | TACTTGTCGCAACCTGCGTACTTTTTTTTTTTATTTTAACGTA |
| 144B | GCACGGCGTACGGACTCACGGTGTTACTTGTCGCAACCTGCGTACT |
| 144C | TACGTTAAAATTTTTTTTTTAGTACGCAGGTTGCGACAAGTATGGTCGGTGCTCGCAGGCTCGGCA |
| 145A | TGCCGCGACACCCGCGTTTCCTTGCGCC |
| 145B | GCACGGCGTACGGACTCACGGTGTTGCCGCGACACCCGCGTTTCCT |
| 145C | GGCGCTTTTAGGAAACGCGGGTGTCGCGGCATGGTCGGTGCTCGCAGGCTCGGCA |
| 146A | GCGCCGTGGCCCGGCTTGGTGGTTTTTACCAGG |
| 146B | GCACGGCGTACGGACTCACGGTGTGCGCCGTGGCCCGGCTTGGTGG |
| 146C | CCTGGTTTTTTCCACCAAGCCGGGCCACGGCGCTGGTCGGTGCTCGCAGGCTCGGCA |
| 147A | TGTGGAATATTTCACATTGGGTTTAATGTTTT |
| 147B | GCACGGCGTACGGACTCACGGTGTTGTGGAATATTTCACATTGGGT |
| 147C | AAAACATTTTTTACCCAATGTGAAATATTCCACATGGTCGGTGCTCGCAGGCTCGGCA |
| 148A | ACGCAACCTGGCAGTCCTACAGCCTACCATCT |
| 148B | GCACGGCGTACGGACTCACGGTGTACGCAACCTGGCAGTCCTACAG |
| 148C | AGATGGTAGGCTGTAGGACTGCCAGGTTGCGTTGGTCGGTGCTCGCAGGCTCGGCA |
| 149A | CGCGTTAGCGCCGCCACGGGGCTTTTTGCTCCAT |
| 149B | GCACGGCGTACGGACTCACGGTGTCGCGTTAGCGCCGCCACGGGGC |
| 149C | ATGGAGCTTTTTTGCCCCGTGGCGGCGCTAACGCGTGGTCGGTGCTCGCAGGCTCGGCA |
| 150A | GAGCATCAGATACGCGGCATAAGACT |
| 150B | GCACGGCGTACGGACTCACGGTGTGAGCATCAGATACGCGGCATAA |
| 150C | AGTCTTATGCCGCGTATCTGATGCTCTGGTCGGTGCTCGCAGGCTCGGCA |
| 151A | CGAAATTTCAGGACCGTTCTGATTTCGCTAA |
| 151B | GCACGGCGTACGGACTCACGGTGTCGAAATTTCAGGACCGTTCTGA |
| 151C | TTAGCGTTCAGAACGGTCCTGAAATTTCGTGGTCGGTGCTCGCAGGCTCGGCA |
| 152A | AGCCTCCGTTTGGGGCGGCCCACCAACGTAA |
| 152B | GCACGGCGTACGGACTCACGGTGTAGCCTCCGTTTGGGGCGGCCCA |
| 152C | TTACGTTGGTTTTTGGGCCGCCCCAAACGGAGGCTTGGTCGGTGCTCGCAGGCTCGGCA |
| 153A | CCCACGTCGAAATAAGATGCACTGCTCTAT |
| 153B | GCACGGCGTACGGACTCACGGTGTCCCACGTCGAAATAAGATGCAC |
| 153C | ATAGAGCAGTGCATCTTATTTCGACGTGGGTGGTCGGTGCTCGCAGGCTCGGCA |
| 154A | TAATCCACGCAATCTTCAAACTTAACTCTG |
| 154B | GCACGGCGTACGGACTCACGGTGTTAATCCACGCAATCTTCAAACT |
| 154C | CAGAGTTAAGTTTGAAGATTGCGTGGATTATGGTCGGTGCTCGCAGGCTCGGCA |
| 155A | CTATGGAAAACGGAACAGGGGCAAAGCT |
| 155B | GCACGGCGTACGGACTCACGGTGTCTATGGAAAACGGAACAGGGGC |
| 155C | AGCTTTGCCCCTGTTCCGTTTTCCATAGTGGTCGGTGCTCGCAGGCTCGGCA |
| 156A | GAAGTCCGAGACCCTTTCGCGGCGTAAC |
| 156B | GCACGGCGTACGGACTCACGGTGTGAAGTCCGAGACCCTTTCGCGG |
| 156C | GTTACGTTCCGCGAAAGGGTCTCGGACTTCTGGTCGGTGCTCGCAGGCTCGGCA |
| 157A | CACAATCTCTGCCTTCGCATTATTTTGGCTAGGCT |
| 157B | GCACGGCGTACGGACTCACGGTGTCACAATCTCTGCCTTCGCATTA |
| 157C | AGCCTAGCCTTTTTTAATGCGAAGGCAGAGATTGTGTGGTCGGTGCTCGCAGGCTCGGCA |
| 158A | GGTCGAAGCGGGAAACCAGGTGTCACGCACG |
| 158B | GCACGGCGTACGGACTCACGGTGTGGTCGAAGCGGGAAACCAGGTG |
| 158C | CGTGCGTGTTTTCACCTGGTTTCCCGCTTCGACCTGGTCGGTGCTCGCAGGCTCGGCA |
| 159A | AGTAATACGCGGGCATGCCGACTTTTGTCAGCGTATGT |
| 159B | GCACGGCGTACGGACTCACGGTGTAGTAATACGCGGGCATGCCGAC |
| 159C | ACATACGCTGACTTGTCGGCATGCCCGCGTATTACTTGGTCGGTGCTCGCAGGCTCGGCA |
| 160A | CTATTAACTCTACCTCAGTCTCTTTTTTTTTGTCCGCGCTAATA |
| 160B | GCACGGCGTACGGACTCACGGTGTCTATTAACTCTACCTCAGTCTC |
| 160C | TATTAGCGCGGACTTGAGACTGAGGTAGAGTTAATAGTGGTCGGTGCTCGCAGGCTCGGCA |
| 161A | AATGTAATGCGAACTACCCGGGTTTTTTTCGCACTACA |
| 161B | GCACGGCGTACGGACTCACGGTGTAATGTAATGCGAACTACCCGGG |
| 161C | TGTAGTGCGTTTTTTCCCGGGTAGTTCGCATTACATTTGGTCGGTGCTCGCAGGCTCGGCA |
| 162A | TCTTGAATATTATGATGTAATATTTTCGGCT |
| 162B | GCACGGCGTACGGACTCACGGTGTTCTTGAATATTATGATGTAATA |
| 162C | AGCCGTATTACATCATAATATTCAAGATGGTCGGTGCTCGCAGGCTCGGCA |
| 163A | GCTCTGTGTACATTTTCCATCCTTTTTTCAGGTCGG |
| 163B | GCACGGCGTACGGACTCACGGTGTGCTCTGTGTACATTTTCCATCC |
| 163C | CCGACCTGTGGATGGAAAATGTACACAGAGCTGGTCGGTGCTCGCAGGCTCGGCA |
| 164A | TACCACCTTCGCCGTCGCTATATTATCTG |
| 164B | GCACGGCGTACGGACTCACGGTGTTACCACCTTCGCCGTCGCTATA |
| 164C | CAGATTTTATAGCGACGGCGAAGGTGGTATGGTCGGTGCTCGCAGGCTCGGCA |
| 165A | CTGATCTAATAAAAATTGTCAATTTTTTTCAAGTCGTCTTG |
| 165B | GCACGGCGTACGGACTCACGGTGTCTGATCTAATAAAAATTGTCAA |
| 165C | CAAGACGACTTGTTTTTTTTTTTGACAATTTTTATTAGATCAGTGGTCGGTGCTCGCAGGCTCGGCA |
| 166A | CAAGTGTTTGACTAGTCCACTTTTAGTCAATAC |
| 166B | GCACGGCGTACGGACTCACGGTGTCAAGTGTTTGACTAGTCCACTT |
| 166C | GTATTGACTAATAAGTGGACTAGTCAAACACTTGTGGTCGGTGCTCGCAGGCTCGGCA |
| 167A | CCTGGCAGAGTATCGCCCGCCCTTACGATA |
| 167B | GCACGGCGTACGGACTCACGGTGTCCTGGCAGAGTATCGCCCGCCC |
| 167C | TATCGTTGGGCGGGCGATACTCTGCCAGGTGGTCGGTGCTCGCAGGCTCGGCA |
| 168A | ATCTTCGCCTAGCTCTTGGCGTTTTTTGTCGGT |
| 168B | GCACGGCGTACGGACTCACGGTGTATCTTCGCCTAGCTCTTGGCGT |
| 168C | ACCGACTTTTACGCCAAGAGCTAGGCGAAGATTGGTCGGTGCTCGCAGGCTCGGCA |
| 169A | TACACCCTTAGTACCCTATGGATTTTTTACTGTAACTA |
| 169B | GCACGGCGTACGGACTCACGGTGTTACACCCTTAGTACCCTATGGA |
| 169C | TAGTTACAGTTTTTTTTTTCCATAGGGTACTAAGGGTGTATGGTCGGTGCTCGCAGGCTCGGCA |
| 170A | ATTAGCAAGATATTTATTACGCTTTTAATAAGAAA |
| 170B | GCACGGCGTACGGACTCACGGTGTATTAGCAAGATATTTATTACGC |
| 170C | TTTCTTATTTTTTTTTGCGTAATAAATATCTTGCTAATTGGTCGGTGCTCGCAGGCTCGGCA |
| 171A | AGGGATCCTTTCTGTCCCCGTATTTTTTGTACTGAG |
| 171B | GCACGGCGTACGGACTCACGGTGTAGGGATCCTTTCTGTCCCCGTA |
| 171C | CTCAGTACTTTTTTTACGGGGACAGAAAGGATCCCTTGGTCGGTGCTCGCAGGCTCGGCA |
| 172A | AGCTTCTTTACCTACCCGGCGGTTTTTGGCCGCTGTCT |
| 172B | GCACGGCGTACGGACTCACGGTGTAGCTTCTTTACCTACCCGGCGG |
| 172C | AGACAGCGGCCCCGCCGGGTAGGTAAAGAAGCTTGGTCGGTGCTCGCAGGCTCGGCA |
| 173A | CACACTCCTCGATATTCTTCCCTTCCATCGGT |
| 173B | GCACGGCGTACGGACTCACGGTGTCACACTCCTCGATATTCTTCCC |
| 173C | ACCGATGGTTTGGGAAGAATATCGAGGAGTGTGTGGTCGGTGCTCGCAGGCTCGGCA |
| 174A | TCAGAATGATCAGAAGACCCTTCTATTAAAAT |
| 174B | GCACGGCGTACGGACTCACGGTGTTCAGAATGATCAGAAGACCCTT |
| 174C | ATTTTAATAGTTAAGGGTCTTCTGATCATTCTGATGGTCGGTGCTCGCAGGCTCGGCA |
| 175A | AATACGTGATATCTCATTATCACCGC |
| 175B | GCACGGCGTACGGACTCACGGTGTAATACGTGATATCTCATTATCA |
| 175C | GCGGTGATAATGAGATATCACGTATTTGGTCGGTGCTCGCAGGCTCGGCA |
| 176A | ACATGAGAAATTTTCCGTAAGCTTGCACC |
| 176B | GCACGGCGTACGGACTCACGGTGTACATGAGAAATTTTCCGTAAGC |
| 176C | GGTGCGCTTACGGAAAATTTCTCATGTTGGTCGGTGCTCGCAGGCTCGGCA |
| 177A | TGGATACCGACGGTCGAGCATTTTCTCTA |
| 177B | GCACGGCGTACGGACTCACGGTGTTGGATACCGACGGTCGAGCATT |
| 177C | TAGAGAATGCTCGACCGTCGGTATCCATGGTCGGTGCTCGCAGGCTCGGCA |
| 178A | ATAGCGGATTTTAGCCACGAAGTTTCTGGCAA |
| 178B | GCACGGCGTACGGACTCACGGTGTATAGCGGATTTTAGCCACGAAG |
| 178C | TTGCCAGTCTTCGTGGCTAAAATCCGCTATTGGTCGGTGCTCGCAGGCTCGGCA |
| 179A | AATCGCATGAGTGAGATCATTCTTTTTTTTACCACCAAG |
| 179B | GCACGGCGTACGGACTCACGGTGTAATCGCATGAGTGAGATCATTC |
| 179C | CTTGGTGGTTTTGAATGATCTCACTCATGCGATTTGGTCGGTGCTCGCAGGCTCGGCA |
| 180A | GGCTCTACAGACACGCGATAACAACAACTTC |
| 180B | GCACGGCGTACGGACTCACGGTGTGGCTCTACAGACACGCGATAAC |
| 180C | GAAGTTGTTGTTATCGCGTGTCTGTAGAGCCTGGTCGGTGCTCGCAGGCTCGGCA |
| 181A | CTCTCACTATGTAATACTATACTCGT |
| 181B | GCACGGCGTACGGACTCACGGTGTCTCTCACTATGTAATACTATAC |
| 181C | ACGAGTATAGTATTACATAGTGAGAGTGGTCGGTGCTCGCAGGCTCGGCA |
| 182A | GGCTTCACATGGGTCATAAGATTTTCCGGTCAACGGAAG |
| 182B | GCACGGCGTACGGACTCACGGTGTGGCTTCACATGGGTCATAAGAT |
| 182C | CTTCCGTTGACCGGTTTTATCTTATGACCCATGTGAAGCCTGGTCGGTGCTCGCAGGCTCGGCA |
| 183A | GCTGGTCGCGGCCCTCGGGACGTTGCTG |
| 183B | GCACGGCGTACGGACTCACGGTGTGCTGGTCGCGGCCCTCGGGACG |
| 183C | CAGCTTTCGTCCCGAGGGCCGCGACCAGCTGGTCGGTGCTCGCAGGCTCGGCA |
| 184A | CCTATCTGCAATAGCTTCTGGTTTTTCTCTCATTC |
| 184B | GCACGGCGTACGGACTCACGGTGTCCTATCTGCAATAGCTTCTGGT |
| 184C | GAATGAGAGATTTTTTTTTACCAGAAGCTATTGCAGATAGGTGGTCGGTGCTCGCAGGCTCGGCA |
| 185A | CACGCATTGTCACGTCTCACATTTTTTTTTTTTATAGGAGGCTTA |
| 185B | GCACGGCGTACGGACTCACGGTGTCACGCATTGTCACGTCTCACAT |
| 185C | TAAGCCTCCTATAATTTTTTTATGTGAGACGTGACAATGCGTGTGGTCGGTGCTCGCAGGCTCGGCA |
| 186A | AGGGAATAGTTCAGAACATTCGTTTTTTTTTCTATAGTTTAC |
| 186B | GCACGGCGTACGGACTCACGGTGTAGGGAATAGTTCAGAACATTCG |
| 186C | GTAAACTATAGTTCGAATGTTCTGAACTATTCCCTTGGTCGGTGCTCGCAGGCTCGGCA |
| 187A | TATACCACGTATTAGCCACCGTTTTTTTTTTCAGGAGATTA |
| 187B | GCACGGCGTACGGACTCACGGTGTTATACCACGTATTAGCCACCGT |
| 187C | TAATCTCCTGATTTTTTTTTTACGGTGGCTAATACGTGGTATATGGTCGGTGCTCGCAGGCTCGGCA |
| 188A | TTGACTGAAGTGTTACACTGTGTTTTTTACTGAGAGGC |
| 188B | GCACGGCGTACGGACTCACGGTGTTTGACTGAAGTGTTACACTGTG |
| 188C | GCCTCTCAGTATTTTTCACAGTGTAACACTTCAGTCAATGGTCGGTGCTCGCAGGCTCGGCA |
| 189A | GAGCCTCGGTAGCATTTTCTCCTACTTGAAT |
| 189B | GCACGGCGTACGGACTCACGGTGTGAGCCTCGGTAGCATTTTCTCC |
| 189C | ATTCAAGTATGGAGAAAATGCTACCGAGGCTCTGGTCGGTGCTCGCAGGCTCGGCA |
| 190A | GCTTATAGCCTACAGTTGGCTATTTTTTTTTTCGAAACG |
| 190B | GCACGGCGTACGGACTCACGGTGTGCTTATAGCCTACAGTTGGCTA |
| 190C | CGTTTCGAATTTTTAGCCAACTGTAGGCTATAAGCTGGTCGGTGCTCGCAGGCTCGGCA |
| 191A | GTGGGATAATCTCTCGGCCGGTTTTTTTTGTTATTAGTA |
| 191B | GCACGGCGTACGGACTCACGGTGTGTGGGATAATCTCTCGGCCGGT |
| 191C | TACTAATAACATTTTTTTTTACCGGCCGAGAGATTATCCCACTGGTCGGTGCTCGCAGGCTCGGCA |
| 192A | GAGAAGAACCCAATGAAGCGTATTTTTGAGCGCACTAC |
| 192B | GCACGGCGTACGGACTCACGGTGTGAGAAGAACCCAATGAAGCGTA |
| 192C | GTAGTGCGCTCATTTTTTACGCTTCATTGGGTTCTTCTCTGGTCGGTGCTCGCAGGCTCGGCA |
| 193A | TCCACGATAACAGGACAAGTTTTTTTTTTTGGCGCGTATT |
| 193B | GCACGGCGTACGGACTCACGGTGTTCCACGATAACAGGACAAGTTT |
| 193C | AATACGCGCCATTTTTTAAACTTGTCCTGTTATCGTGGATGGTCGGTGCTCGCAGGCTCGGCA |
| 194A | GCTTGCGCGACGCCGCGCCTTGTTGTAATG |
| 194B | GCACGGCGTACGGACTCACGGTGTGCTTGCGCGACGCCGCGCCTTG |
| 194C | CATTACATCAAGGCGCGGCGTCGCGCAAGCTGGTCGGTGCTCGCAGGCTCGGCA |
| 195A | CGGGCTGGTTCTTCCGCAGCCTTTTTTCTGCGAATACCAC |
| 195B | GCACGGCGTACGGACTCACGGTGTCGGGCTGGTTCTTCCGCAGCCT |
| 195C | GTGGTATTCGCAGTTAGGCTGCGGAAGAACCAGCCCGTGGTCGGTGCTCGCAGGCTCGGCA |
| 196A | CGGTGGGGCTTCATCAGATATTTTTTTCACTGACGAA |
| 196B | GCACGGCGTACGGACTCACGGTGTCGGTGGGGCTTCATCAGATATT |
| 196C | TTCGTCAGTGATTTTTTTTTAATATCTGATGAAGCCCCACCGTGGTCGGTGCTCGCAGGCTCGGCA |
| 197A | CGGCCCCTCTCGCACATGGGCAACAGACAAACATCG |
| 197B | GCACGGCGTACGGACTCACGGTGTCGGCCCCTCTCGCACATGGGCA |
| 197C | CGATGTTTGTCTGTTTTTTGCCCATGTGCGAGAGGGGCCGTGGTCGGTGCTCGCAGGCTCGGCA |
| 198A | ACGACGTACCATGGTCGTAGAATCCAACCCCCTTT |
| 198B | GCACGGCGTACGGACTCACGGTGTACGACGTACCATGGTCGTAGAA |
| 198C | AAAGGGGGTTGGATTCTACGACCATGGTACGTCGTTGGTCGGTGCTCGCAGGCTCGGCA |
| 199A | GCGAATATGATGAGTGAGCGCGTTTTTTATTCCGCTCCG |
| 199B | GCACGGCGTACGGACTCACGGTGTGCGAATATGATGAGTGAGCGCG |
| 199C | CGGAGCGGAATAACGCGCTCACTCATCATATTCGCTGGTCGGTGCTCGCAGGCTCGGCA |
| 200A | CGATAGATCTGCAGGTCCGGCGTTTTTTTTATCAG |
| 200B | GCACGGCGTACGGACTCACGGTGTCGATAGATCTGCAGGTCCGGCG |
| 200C | CTGATAAATTTTTCGCCGGACCTGCAGATCTATCGTGGTCGGTGCTCGCAGGCTCGGCA |
| 201A | TAGCCAGGAAGCCGGAGCGATGTTTACGGTGT |
| 201B | GCACGGCGTACGGACTCACGGTGTTAGCCAGGAAGCCGGAGCGATG |
| 201C | ACACCGTTTCATCGCTCCGGCTTCCTGGCTATGGTCGGTGCTCGCAGGCTCGGCA |
| 202A | GCATTGCTTATTAGCGTATCTTTTGATCGAACAGATTT |
| 202B | GCACGGCGTACGGACTCACGGTGTGCATTGCTTATTAGCGTATCTT |
| 202C | AAATCTGTTCGATCTTTTTAAGATACGCTAATAAGCAATGCTGGTCGGTGCTCGCAGGCTCGGCA |
| 203A | ATTGAAGAGCGGGGAACAGGTAGCGGATGT |
| 203B | GCACGGCGTACGGACTCACGGTGTATTGAAGAGCGGGGAACAGGTA |
| 203C | ACATCCGCTTTACCTGTTCCCCGCTCTTCAATTGGTCGGTGCTCGCAGGCTCGGCA |
| 204A | GGCGGCAAGGGACACAGTAGAATTTTGACGAC |
| 204B | GCACGGCGTACGGACTCACGGTGTGGCGGCAAGGGACACAGTAGAA |
| 204C | GTCGTCTTTCTACTGTGTCCCTTGCCGCCTGGTCGGTGCTCGCAGGCTCGGCA |
| 205A | ACGCATAATATACTCTAACCCGGCTAA |
| 205B | GCACGGCGTACGGACTCACGGTGTACGCATAATATACTCTAACCCG |
| 205C | TTAGCCGGGTTAGAGTATATTATGCGTTGGTCGGTGCTCGCAGGCTCGGCA |
| 206A | GGCGGCACTGCTCGTCGTATACCATTATGCTG |
| 206B | GCACGGCGTACGGACTCACGGTGTGGCGGCACTGCTCGTCGTATAC |
| 206C | CAGCATAATGTTTTGTATACGACGAGCAGTGCCGCCTGGTCGGTGCTCGCAGGCTCGGCA |
| 207A | GTTCAGCAGCGCGAACATCCTGTTATCCGTGCTGG |
| 207B | GCACGGCGTACGGACTCACGGTGTGTTCAGCAGCGCGAACATCCTG |
| 207C | CCAGCACGGATTTTTTTTTCAGGATGTTCGCGCTGCTGAACTGGTCGGTGCTCGCAGGCTCGGCA |
| 208A | CAGGTCAGACGCGCCGGGATAATTTTTTTCCCGGTGAC |
| 208B | GCACGGCGTACGGACTCACGGTGTCAGGTCAGACGCGCCGGGATAA |
| 208C | GTCACCGGGATTTTTATCCCGGCGCGTCTGACCTGTGGTCGGTGCTCGCAGGCTCGGCA |
| 209A | GCTACGCGACCTTTCTTCTCAATTTTTTTTTATTAGACTGCT |
| 209B | GCACGGCGTACGGACTCACGGTGTGCTACGCGACCTTTCTTCTCAA |
| 209C | AGCAGTCTAATTTTTTTTTTTGAGAAGAAAGGTCGCGTAGCTGGTCGGTGCTCGCAGGCTCGGCA |
| 210A | ATCCTCAAATGCGACTCAGTACTTTTAGCAT |
| 210B | GCACGGCGTACGGACTCACGGTGTATCCTCAAATGCGACTCAGTAC |
| 210C | ATGCTTTTGTACTGAGTCGCATTTGAGGATTGGTCGGTGCTCGCAGGCTCGGCA |
| 211A | TACGCACTTCTGGACAAAACTGTAGCACCC |
| 211B | GCACGGCGTACGGACTCACGGTGTTACGCACTTCTGGACAAAACTG |
| 211C | GGGTGCTTTTTCAGTTTTGTCCAGAAGTGCGTATGGTCGGTGCTCGCAGGCTCGGCA |
| 212A | CGGGTAACGCATAGCAGACAGGTATCTTG |
| 212B | GCACGGCGTACGGACTCACGGTGTCGGGTAACGCATAGCAGACAGG |
| 212C | CAAGATACCTGTCTGCTATGCGTTACCCGTGGTCGGTGCTCGCAGGCTCGGCA |
| 213A | AACCCCAAGGCTCGGACAGCTTTACTGGTTTTCTC |
| 213B | GCACGGCGTACGGACTCACGGTGTAACCCCAAGGCTCGGACAGCTT |
| 213C | GAGAAAACCAGTTTTTAAGCTGTCCGAGCCTTGGGGTTTGGTCGGTGCTCGCAGGCTCGGCA |
| 214A | CGGAGAGAATATAACAGGATCGTTTTTCTATGCA |
| 214B | GCACGGCGTACGGACTCACGGTGTCGGAGAGAATATAACAGGATCG |
| 214C | TGCATAGTTTCGATCCTGTTATATTCTCTCCGTGGTCGGTGCTCGCAGGCTCGGCA |
| 215A | CCGACATCACGGCCACAACCTATTTTGTGCTG |
| 215B | GCACGGCGTACGGACTCACGGTGTCCGACATCACGGCCACAACCTA |
| 215C | CAGCACAATTTTAGGTTGTGGCCGTGATGTCGGTGGTCGGTGCTCGCAGGCTCGGCA |
| 216A | GAAAGCTACACAAGCGATGACCTTTCGCGGCGCATTT |
| 216B | GCACGGCGTACGGACTCACGGTGTGAAAGCTACACAAGCGATGACC |
| 216C | AAATGCGCCGCGAATTTTTTTTGGTCATCGCTTGTGTAGCTTTCTGGTCGGTGCTCGCAGGCTCGGCA |
| 217A | GCTTAGAACTTACATGCTTAGGTTTCTCCGC |
| 217B | GCACGGCGTACGGACTCACGGTGTGCTTAGAACTTACATGCTTAGG |
| 217C | GCGGAGATTCCTAAGCATGTAAGTTCTAAGCTGGTCGGTGCTCGCAGGCTCGGCA |
| 218A | GACAAAACATTTTGCCTTAAGATTTTTGCCAGTGT |
| 218B | GCACGGCGTACGGACTCACGGTGTGACAAAACATTTTGCCTTAAGA |
| 218C | ACACTGGCTTTTTCTTAAGGCAAAATGTTTTGTCTGGTCGGTGCTCGCAGGCTCGGCA |
| 219A | AATGTGATACGCCGTGAGTCGGTTTTTTTCGGTTCAGG |
| 219B | GCACGGCGTACGGACTCACGGTGTAATGTGATACGCCGTGAGTCGG |
| 219C | CCTGAACCGTTTCCGACTCACGGCGTATCACATTTGGTCGGTGCTCGCAGGCTCGGCA |
| 220A | AGCTGGGAGCAGTCGGGTGAAATCATCGCG |
| 220B | GCACGGCGTACGGACTCACGGTGTAGCTGGGAGCAGTCGGGTGAAA |
| 220C | CGCGATGTTTCACCCGACTGCTCCCAGCTTGGTCGGTGCTCGCAGGCTCGGCA |
| 221A | TGCTACTATGGGTAGGGCCGCTTTCCCCTATGGAGTG |
| 221B | GCACGGCGTACGGACTCACGGTGTTGCTACTATGGGTAGGGCCGCT |
| 221C | CACTCCATAGGGGATTTAGCGGCCCTACCCATAGTAGCATGGTCGGTGCTCGCAGGCTCGGCA |
| 222A | CCCCTCGGCCTCCTAACGTTAGTTTTTTTTTTGGCTCCCTCATC |
| 222B | GCACGGCGTACGGACTCACGGTGTCCCCTCGGCCTCCTAACGTTAG |
| 222C | GATGAGGGAGCCATTCTAACGTTAGGAGGCCGAGGGGTGGTCGGTGCTCGCAGGCTCGGCA |
| 223A | GTGCCACGAACCCCAAGACCGCTTTTTTTTTTTTGGGGGAC |
| 223B | GCACGGCGTACGGACTCACGGTGTGTGCCACGAACCCCAAGACCGC |
| 223C | GTCCCCCAAAAGCGGTCTTGGGGTTCGTGGCACTGGTCGGTGCTCGCAGGCTCGGCA |
| 224A | TTAAGAAGCACCTCGGATGTGTTTTTTTTTTGGCTCGTGCTGAC |
| 224B | GCACGGCGTACGGACTCACGGTGTTTAAGAAGCACCTCGGATGTGT |
| 224C | GTCAGCACGAGCCAACACATCCGAGGTGCTTCTTAATGGTCGGTGCTCGCAGGCTCGGCA |
| 225A | TAGGGCTCGGATGCCAGCGACCTTTTTCGATCGCA |
| 225B | GCACGGCGTACGGACTCACGGTGTTAGGGCTCGGATGCCAGCGACC |
| 225C | TGCGATCGTGGTCGCTGGCATCCGAGCCCTATGGTCGGTGCTCGCAGGCTCGGCA |
| 226A | AGTTCGTCACACAGCGCTTTGTTTTCTAACTTGTTTC |
| 226B | GCACGGCGTACGGACTCACGGTGTAGTTCGTCACACAGCGCTTTGT |
| 226C | GAAACAAGTTAGACAAAGCGCTGTGTGACGAACTTGGTCGGTGCTCGCAGGCTCGGCA |
| 227A | ACGCGCCCCATCAACGCCATATTTTTCTCGCGA |
| 227B | GCACGGCGTACGGACTCACGGTGTACGCGCCCCATCAACGCCATAT |
| 227C | TCGCGAGATATATGGCGTTGATGGGGCGCGTTGGTCGGTGCTCGCAGGCTCGGCA |
| 228A | GTAGTTGGCACTCAGGTATACTTTTTTTTTTTAATTTACGGCAT |
| 228B | GCACGGCGTACGGACTCACGGTGTGTAGTTGGCACTCAGGTATACT |
| 228C | ATGCCGTAAATTTAGTATACCTGAGTGCCAACTACTGGTCGGTGCTCGCAGGCTCGGCA |
| 229A | AAATAGAGATTCTAAGGCTTTCTTTTATTGAAGA |
| 229B | GCACGGCGTACGGACTCACGGTGTAAATAGAGATTCTAAGGCTTTC |
| 229C | TCTTCAATTTTTTTTGAAAGCCTTAGAATCTCTATTTTGGTCGGTGCTCGCAGGCTCGGCA |
| F | TGCCGAGCCTGCGAGCACCGACCA/3`6-FAM/ |
| Q | /5`Dabcyl/ACACCGTGAGTCCGTACGCCGTGC |

**Table S7.** Kinetic parameters and experiment temperatures of 349 DNA strand displacement.

| ID | num | Temp | log10(k1) | log10(k2) | y1_k1 | y2_k1 | y3_k1 | y1_k2 | y2_k2 | y3_k2 | y_9000 |
| --- | --- | --- | --- | --- | --- | --- | --- | --- | --- | --- | --- |
| 1 | 1 | 25 | 4.786 | 3.435 | 0.391 | 0.495 | 0.596 | 0.404 | 0.404 | 0.404 | 0.211 |
| 2 | 2 | 25 | 5.466 | 4.060 | 0.405 | 0.418 | 0.434 | 0.415 | 0.415 | 0.415 | 0.392 |
| 3 | 3 | 25 | 5.854 | 4.450 | 0.700 | 0.712 | 0.725 | 0.724 | 0.724 | 0.725 | 0.712 |
| 4 | 4 | 25 | 5.322 | 3.777 | 0.044 | 0.055 | 0.072 | 0.060 | 0.060 | 0.068 | 0.050 |
| 5 | 5 | 25 | 5.349 | 3.810 | 0.159 | 0.220 | 0.267 | 0.217 | 0.239 | 0.254 | 0.191 |
| 6 | 6 | 25 | 6.209 | 4.802 | 0.654 | 0.680 | 0.694 | 0.680 | 0.680 | 0.694 | 0.676 |
| 7 | 7 | 25 | 7.557 | 6.215 | 0.719 | 0.729 | 0.744 | 0.719 | 0.729 | 0.744 | 0.731 |
| 8 | 8 | 25 | 5.962 | 4.537 | 0.561 | 0.591 | 0.613 | 0.589 | 0.591 | 0.613 | 0.588 |
| 9 | 9 | 25 | 5.226 | 3.670 | 0.064 | 0.096 | 0.124 | 0.094 | 0.107 | 0.118 | 0.078 |
| 10 | 10 | 25 | 6.276 | 4.879 | 0.686 | 0.701 | 0.714 | 0.697 | 0.701 | 0.714 | 0.700 |
| 11 | 11 | 25 | 5.273 | 3.753 | 0.032 | 0.042 | 0.050 | 0.044 | 0.045 | 0.046 | 0.034 |
| 12 | 12 | 25 | 5.911 | 4.510 | 0.627 | 0.642 | 0.656 | 0.642 | 0.642 | 0.656 | 0.642 |
| 13 | 13 | 25 | 5.230 | 3.746 | 0.393 | 0.502 | 0.572 | 0.522 | 0.522 | 0.522 | 0.387 |
| 14 | 14 | 25 | 5.292 | 3.706 | 0.155 | 0.162 | 0.167 | 0.193 | 0.193 | 0.193 | 0.159 |
| 15 | 15 | 25 | 5.165 | 3.682 | 0.304 | 0.387 | 0.456 | 0.402 | 0.402 | 0.402 | 0.283 |
| 16 | 16 | 25 | 4.915 | 3.739 | 0.315 | 0.333 | 0.374 | 0.218 | 0.218 | 0.218 | 0.181 |
| 17 | 17 | 25 | 5.951 | 4.540 | 0.534 | 0.548 | 0.562 | 0.548 | 0.548 | 0.562 | 0.548 |
| 18 | 18 | 25 | 5.244 | 3.701 | 0.201 | 0.264 | 0.333 | 0.294 | 0.294 | 0.315 | 0.216 |
| 19 | 19 | 25 | 6.891 | 5.519 | 0.742 | 0.752 | 0.757 | 0.742 | 0.752 | 0.757 | 0.750 |
| 20 | 20 | 25 | 6.141 | 4.738 | 0.665 | 0.687 | 0.704 | 0.683 | 0.687 | 0.703 | 0.685 |
| 21 | 21 | 25 | 5.283 | 3.735 | 0.061 | 0.098 | 0.121 | 0.087 | 0.108 | 0.114 | 0.080 |
| 22 | 22 | 25 | 7.726 | 6.485 | 0.897 | 0.916 | 0.921 | 0.897 | 0.916 | 0.921 | 0.911 |
| 23 | 23 | 25 | 5.463 | 3.962 | 0.402 | 0.500 | 0.589 | 0.526 | 0.529 | 0.556 | 0.466 |
| 24 | 24 | 25 | 6.845 | 5.438 | 0.735 | 0.772 | 0.786 | 0.735 | 0.772 | 0.786 | 0.764 |
| 25 | 25 | 25 | 5.151 | 3.695 | 0.189 | 0.232 | 0.274 | 0.232 | 0.232 | 0.232 | 0.171 |
| 26 | 26 | 25 | 5.359 | 3.852 | 0.331 | 0.418 | 0.477 | 0.445 | 0.445 | 0.445 | 0.361 |
| 27 | 27 | 25 | 6.345 | 4.945 | 0.659 | 0.677 | 0.691 | 0.661 | 0.677 | 0.691 | 0.676 |
| 28 | 28 | 25 | 6.221 | 4.823 | 0.660 | 0.672 | 0.698 | 0.675 | 0.675 | 0.698 | 0.677 |
| 29 | 29 | 25 | 5.431 | 3.932 | 0.345 | 0.417 | 0.499 | 0.442 | 0.442 | 0.469 | 0.389 |
| 30 | 30 | 25 | 6.783 | 5.394 | 0.768 | 0.794 | 0.823 | 0.768 | 0.794 | 0.823 | 0.795 |
| 31 | 31 | 25 | 5.265 | 3.927 | 0.165 | 0.178 | 0.187 | 0.154 | 0.154 | 0.154 | 0.148 |
| 32 | 32 | 25 | 5.731 | 4.306 | 0.568 | 0.608 | 0.673 | 0.608 | 0.609 | 0.647 | 0.603 |
| 33 | 33 | 25 | 7.250 | 5.878 | 0.772 | 0.783 | 0.789 | 0.772 | 0.783 | 0.789 | 0.781 |
| 34 | 34 | 25 | 4.678 | 3.065 | 0.105 | 0.168 | 0.219 | 0.200 | 0.222 | 0.225 | 0.059 |
| 35 | 35 | 25 | 5.282 | 3.930 | 0.505 | 0.557 | 0.596 | 0.504 | 0.504 | 0.504 | 0.457 |
| 36 | 36 | 25 | 6.088 | 4.688 | 0.685 | 0.706 | 0.722 | 0.705 | 0.706 | 0.722 | 0.704 |
| 37 | 37 | 25 | 5.613 | 4.165 | 0.643 | 0.685 | 0.722 | 0.714 | 0.715 | 0.719 | 0.676 |
| 38 | 38 | 25 | 4.938 | 3.524 | 0.340 | 0.440 | 0.514 | 0.403 | 0.403 | 0.403 | 0.235 |
| 39 | 39 | 25 | 6.256 | 4.862 | 0.633 | 0.645 | 0.665 | 0.645 | 0.645 | 0.665 | 0.648 |
| 40 | 40 | 25 | 5.905 | 4.484 | 0.612 | 0.646 | 0.657 | 0.646 | 0.646 | 0.657 | 0.638 |
| 41 | 41 | 25 | 5.427 | 3.916 | 0.406 | 0.503 | 0.619 | 0.543 | 0.543 | 0.586 | 0.471 |
| 42 | 42 | 25 | 5.231 | 3.690 | 0.052 | 0.068 | 0.084 | 0.074 | 0.074 | 0.078 | 0.055 |
| 43 | 43 | 25 | 5.476 | 4.008 | 0.353 | 0.427 | 0.506 | 0.431 | 0.436 | 0.466 | 0.400 |
| 44 | 44 | 25 | 5.066 | 3.557 | 0.166 | 0.214 | 0.265 | 0.231 | 0.231 | 0.235 | 0.141 |
| 45 | 45 | 25 | 6.543 | 5.154 | 0.683 | 0.704 | 0.714 | 0.689 | 0.704 | 0.714 | 0.700 |
| 46 | 46 | 25 | 6.026 | 4.622 | 0.709 | 0.731 | 0.758 | 0.732 | 0.732 | 0.758 | 0.732 |
| 47 | 47 | 25 | 6.269 | 4.877 | 0.705 | 0.727 | 0.743 | 0.710 | 0.727 | 0.743 | 0.725 |
| 48 | 48 | 25 | 5.378 | 3.887 | 0.300 | 0.359 | 0.413 | 0.379 | 0.379 | 0.380 | 0.319 |
| 49 | 49 | 25 | 5.346 | 3.786 | 0.062 | 0.081 | 0.116 | 0.087 | 0.090 | 0.111 | 0.077 |
| 50 | 50 | 25 | 6.073 | 4.665 | 0.667 | 0.687 | 0.697 | 0.684 | 0.687 | 0.695 | 0.683 |
| 51 | 51 | 25 | 6.425 | 5.027 | 0.687 | 0.701 | 0.741 | 0.700 | 0.701 | 0.741 | 0.710 |
| 52 | 52 | 25 | 5.091 | 3.545 | 0.202 | 0.304 | 0.415 | 0.312 | 0.346 | 0.390 | 0.209 |
| 53 | 53 | 25 | 5.413 | 3.882 | 0.109 | 0.140 | 0.185 | 0.146 | 0.147 | 0.176 | 0.136 |
| 54 | 54 | 25 | 5.726 | 4.277 | 0.609 | 0.657 | 0.678 | 0.669 | 0.669 | 0.678 | 0.643 |
| 55 | 55 | 25 | 5.999 | 4.586 | 0.706 | 0.741 | 0.770 | 0.744 | 0.744 | 0.770 | 0.739 |
| 56 | 56 | 25 | 5.294 | 3.781 | 0.237 | 0.296 | 0.370 | 0.320 | 0.320 | 0.341 | 0.252 |
| 57 | 57 | 25 | 5.961 | 4.552 | 0.647 | 0.671 | 0.686 | 0.671 | 0.671 | 0.686 | 0.668 |
| 58 | 58 | 25 | 5.445 | 3.993 | 0.451 | 0.521 | 0.578 | 0.534 | 0.534 | 0.534 | 0.478 |
| 59 | 59 | 25 | 6.083 | 4.685 | 0.744 | 0.764 | 0.800 | 0.762 | 0.764 | 0.800 | 0.769 |
| 60 | 60 | 25 | 5.690 | 4.245 | 0.584 | 0.616 | 0.638 | 0.636 | 0.636 | 0.637 | 0.607 |
| 61 | 61 | 25 | 5.405 | 4.017 | 0.560 | 0.586 | 0.637 | 0.580 | 0.580 | 0.580 | 0.538 |
| 62 | 62 | 25 | 5.654 | 4.228 | 0.507 | 0.522 | 0.531 | 0.531 | 0.531 | 0.531 | 0.519 |
| 63 | 63 | 25 | 5.343 | 3.852 | 0.323 | 0.390 | 0.456 | 0.415 | 0.415 | 0.417 | 0.343 |
| 64 | 64 | 25 | 6.313 | 4.920 | 0.722 | 0.745 | 0.765 | 0.733 | 0.745 | 0.765 | 0.744 |
| 65 | 65 | 25 | 5.468 | 3.884 | 0.041 | 0.055 | 0.065 | 0.057 | 0.061 | 0.065 | 0.052 |
| 66 | 66 | 25 | 5.940 | 4.532 | 0.648 | 0.670 | 0.687 | 0.670 | 0.670 | 0.687 | 0.668 |
| 67 | 67 | 25 | 5.079 | 3.517 | 0.083 | 0.132 | 0.181 | 0.130 | 0.152 | 0.172 | 0.089 |
| 68 | 68 | 25 | 5.909 | 4.476 | 0.532 | 0.568 | 0.586 | 0.551 | 0.568 | 0.586 | 0.558 |
| 69 | 69 | 25 | 5.326 | 3.979 | 0.424 | 0.459 | 0.481 | 0.414 | 0.414 | 0.414 | 0.396 |
| 70 | 70 | 25 | 6.343 | 4.935 | 0.638 | 0.689 | 0.711 | 0.638 | 0.689 | 0.711 | 0.679 |
| 71 | 71 | 25 | 6.062 | 4.645 | 0.567 | 0.598 | 0.637 | 0.585 | 0.598 | 0.637 | 0.601 |
| 72 | 72 | 25 | 6.284 | 4.892 | 0.711 | 0.721 | 0.746 | 0.721 | 0.721 | 0.746 | 0.726 |
| 73 | 73 | 25 | 5.922 | 4.509 | 0.556 | 0.574 | 0.587 | 0.574 | 0.574 | 0.587 | 0.572 |
| 74 | 74 | 25 | 6.354 | 4.959 | 0.694 | 0.716 | 0.725 | 0.706 | 0.716 | 0.725 | 0.712 |
| 75 | 75 | 25 | 6.214 | 4.828 | 0.734 | 0.744 | 0.759 | 0.744 | 0.744 | 0.759 | 0.746 |
| 76 | 76 | 25 | 5.170 | 3.787 | 0.101 | 0.117 | 0.129 | 0.105 | 0.105 | 0.105 | 0.087 |
| 77 | 77 | 25 | 6.522 | 5.122 | 0.661 | 0.684 | 0.701 | 0.662 | 0.684 | 0.701 | 0.682 |
| 78 | 78 | 25 | 5.024 | 3.451 | 0.056 | 0.086 | 0.111 | 0.090 | 0.101 | 0.106 | 0.052 |
| 79 | 79 | 25 | 6.299 | 4.913 | 0.709 | 0.722 | 0.746 | 0.718 | 0.722 | 0.746 | 0.726 |
| 80 | 80 | 25 | 5.365 | 3.824 | 0.058 | 0.086 | 0.119 | 0.080 | 0.093 | 0.113 | 0.080 |
| 81 | 81 | 25 | 6.940 | 5.561 | 0.757 | 0.773 | 0.787 | 0.757 | 0.773 | 0.787 | 0.772 |
| 82 | 82 | 25 | 5.510 | 4.131 | 0.674 | 0.681 | 0.709 | 0.679 | 0.680 | 0.681 | 0.661 |
| 83 | 83 | 25 | 5.031 | 3.548 | 0.072 | 0.089 | 0.114 | 0.093 | 0.093 | 0.096 | 0.057 |
| 84 | 84 | 25 | 4.774 | 3.565 | 0.226 | 0.276 | 0.310 | 0.172 | 0.172 | 0.172 | 0.114 |
| 85 | 85 | 25 | 5.845 | 4.433 | 0.635 | 0.651 | 0.668 | 0.655 | 0.655 | 0.668 | 0.651 |
| 86 | 86 | 25 | 5.417 | 3.946 | 0.434 | 0.511 | 0.578 | 0.534 | 0.534 | 0.534 | 0.463 |
| 87 | 87 | 25 | 6.271 | 4.871 | 0.652 | 0.672 | 0.684 | 0.664 | 0.672 | 0.684 | 0.669 |
| 88 | 88 | 25 | 5.264 | 3.803 | 0.309 | 0.380 | 0.426 | 0.381 | 0.381 | 0.381 | 0.305 |
| 89 | 89 | 25 | 6.460 | 5.077 | 0.757 | 0.778 | 0.792 | 0.770 | 0.778 | 0.792 | 0.776 |
| 90 | 90 | 25 | 7.682 | 6.388 | 0.776 | 0.786 | 0.794 | 0.776 | 0.786 | 0.794 | 0.785 |
| 91 | 91 | 25 | 5.681 | 4.250 | 0.524 | 0.553 | 0.595 | 0.563 | 0.563 | 0.577 | 0.550 |
| 92 | 92 | 25 | 6.027 | 4.608 | 0.516 | 0.535 | 0.554 | 0.534 | 0.535 | 0.554 | 0.535 |
| 93 | 93 | 25 | 5.175 | 3.627 | 0.182 | 0.247 | 0.306 | 0.274 | 0.279 | 0.289 | 0.187 |
| 94 | 94 | 25 | 5.146 | 3.608 | 0.233 | 0.306 | 0.377 | 0.345 | 0.345 | 0.351 | 0.224 |
| 95 | 95 | 25 | 6.342 | 4.948 | 0.706 | 0.721 | 0.742 | 0.719 | 0.721 | 0.742 | 0.723 |
| 96 | 96 | 25 | 7.109 | 5.715 | 0.756 | 0.768 | 0.782 | 0.756 | 0.768 | 0.782 | 0.768 |
| 97 | 97 | 25 | 5.374 | 3.974 | 0.524 | 0.564 | 0.597 | 0.551 | 0.551 | 0.551 | 0.503 |
| 98 | 98 | 25 | 5.458 | 3.962 | 0.333 | 0.400 | 0.457 | 0.423 | 0.423 | 0.427 | 0.371 |
| 99 | 99 | 25 | 5.714 | 4.319 | 0.653 | 0.666 | 0.680 | 0.671 | 0.671 | 0.671 | 0.662 |
| 100 | 100 | 25 | 6.456 | 5.063 | 0.633 | 0.648 | 0.659 | 0.636 | 0.648 | 0.659 | 0.647 |
| 101 | 101 | 25 | 5.553 | 4.122 | 0.485 | 0.512 | 0.545 | 0.524 | 0.524 | 0.524 | 0.502 |
| 102 | 102 | 25 | 5.227 | 3.819 | 0.211 | 0.240 | 0.272 | 0.228 | 0.228 | 0.228 | 0.193 |
| 103 | 103 | 25 | 5.117 | 3.648 | 0.098 | 0.118 | 0.142 | 0.121 | 0.121 | 0.121 | 0.083 |
| 104 | 104 | 25 | 5.459 | 3.987 | 0.495 | 0.575 | 0.656 | 0.609 | 0.609 | 0.609 | 0.538 |
| 105 | 105 | 25 | 6.697 | 5.310 | 0.716 | 0.735 | 0.744 | 0.716 | 0.735 | 0.744 | 0.732 |
| 106 | 106 | 25 | 5.404 | 3.983 | 0.392 | 0.421 | 0.443 | 0.415 | 0.416 | 0.416 | 0.384 |
| 107 | 107 | 25 | 5.020 | 3.790 | 0.127 | 0.137 | 0.147 | 0.097 | 0.097 | 0.097 | 0.085 |
| 108 | 108 | 25 | 5.232 | 3.806 | 0.408 | 0.483 | 0.550 | 0.473 | 0.473 | 0.473 | 0.379 |
| 109 | 109 | 25 | 4.884 | 3.460 | 0.445 | 0.585 | 0.736 | 0.557 | 0.559 | 0.564 | 0.295 |
| 110 | 110 | 25 | 7.209 | 5.824 | 0.819 | 0.838 | 0.860 | 0.819 | 0.838 | 0.860 | 0.839 |
| 111 | 111 | 25 | 4.995 | 3.385 | 0.043 | 0.060 | 0.079 | 0.074 | 0.075 | 0.081 | 0.035 |
| 112 | 112 | 25 | 7.218 | 5.848 | 0.749 | 0.760 | 0.770 | 0.749 | 0.760 | 0.770 | 0.760 |
| 113 | 113 | 25 | 5.379 | 3.893 | 0.391 | 0.471 | 0.550 | 0.500 | 0.500 | 0.506 | 0.422 |
| 114 | 114 | 25 | 5.644 | 4.198 | 0.495 | 0.553 | 0.609 | 0.558 | 0.558 | 0.581 | 0.539 |
| 115 | 115 | 25 | 4.938 | 3.444 | 0.031 | 0.039 | 0.052 | 0.042 | 0.042 | 0.044 | 0.023 |
| 116 | 116 | 25 | 5.218 | 3.663 | 0.041 | 0.054 | 0.066 | 0.059 | 0.060 | 0.063 | 0.043 |
| 117 | 117 | 25 | 5.067 | 3.540 | 0.163 | 0.215 | 0.263 | 0.238 | 0.238 | 0.238 | 0.142 |
| 118 | 118 | 25 | 5.291 | 3.731 | 0.043 | 0.066 | 0.086 | 0.061 | 0.073 | 0.082 | 0.055 |
| 119 | 119 | 25 | 6.478 | 5.096 | 0.803 | 0.812 | 0.829 | 0.811 | 0.812 | 0.829 | 0.815 |
| 120 | 120 | 25 | 6.317 | 4.911 | 0.590 | 0.603 | 0.625 | 0.598 | 0.603 | 0.625 | 0.606 |
| 121 | 121 | 25 | 6.133 | 4.745 | 0.726 | 0.739 | 0.749 | 0.738 | 0.739 | 0.749 | 0.738 |
| 122 | 122 | 25 | 6.317 | 4.926 | 0.694 | 0.725 | 0.729 | 0.711 | 0.725 | 0.729 | 0.716 |
| 123 | 123 | 25 | 7.339 | 5.987 | 0.839 | 0.854 | 0.865 | 0.839 | 0.854 | 0.865 | 0.853 |
| 124 | 124 | 25 | 5.021 | 3.481 | 0.237 | 0.331 | 0.402 | 0.370 | 0.373 | 0.373 | 0.199 |
| 125 | 125 | 25 | 5.581 | 4.175 | 0.596 | 0.616 | 0.636 | 0.622 | 0.622 | 0.622 | 0.602 |
| 126 | 126 | 25 | 5.090 | 3.574 | 0.020 | 0.025 | 0.033 | 0.027 | 0.027 | 0.029 | 0.019 |
| 127 | 127 | 25 | 5.241 | 3.940 | 0.544 | 0.562 | 0.592 | 0.485 | 0.485 | 0.485 | 0.449 |
| 128 | 128 | 25 | 5.048 | 3.828 | 0.592 | 0.626 | 0.669 | 0.457 | 0.457 | 0.457 | 0.398 |
| 129 | 129 | 25 | 6.080 | 4.680 | 0.636 | 0.653 | 0.673 | 0.647 | 0.653 | 0.673 | 0.654 |
| 130 | 130 | 25 | 5.070 | 3.836 | 0.659 | 0.703 | 0.746 | 0.526 | 0.526 | 0.526 | 0.459 |
| 131 | 131 | 25 | 5.234 | 3.716 | 0.309 | 0.398 | 0.473 | 0.435 | 0.435 | 0.438 | 0.313 |
| 132 | 132 | 25 | 7.644 | 6.353 | 0.863 | 0.887 | 0.893 | 0.863 | 0.887 | 0.893 | 0.881 |
| 133 | 133 | 25 | 4.904 | 3.326 | 0.098 | 0.138 | 0.176 | 0.166 | 0.167 | 0.170 | 0.073 |
| 134 | 134 | 25 | 6.008 | 4.598 | 0.728 | 0.761 | 0.793 | 0.761 | 0.761 | 0.793 | 0.761 |
| 135 | 135 | 25 | 5.235 | 3.713 | 0.051 | 0.065 | 0.079 | 0.070 | 0.070 | 0.072 | 0.052 |
| 136 | 136 | 25 | 5.187 | 3.681 | 0.128 | 0.160 | 0.195 | 0.172 | 0.172 | 0.174 | 0.124 |
| 137 | 137 | 25 | 7.546 | 6.193 | 0.780 | 0.786 | 0.803 | 0.780 | 0.786 | 0.803 | 0.790 |
| 138 | 138 | 25 | 4.689 | 3.477 | 0.255 | 0.310 | 0.358 | 0.194 | 0.194 | 0.194 | 0.110 |
| 139 | 139 | 25 | 5.321 | 3.869 | 0.473 | 0.570 | 0.643 | 0.579 | 0.579 | 0.579 | 0.487 |
| 140 | 140 | 25 | 6.462 | 5.072 | 0.668 | 0.677 | 0.692 | 0.668 | 0.677 | 0.692 | 0.679 |
| 141 | 141 | 25 | 7.638 | 6.335 | 0.785 | 0.795 | 0.811 | 0.785 | 0.795 | 0.811 | 0.797 |
| 142 | 142 | 25 | 7.431 | 6.098 | 0.808 | 0.823 | 0.837 | 0.808 | 0.823 | 0.837 | 0.822 |
| 143 | 143 | 25 | 5.231 | 3.716 | 0.125 | 0.156 | 0.194 | 0.168 | 0.168 | 0.176 | 0.127 |
| 144 | 144 | 25 | 5.249 | 3.821 | 0.382 | 0.455 | 0.516 | 0.445 | 0.445 | 0.445 | 0.360 |
| 145 | 145 | 25 | 5.335 | 3.793 | 0.027 | 0.040 | 0.048 | 0.038 | 0.043 | 0.046 | 0.034 |
| 146 | 146 | 25 | 5.083 | 3.547 | 0.041 | 0.058 | 0.081 | 0.061 | 0.064 | 0.074 | 0.041 |
| 147 | 147 | 25 | 5.200 | 3.659 | 0.212 | 0.316 | 0.397 | 0.310 | 0.353 | 0.375 | 0.237 |
| 148 | 148 | 25 | 7.307 | 5.919 | 0.724 | 0.745 | 0.757 | 0.724 | 0.745 | 0.757 | 0.742 |
| 149 | 149 | 25 | 5.150 | 3.602 | 0.062 | 0.093 | 0.123 | 0.093 | 0.104 | 0.115 | 0.069 |
| 150 | 150 | 25 | 5.242 | 3.759 | 0.398 | 0.486 | 0.576 | 0.517 | 0.517 | 0.517 | 0.388 |
| 151 | 151 | 25 | 5.209 | 3.698 | 0.083 | 0.103 | 0.132 | 0.111 | 0.111 | 0.119 | 0.084 |
| 152 | 152 | 25 | 5.097 | 3.604 | 0.244 | 0.302 | 0.414 | 0.325 | 0.325 | 0.362 | 0.219 |
| 153 | 153 | 25 | 7.431 | 6.054 | 0.782 | 0.804 | 0.816 | 0.782 | 0.804 | 0.816 | 0.801 |
| 154 | 154 | 25 | 7.304 | 5.932 | 0.807 | 0.842 | 0.856 | 0.807 | 0.842 | 0.856 | 0.835 |
| 155 | 155 | 25 | 6.991 | 5.585 | 0.690 | 0.705 | 0.714 | 0.690 | 0.705 | 0.714 | 0.703 |
| 156 | 156 | 25 | 4.990 | 3.505 | 0.301 | 0.452 | 0.544 | 0.425 | 0.465 | 0.466 | 0.255 |
| 157 | 157 | 25 | 5.558 | 4.116 | 0.543 | 0.584 | 0.634 | 0.605 | 0.606 | 0.606 | 0.570 |
| 158 | 158 | 25 | 5.019 | 3.608 | 0.439 | 0.519 | 0.634 | 0.499 | 0.499 | 0.499 | 0.325 |
| 159 | 159 | 25 | 5.599 | 4.188 | 0.541 | 0.563 | 0.589 | 0.568 | 0.569 | 0.569 | 0.556 |
| 160 | 160 | 25 | 6.524 | 5.147 | 0.768 | 0.777 | 0.790 | 0.771 | 0.777 | 0.790 | 0.778 |
| 161 | 161 | 25 | 5.216 | 3.787 | 0.345 | 0.414 | 0.469 | 0.402 | 0.402 | 0.402 | 0.321 |
| 162 | 162 | 25 | 5.336 | 3.909 | 0.018 | 0.024 | 0.029 | 0.020 | 0.022 | 0.025 | 0.020 |
| 163 | 163 | 25 | 5.742 | 4.313 | 0.530 | 0.587 | 0.611 | 0.574 | 0.586 | 0.588 | 0.562 |
| 164 | 164 | 25 | 5.311 | 3.773 | 0.097 | 0.124 | 0.155 | 0.135 | 0.135 | 0.145 | 0.109 |
| 165 | 165 | 25 | 5.317 | 3.994 | 0.540 | 0.562 | 0.590 | 0.503 | 0.503 | 0.503 | 0.472 |
| 166 | 166 | 25 | 5.452 | 3.946 | 0.457 | 0.561 | 0.655 | 0.604 | 0.604 | 0.622 | 0.520 |
| 167 | 167 | 25 | 5.396 | 3.886 | 0.246 | 0.327 | 0.389 | 0.327 | 0.346 | 0.363 | 0.295 |
| 168 | 168 | 25 | 5.296 | 3.723 | 0.040 | 0.053 | 0.065 | 0.058 | 0.060 | 0.063 | 0.044 |
| 169 | 169 | 25 | 5.179 | 3.641 | 0.184 | 0.250 | 0.326 | 0.272 | 0.278 | 0.304 | 0.192 |
| 170 | 170 | 25 | 5.207 | 3.681 | 0.188 | 0.243 | 0.301 | 0.266 | 0.266 | 0.278 | 0.190 |
| 171 | 171 | 25 | 5.185 | 3.630 | 0.143 | 0.217 | 0.315 | 0.215 | 0.247 | 0.301 | 0.173 |
| 172 | 172 | 25 | 5.362 | 3.980 | 0.343 | 0.361 | 0.382 | 0.343 | 0.343 | 0.343 | 0.323 |
| 173 | 173 | 25 | 6.343 | 4.946 | 0.699 | 0.716 | 0.733 | 0.708 | 0.716 | 0.726 | 0.714 |
| 174 | 174 | 25 | 5.510 | 4.017 | 0.358 | 0.435 | 0.506 | 0.452 | 0.454 | 0.476 | 0.418 |
| 175 | 175 | 25 | 5.196 | 3.662 | 0.138 | 0.185 | 0.218 | 0.200 | 0.202 | 0.202 | 0.141 |
| 176 | 176 | 25 | 5.180 | 3.629 | 0.135 | 0.208 | 0.254 | 0.201 | 0.234 | 0.241 | 0.150 |
| 177 | 177 | 25 | 5.235 | 3.694 | 0.107 | 0.136 | 0.170 | 0.151 | 0.151 | 0.159 | 0.112 |
| 178 | 178 | 25 | 5.194 | 3.654 | 0.161 | 0.217 | 0.269 | 0.236 | 0.241 | 0.252 | 0.166 |
| 179 | 179 | 25 | 5.951 | 4.542 | 0.634 | 0.649 | 0.673 | 0.653 | 0.653 | 0.662 | 0.648 |
| 180 | 180 | 25 | 7.689 | 6.392 | 0.708 | 0.725 | 0.738 | 0.708 | 0.725 | 0.738 | 0.724 |
| 181 | 181 | 25 | 5.102 | 3.652 | 0.424 | 0.544 | 0.629 | 0.538 | 0.538 | 0.538 | 0.366 |
| 182 | 182 | 25 | 5.970 | 4.560 | 0.640 | 0.664 | 0.692 | 0.664 | 0.664 | 0.692 | 0.665 |
| 183 | 183 | 25 | 5.345 | 3.812 | 0.028 | 0.039 | 0.058 | 0.037 | 0.041 | 0.054 | 0.037 |
| 184 | 184 | 25 | 5.825 | 4.394 | 0.590 | 0.638 | 0.679 | 0.646 | 0.647 | 0.653 | 0.630 |
| 185 | 185 | 25 | 5.937 | 4.559 | 0.730 | 0.735 | 0.747 | 0.735 | 0.735 | 0.747 | 0.734 |
| 186 | 186 | 25 | 5.564 | 4.104 | 0.452 | 0.532 | 0.608 | 0.529 | 0.542 | 0.574 | 0.513 |
| 187 | 187 | 25 | 5.775 | 4.359 | 0.509 | 0.522 | 0.532 | 0.522 | 0.522 | 0.532 | 0.519 |
| 188 | 188 | 25 | 6.362 | 4.975 | 0.724 | 0.741 | 0.761 | 0.729 | 0.741 | 0.761 | 0.742 |
| 189 | 189 | 25 | 6.296 | 4.908 | 0.769 | 0.791 | 0.811 | 0.769 | 0.791 | 0.807 | 0.789 |
| 190 | 190 | 25 | 5.420 | 3.925 | 0.296 | 0.355 | 0.444 | 0.375 | 0.375 | 0.411 | 0.340 |
| 191 | 191 | 25 | 5.353 | 3.845 | 0.342 | 0.433 | 0.495 | 0.462 | 0.462 | 0.463 | 0.370 |
| 192 | 192 | 25 | 6.218 | 4.824 | 0.746 | 0.766 | 0.787 | 0.764 | 0.766 | 0.787 | 0.766 |
| 193 | 193 | 25 | 6.232 | 4.832 | 0.691 | 0.706 | 0.722 | 0.708 | 0.708 | 0.722 | 0.706 |
| 194 | 194 | 25 | 4.985 | 3.457 | 0.112 | 0.146 | 0.190 | 0.164 | 0.164 | 0.170 | 0.088 |
| 195 | 195 | 25 | 5.733 | 4.308 | 0.521 | 0.537 | 0.548 | 0.545 | 0.545 | 0.548 | 0.530 |
| 196 | 196 | 25 | 5.944 | 4.530 | 0.687 | 0.724 | 0.744 | 0.726 | 0.726 | 0.744 | 0.718 |
| 197 | 197 | 25 | 6.320 | 4.920 | 0.661 | 0.681 | 0.703 | 0.667 | 0.681 | 0.703 | 0.682 |
| 198 | 198 | 25 | 6.745 | 5.358 | 0.677 | 0.694 | 0.698 | 0.677 | 0.694 | 0.698 | 0.690 |
| 199 | 199 | 25 | 6.180 | 4.781 | 0.591 | 0.613 | 0.625 | 0.591 | 0.613 | 0.625 | 0.610 |
| 200 | 200 | 25 | 5.366 | 3.855 | 0.299 | 0.382 | 0.447 | 0.405 | 0.408 | 0.417 | 0.333 |
| 201 | 201 | 25 | 5.575 | 4.133 | 0.602 | 0.678 | 0.739 | 0.688 | 0.688 | 0.704 | 0.647 |
| 202 | 202 | 25 | 6.010 | 4.606 | 0.689 | 0.715 | 0.724 | 0.715 | 0.715 | 0.724 | 0.709 |
| 203 | 203 | 25 | 6.781 | 5.391 | 0.737 | 0.746 | 0.764 | 0.737 | 0.746 | 0.764 | 0.749 |
| 204 | 204 | 25 | 5.368 | 3.831 | 0.100 | 0.126 | 0.154 | 0.134 | 0.136 | 0.146 | 0.115 |
| 205 | 205 | 25 | 6.278 | 4.873 | 0.625 | 0.647 | 0.682 | 0.642 | 0.647 | 0.666 | 0.646 |
| 206 | 206 | 25 | 5.979 | 4.562 | 0.610 | 0.635 | 0.677 | 0.635 | 0.635 | 0.669 | 0.638 |
| 207 | 207 | 25 | 5.220 | 3.918 | 0.361 | 0.381 | 0.403 | 0.321 | 0.321 | 0.321 | 0.303 |
| 208 | 208 | 25 | 4.747 | 3.627 | 0.454 | 0.506 | 0.565 | 0.280 | 0.280 | 0.280 | 0.203 |
| 209 | 209 | 25 | 6.203 | 4.809 | 0.702 | 0.721 | 0.738 | 0.718 | 0.721 | 0.738 | 0.720 |
| 210 | 210 | 25 | 5.362 | 3.833 | 0.045 | 0.060 | 0.070 | 0.060 | 0.064 | 0.065 | 0.052 |
| 211 | 211 | 25 | 5.714 | 4.271 | 0.476 | 0.537 | 0.570 | 0.516 | 0.537 | 0.560 | 0.524 |
| 212 | 212 | 25 | 6.818 | 5.419 | 0.668 | 0.690 | 0.704 | 0.668 | 0.690 | 0.704 | 0.687 |
| 213 | 213 | 25 | 6.017 | 4.622 | 0.653 | 0.670 | 0.691 | 0.665 | 0.670 | 0.691 | 0.671 |
| 214 | 214 | 25 | 5.189 | 3.641 | 0.112 | 0.151 | 0.193 | 0.165 | 0.168 | 0.181 | 0.115 |
| 215 | 215 | 25 | 5.548 | 4.093 | 0.480 | 0.542 | 0.637 | 0.556 | 0.556 | 0.597 | 0.530 |
| 216 | 216 | 25 | 5.891 | 4.496 | 0.725 | 0.737 | 0.746 | 0.745 | 0.745 | 0.746 | 0.733 |
| 217 | 217 | 25 | 6.385 | 4.991 | 0.679 | 0.685 | 0.695 | 0.679 | 0.685 | 0.695 | 0.686 |
| 218 | 218 | 25 | 5.438 | 3.946 | 0.436 | 0.522 | 0.612 | 0.557 | 0.557 | 0.573 | 0.484 |
| 219 | 219 | 25 | 5.815 | 4.378 | 0.617 | 0.654 | 0.678 | 0.669 | 0.669 | 0.672 | 0.648 |
| 220 | 220 | 25 | 5.487 | 4.012 | 0.436 | 0.510 | 0.573 | 0.533 | 0.533 | 0.535 | 0.479 |
| 221 | 221 | 25 | 6.208 | 4.811 | 0.711 | 0.742 | 0.756 | 0.729 | 0.742 | 0.756 | 0.736 |
| 222 | 222 | 25 | 6.429 | 5.041 | 0.668 | 0.686 | 0.698 | 0.675 | 0.686 | 0.698 | 0.684 |
| 223 | 223 | 25 | 5.264 | 3.905 | 0.382 | 0.421 | 0.457 | 0.381 | 0.381 | 0.381 | 0.342 |
| 224 | 224 | 25 | 5.493 | 4.080 | 0.586 | 0.604 | 0.641 | 0.616 | 0.616 | 0.616 | 0.579 |
| 225 | 225 | 25 | 5.248 | 3.747 | 0.247 | 0.303 | 0.382 | 0.326 | 0.326 | 0.347 | 0.251 |
| 226 | 226 | 25 | 6.580 | 5.200 | 0.749 | 0.759 | 0.766 | 0.749 | 0.759 | 0.766 | 0.758 |
| 227 | 227 | 25 | 5.764 | 4.370 | 0.684 | 0.723 | 0.751 | 0.711 | 0.718 | 0.724 | 0.704 |
| 228 | 228 | 25 | 5.818 | 4.414 | 0.681 | 0.703 | 0.717 | 0.707 | 0.708 | 0.709 | 0.696 |
| 229 | 229 | 25 | 5.260 | 3.724 | 0.129 | 0.202 | 0.245 | 0.184 | 0.222 | 0.229 | 0.161 |
| 6 | 230 | 13 | 6.101 | 4.692 | 0.650 | 0.673 | 0.708 | 0.669 | 0.673 | 0.708 | 0.677 |
| 8 | 231 | 13 | 5.511 | 4.093 | 0.419 | 0.430 | 0.444 | 0.433 | 0.433 | 0.433 | 0.421 |
| 10 | 232 | 13 | 6.111 | 4.692 | 0.625 | 0.665 | 0.687 | 0.650 | 0.665 | 0.687 | 0.659 |
| 17 | 233 | 13 | 5.027 | 3.783 | 0.317 | 0.359 | 0.378 | 0.257 | 0.257 | 0.257 | 0.221 |
| 22 | 234 | 13 | 7.667 | 6.377 | 0.811 | 0.827 | 0.838 | 0.811 | 0.827 | 0.838 | 0.826 |
| 28 | 235 | 13 | 5.671 | 4.239 | 0.516 | 0.528 | 0.544 | 0.543 | 0.543 | 0.544 | 0.528 |
| 32 | 236 | 13 | 5.288 | 3.884 | 0.459 | 0.536 | 0.581 | 0.509 | 0.509 | 0.509 | 0.439 |
| 54 | 237 | 13 | 5.292 | 3.946 | 0.424 | 0.452 | 0.493 | 0.411 | 0.411 | 0.411 | 0.388 |
| 55 | 238 | 13 | 5.511 | 4.074 | 0.498 | 0.539 | 0.559 | 0.545 | 0.545 | 0.545 | 0.514 |
| 72 | 239 | 13 | 5.473 | 4.057 | 0.511 | 0.566 | 0.600 | 0.560 | 0.560 | 0.560 | 0.531 |
| 73 | 240 | 13 | 5.377 | 3.993 | 0.455 | 0.470 | 0.498 | 0.455 | 0.456 | 0.456 | 0.425 |
| 75 | 241 | 13 | 5.431 | 4.020 | 0.523 | 0.563 | 0.612 | 0.563 | 0.563 | 0.564 | 0.527 |
| 79 | 242 | 13 | 5.187 | 3.861 | 0.515 | 0.558 | 0.616 | 0.486 | 0.486 | 0.486 | 0.428 |
| 85 | 243 | 13 | 5.526 | 4.082 | 0.488 | 0.524 | 0.555 | 0.539 | 0.539 | 0.539 | 0.512 |
| 89 | 244 | 13 | 5.530 | 4.058 | 0.507 | 0.526 | 0.595 | 0.573 | 0.574 | 0.574 | 0.526 |
| 90 | 245 | 13 | 7.470 | 6.107 | 0.782 | 0.792 | 0.802 | 0.782 | 0.792 | 0.802 | 0.792 |
| 92 | 246 | 13 | 5.373 | 3.990 | 0.426 | 0.448 | 0.469 | 0.428 | 0.428 | 0.428 | 0.400 |
| 95 | 247 | 13 | 5.634 | 4.205 | 0.576 | 0.604 | 0.628 | 0.615 | 0.617 | 0.622 | 0.598 |
| 96 | 248 | 13 | 6.744 | 5.342 | 0.685 | 0.697 | 0.730 | 0.685 | 0.697 | 0.730 | 0.704 |
| 120 | 249 | 13 | 5.975 | 4.563 | 0.542 | 0.552 | 0.583 | 0.551 | 0.552 | 0.583 | 0.559 |
| 101 | 250 | 13 | 5.084 | 3.883 | 0.386 | 0.407 | 0.416 | 0.287 | 0.287 | 0.287 | 0.273 |
| 129 | 251 | 13 | 5.470 | 4.056 | 0.576 | 0.602 | 0.633 | 0.610 | 0.610 | 0.610 | 0.570 |
| 132 | 252 | 13 | 7.292 | 5.897 | 0.754 | 0.795 | 0.818 | 0.754 | 0.795 | 0.818 | 0.789 |
| 110 | 253 | 13 | 6.924 | 5.532 | 0.683 | 0.695 | 0.736 | 0.683 | 0.695 | 0.736 | 0.705 |
| 104 | 254 | 13 | 5.018 | 3.796 | 0.502 | 0.521 | 0.570 | 0.383 | 0.383 | 0.383 | 0.325 |
| 112 | 255 | 13 | 6.665 | 5.264 | 0.617 | 0.659 | 0.676 | 0.617 | 0.659 | 0.676 | 0.651 |
| 105 | 256 | 13 | 5.567 | 4.119 | 0.559 | 0.591 | 0.617 | 0.613 | 0.614 | 0.614 | 0.584 |
| 137 | 257 | 13 | 7.036 | 5.656 | 0.750 | 0.766 | 0.789 | 0.750 | 0.766 | 0.789 | 0.768 |
| 100 | 258 | 13 | 5.451 | 4.002 | 0.458 | 0.498 | 0.550 | 0.517 | 0.517 | 0.517 | 0.474 |
| 184 | 259 | 13 | 5.411 | 4.018 | 0.510 | 0.559 | 0.604 | 0.543 | 0.543 | 0.543 | 0.514 |
| 185 | 260 | 13 | 5.641 | 4.201 | 0.585 | 0.610 | 0.631 | 0.629 | 0.629 | 0.631 | 0.604 |
| 186 | 261 | 13 | 5.358 | 3.930 | 0.421 | 0.463 | 0.523 | 0.468 | 0.468 | 0.468 | 0.416 |
| 187 | 262 | 13 | 5.336 | 4.010 | 0.489 | 0.506 | 0.525 | 0.454 | 0.454 | 0.454 | 0.443 |
| 192 | 263 | 13 | 5.813 | 4.403 | 0.636 | 0.648 | 0.670 | 0.656 | 0.656 | 0.670 | 0.651 |
| 209 | 264 | 13 | 5.747 | 4.325 | 0.561 | 0.589 | 0.651 | 0.585 | 0.589 | 0.651 | 0.597 |
| 216 | 265 | 13 | 5.129 | 3.803 | 0.435 | 0.503 | 0.540 | 0.418 | 0.418 | 0.418 | 0.349 |
| 219 | 266 | 13 | 5.069 | 3.818 | 0.520 | 0.579 | 0.609 | 0.431 | 0.431 | 0.431 | 0.374 |
| 227 | 267 | 13 | 5.449 | 4.021 | 0.534 | 0.608 | 0.652 | 0.606 | 0.607 | 0.607 | 0.563 |
| 19 | 268 | 37 | 7.022 | 5.639 | 0.753 | 0.764 | 0.778 | 0.753 | 0.764 | 0.778 | 0.765 |
| 20 | 269 | 37 | 6.481 | 5.091 | 0.677 | 0.698 | 0.715 | 0.677 | 0.698 | 0.715 | 0.697 |
| 30 | 270 | 37 | 6.851 | 5.455 | 0.742 | 0.757 | 0.784 | 0.742 | 0.757 | 0.784 | 0.761 |
| 33 | 271 | 37 | 7.360 | 6.001 | 0.822 | 0.850 | 0.864 | 0.822 | 0.850 | 0.864 | 0.845 |
| 39 | 272 | 37 | 6.537 | 5.156 | 0.678 | 0.687 | 0.695 | 0.678 | 0.687 | 0.695 | 0.687 |
| 45 | 273 | 37 | 6.709 | 5.318 | 0.727 | 0.746 | 0.761 | 0.727 | 0.746 | 0.761 | 0.745 |
| 46 | 274 | 37 | 6.211 | 4.801 | 0.682 | 0.710 | 0.753 | 0.706 | 0.710 | 0.753 | 0.715 |
| 51 | 275 | 37 | 6.668 | 5.279 | 0.779 | 0.795 | 0.806 | 0.779 | 0.795 | 0.806 | 0.793 |
| 57 | 276 | 37 | 6.229 | 4.826 | 0.627 | 0.639 | 0.657 | 0.638 | 0.639 | 0.657 | 0.641 |
| 59 | 277 | 37 | 6.603 | 5.206 | 0.720 | 0.743 | 0.775 | 0.720 | 0.743 | 0.775 | 0.746 |
| 60 | 278 | 37 | 5.950 | 4.548 | 0.683 | 0.703 | 0.719 | 0.701 | 0.703 | 0.719 | 0.701 |
| 61 | 279 | 37 | 5.849 | 4.432 | 0.546 | 0.569 | 0.588 | 0.570 | 0.570 | 0.588 | 0.565 |
| 74 | 280 | 37 | 6.593 | 5.204 | 0.687 | 0.729 | 0.742 | 0.691 | 0.729 | 0.742 | 0.720 |
| 77 | 281 | 37 | 6.825 | 5.430 | 0.747 | 0.757 | 0.787 | 0.747 | 0.757 | 0.787 | 0.764 |
| 81 | 282 | 37 | 7.160 | 5.760 | 0.705 | 0.739 | 0.766 | 0.705 | 0.739 | 0.766 | 0.737 |
| 82 | 283 | 37 | 5.730 | 4.290 | 0.632 | 0.680 | 0.718 | 0.685 | 0.691 | 0.718 | 0.670 |
| 87 | 284 | 37 | 6.377 | 4.974 | 0.614 | 0.631 | 0.653 | 0.620 | 0.631 | 0.653 | 0.633 |
| 119 | 285 | 37 | 6.845 | 5.471 | 0.795 | 0.816 | 0.831 | 0.795 | 0.816 | 0.831 | 0.814 |
| 180 | 286 | 37 | 7.695 | 6.431 | 0.873 | 0.879 | 0.889 | 0.873 | 0.879 | 0.889 | 0.880 |
| 160 | 287 | 37 | 6.720 | 5.335 | 0.741 | 0.755 | 0.768 | 0.741 | 0.755 | 0.768 | 0.755 |
| 166 | 288 | 37 | 5.660 | 4.221 | 0.516 | 0.561 | 0.588 | 0.563 | 0.563 | 0.575 | 0.547 |
| 154 | 289 | 37 | 7.665 | 6.374 | 0.801 | 0.820 | 0.829 | 0.801 | 0.820 | 0.829 | 0.817 |
| 153 | 290 | 37 | 7.625 | 6.318 | 0.851 | 0.858 | 0.866 | 0.851 | 0.858 | 0.866 | 0.858 |
| 148 | 291 | 37 | 7.535 | 6.199 | 0.753 | 0.770 | 0.774 | 0.753 | 0.770 | 0.774 | 0.766 |
| 140 | 292 | 37 | 6.597 | 5.195 | 0.653 | 0.672 | 0.682 | 0.653 | 0.672 | 0.682 | 0.669 |
| 97 | 293 | 37 | 5.781 | 4.373 | 0.599 | 0.647 | 0.687 | 0.616 | 0.647 | 0.669 | 0.634 |
| 141 | 294 | 37 | 7.679 | 6.401 | 0.859 | 0.878 | 0.887 | 0.859 | 0.878 | 0.887 | 0.875 |
| 134 | 295 | 37 | 6.246 | 4.854 | 0.735 | 0.753 | 0.794 | 0.751 | 0.753 | 0.794 | 0.761 |
| 142 | 296 | 37 | 7.634 | 6.338 | 0.809 | 0.816 | 0.824 | 0.809 | 0.816 | 0.824 | 0.816 |
| 159 | 297 | 37 | 6.158 | 4.753 | 0.625 | 0.638 | 0.662 | 0.640 | 0.640 | 0.662 | 0.642 |
| 99 | 298 | 37 | 6.057 | 4.640 | 0.707 | 0.746 | 0.767 | 0.749 | 0.749 | 0.766 | 0.740 |
| 189 | 299 | 37 | 6.519 | 5.130 | 0.784 | 0.801 | 0.815 | 0.784 | 0.801 | 0.811 | 0.799 |
| 193 | 300 | 37 | 6.487 | 5.089 | 0.698 | 0.729 | 0.746 | 0.706 | 0.729 | 0.746 | 0.724 |
| 195 | 301 | 37 | 6.149 | 4.731 | 0.596 | 0.633 | 0.654 | 0.613 | 0.633 | 0.654 | 0.628 |
| 202 | 302 | 37 | 6.354 | 4.967 | 0.756 | 0.789 | 0.805 | 0.764 | 0.789 | 0.805 | 0.784 |
| 206 | 303 | 37 | 6.374 | 4.965 | 0.684 | 0.713 | 0.725 | 0.684 | 0.713 | 0.725 | 0.707 |
| 211 | 304 | 37 | 6.099 | 4.700 | 0.589 | 0.609 | 0.636 | 0.592 | 0.609 | 0.625 | 0.608 |
| 217 | 305 | 37 | 6.487 | 5.082 | 0.681 | 0.701 | 0.715 | 0.681 | 0.701 | 0.715 | 0.699 |
| 221 | 306 | 37 | 6.611 | 5.228 | 0.728 | 0.748 | 0.765 | 0.730 | 0.748 | 0.765 | 0.747 |
| 224 | 307 | 37 | 5.961 | 4.533 | 0.671 | 0.725 | 0.737 | 0.723 | 0.725 | 0.737 | 0.711 |
| 228 | 308 | 37 | 5.993 | 4.590 | 0.652 | 0.691 | 0.733 | 0.656 | 0.691 | 0.723 | 0.689 |
| 3 | 309 | 49 | 6.390 | 4.985 | 0.680 | 0.714 | 0.758 | 0.684 | 0.714 | 0.758 | 0.718 |
| 7 | 310 | 49 | 7.652 | 6.350 | 0.784 | 0.802 | 0.813 | 0.784 | 0.802 | 0.813 | 0.800 |
| 12 | 311 | 49 | 6.289 | 4.883 | 0.684 | 0.706 | 0.728 | 0.697 | 0.706 | 0.728 | 0.706 |
| 24 | 312 | 49 | 7.311 | 5.932 | 0.714 | 0.730 | 0.745 | 0.714 | 0.730 | 0.745 | 0.730 |
| 27 | 313 | 49 | 6.796 | 5.388 | 0.672 | 0.712 | 0.728 | 0.672 | 0.712 | 0.728 | 0.704 |
| 36 | 314 | 49 | 6.309 | 4.913 | 0.747 | 0.768 | 0.823 | 0.757 | 0.768 | 0.823 | 0.779 |
| 37 | 315 | 49 | 6.724 | 5.336 | 0.756 | 0.770 | 0.792 | 0.756 | 0.770 | 0.792 | 0.773 |
| 40 | 316 | 49 | 6.283 | 4.878 | 0.628 | 0.652 | 0.672 | 0.628 | 0.652 | 0.668 | 0.649 |
| 47 | 317 | 49 | 6.559 | 5.168 | 0.766 | 0.774 | 0.805 | 0.766 | 0.774 | 0.805 | 0.782 |
| 50 | 318 | 49 | 6.590 | 5.192 | 0.708 | 0.738 | 0.761 | 0.708 | 0.738 | 0.761 | 0.735 |
| 62 | 319 | 49 | 6.485 | 5.089 | 0.645 | 0.668 | 0.680 | 0.652 | 0.668 | 0.680 | 0.664 |
| 64 | 320 | 49 | 6.956 | 5.583 | 0.782 | 0.798 | 0.805 | 0.782 | 0.798 | 0.805 | 0.795 |
| 66 | 321 | 49 | 6.396 | 5.011 | 0.710 | 0.717 | 0.727 | 0.714 | 0.717 | 0.727 | 0.718 |
| 68 | 322 | 49 | 6.722 | 5.331 | 0.680 | 0.694 | 0.712 | 0.680 | 0.694 | 0.712 | 0.695 |
| 70 | 323 | 49 | 6.877 | 5.464 | 0.662 | 0.693 | 0.712 | 0.662 | 0.693 | 0.712 | 0.689 |
| 71 | 324 | 49 | 6.054 | 4.649 | 0.621 | 0.658 | 0.681 | 0.632 | 0.658 | 0.669 | 0.649 |
| 91 | 325 | 49 | 6.011 | 4.603 | 0.580 | 0.603 | 0.635 | 0.592 | 0.603 | 0.622 | 0.602 |
| 121 | 326 | 49 | 6.827 | 5.436 | 0.752 | 0.771 | 0.796 | 0.752 | 0.771 | 0.796 | 0.773 |
| 173 | 327 | 49 | 6.837 | 5.439 | 0.699 | 0.734 | 0.754 | 0.699 | 0.734 | 0.754 | 0.729 |
| 125 | 328 | 49 | 6.413 | 5.012 | 0.673 | 0.698 | 0.749 | 0.685 | 0.698 | 0.749 | 0.707 |
| 123 | 329 | 49 | 7.478 | 6.141 | 0.817 | 0.848 | 0.870 | 0.817 | 0.848 | 0.870 | 0.845 |
| 163 | 330 | 49 | 6.313 | 4.905 | 0.596 | 0.620 | 0.652 | 0.596 | 0.620 | 0.648 | 0.621 |
| 114 | 331 | 49 | 6.403 | 4.989 | 0.582 | 0.613 | 0.627 | 0.582 | 0.613 | 0.626 | 0.607 |
| 155 | 332 | 49 | 7.109 | 5.725 | 0.748 | 0.763 | 0.783 | 0.748 | 0.763 | 0.783 | 0.765 |
| 179 | 333 | 49 | 6.367 | 4.960 | 0.696 | 0.724 | 0.749 | 0.696 | 0.724 | 0.745 | 0.722 |
| 122 | 334 | 49 | 7.111 | 5.713 | 0.775 | 0.790 | 0.821 | 0.775 | 0.790 | 0.821 | 0.796 |
| 157 | 335 | 49 | 6.070 | 4.676 | 0.644 | 0.678 | 0.716 | 0.645 | 0.678 | 0.703 | 0.675 |
| 182 | 336 | 49 | 6.561 | 5.169 | 0.692 | 0.729 | 0.748 | 0.694 | 0.729 | 0.748 | 0.723 |
| 188 | 337 | 49 | 6.620 | 5.228 | 0.743 | 0.757 | 0.790 | 0.743 | 0.757 | 0.790 | 0.763 |
| 196 | 338 | 49 | 6.295 | 4.898 | 0.720 | 0.744 | 0.772 | 0.726 | 0.744 | 0.772 | 0.745 |
| 197 | 339 | 49 | 6.600 | 5.211 | 0.732 | 0.740 | 0.768 | 0.732 | 0.740 | 0.768 | 0.747 |
| 198 | 340 | 49 | 6.914 | 5.528 | 0.719 | 0.745 | 0.760 | 0.719 | 0.745 | 0.760 | 0.741 |
| 199 | 341 | 49 | 6.416 | 5.015 | 0.642 | 0.659 | 0.706 | 0.642 | 0.659 | 0.706 | 0.669 |
| 201 | 342 | 49 | 6.130 | 4.722 | 0.612 | 0.645 | 0.664 | 0.612 | 0.645 | 0.664 | 0.640 |
| 203 | 343 | 49 | 7.014 | 5.626 | 0.767 | 0.794 | 0.826 | 0.767 | 0.794 | 0.826 | 0.795 |
| 205 | 344 | 49 | 6.555 | 5.140 | 0.601 | 0.623 | 0.639 | 0.601 | 0.623 | 0.639 | 0.621 |
| 212 | 345 | 49 | 7.210 | 5.825 | 0.792 | 0.819 | 0.831 | 0.792 | 0.819 | 0.831 | 0.814 |
| 213 | 346 | 49 | 6.587 | 5.184 | 0.695 | 0.714 | 0.736 | 0.695 | 0.714 | 0.736 | 0.715 |
| 215 | 347 | 49 | 6.162 | 4.760 | 0.627 | 0.667 | 0.698 | 0.629 | 0.667 | 0.691 | 0.662 |
| 222 | 348 | 49 | 6.708 | 5.331 | 0.740 | 0.744 | 0.770 | 0.741 | 0.744 | 0.770 | 0.751 |
| 226 | 349 | 49 | 7.031 | 5.642 | 0.764 | 0.787 | 0.815 | 0.764 | 0.787 | 0.815 | 0.789 |

# References

[1] A. J. Genot, D. Y. Zhang, J. Bath, A. J. Turberfield, *J. Am. Chem. Soc.* **2011**, *133*, 2177-2182.

[2] R. R. Machinek, T. E. Ouldridge, N. E. Haley, J. Bath, A. J. Turberfield, *Nat. Commun.* **2014**, *5*, 5324.
